# Synthesis of Enantiomerically Enriched Protected 2-Amino-, 2,3-Diamino- and 2-Amino-3-Hydroxypropylphosphonates

**DOI:** 10.3390/molecules28031466

**Published:** 2023-02-02

**Authors:** Aleksandra Trocha, Dorota G. Piotrowska, Iwona E. Głowacka

**Affiliations:** Bioorganic Chemistry Laboratory, Faculty of Pharmacy, Medical University of Lodz, Muszynskiego 1, 90-151 Lodz, Poland

**Keywords:** aziridinephosphonates, aziridine ring-opening, aminopropylphosphonates, aminohydroxypropylphophonates

## Abstract

Simple and efficient strategies for the syntheses of enantiomerically enriched functionalized diethyl 2-amino-, 2,3-diamino- and 2-amino-3-hydroxypropylphosphonates have been developed starting from, respectively, *N*-protected (aziridin-2-yl)methylphosphonates, employing a regioselective aziridine ring-opening reaction with corresponding nucleophiles. Diethyl (*R*)- and (*S*)-2-(*N*-Boc-amino)propylphosphonates were obtained via direct regiospecific hydrogenolysis of the respective enantiomer of (*R*)- and (*S*)-*N*-Boc-(aziridin-2-yl)methylphosphonates. N-Boc-protected (*R*)- and (*S*)-2,3-diaminopropylphosphonates were synthesized from (*R*)- and (*S*)-*N*-Bn-(aziridin-2-yl)methylphosphonates via a regiospecific ring-opening reaction with neat trimethylsilyl azide and subsequent reduction of (*R*)- and (*S*)-2-(*N*-Boc-amino)-3-azidopropylphosphonates using triphenylphosphine. On the other hand, treatment of the corresponding (*R*)- and (*S*)-*N*-Bn-(aziridin-2-yl)methylphosphonates with glacial acetic acid led regiospecifically to the formation of (*R*)- and (S)-2-(*N*-Bn-amino)-3-acetoxypropylphosphonates.

## 1. Introduction

**Figure 1:**
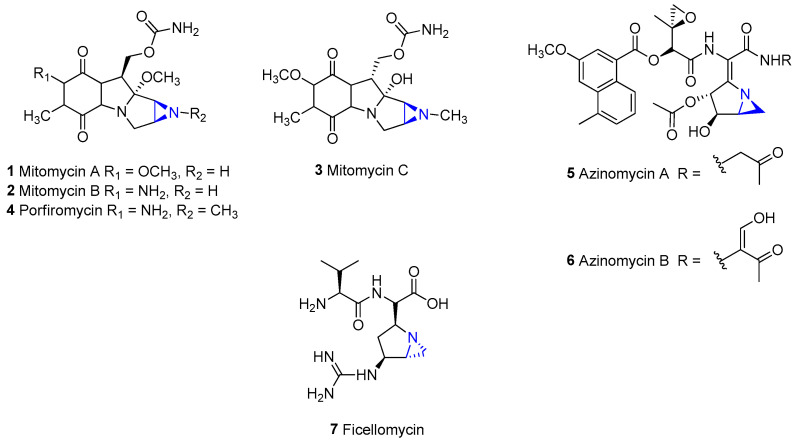
Structures of biologically active compounds bearing aziridine ring.

The usefulness of aziridines for both organic and medicinal chemistry has been intensively explored over the decades [1,2,3,4,5,6,7,8,9,10,11]. The aziridine ring has been found in the structure of biologically relevant compounds, such as naturally occurring mitomycins A, B, C **1**–**3** [12,13], porfiromycin **4** [14], azinomycins A and B **5**–**6** [15] showing antiproliferative activity or ficellomycin **7** [16] exhibiting antibacterial activity (Figure 1). On the other hand, *C*-substituted aziridines can serve as useful building blocks for the synthesis of a wide range of various compounds containing amino functions, e.g., amino acids, amino alcohols, and diamines. Due to the high strain of the three-membered ring, aziridines readily undergo highly regio- and stereospecific ring-opening reactions with a broad spectrum of nucleophiles that makes them powerful tools in synthetic chemistry. However, the regioselectivity of the ring-opening of functionalized aziridines depends on the nature of the substituent attached to the nitrogen atom [17]. In general, activated aziridines, i.e., aziridines bearing an electro-withdrawing group at nitrogen e.g., acyl, sulfonyl, or phosphoryl, undergo ring cleavage reactions on the less-hindered carbon atom under relatively mild conditions. The second class of aziridines constitutes non-activated aziridines that possess an electron-donating substituent e.g., alkyl group and they must be activated by the formation of aziridinium ion or its equivalent prior to ring opening while regioselectivity of these reactions depends on the nature of nucleophile, electrophile, and substituent present at the C2 position [8].

**Figure 2:**
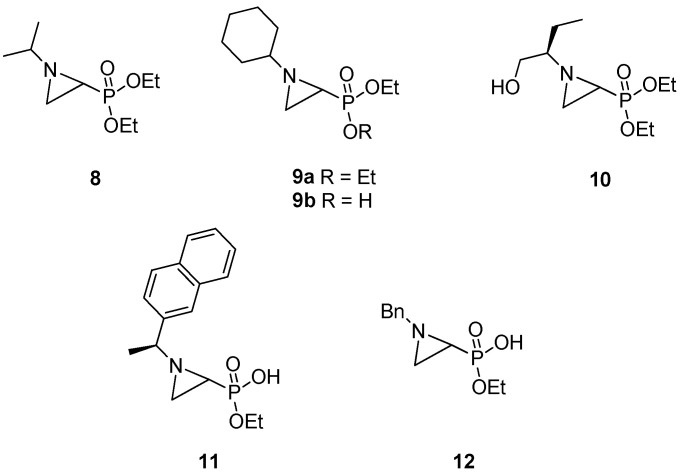
Structures of biologically active aziridine-2-phosphonates.

Among the large variety of structurally diverse compounds containing an aziridine framework, aziridinephosphonates are of special interest. Due to the presence of both an aziridine ring and a phosphonic function which mimics the carboxylate group from amino acids, they can exhibit interesting biological properties. For example, compounds **8**, **9a**, and **10** have been reported to possess high antibacterial activity, comparable with Ampicillin and Streptomycin [18], whereas aziridines **9b** and **11**–**12** were found active against HCT-116 colon cancer cells (Figure 2) [19].

**Figure 3:**
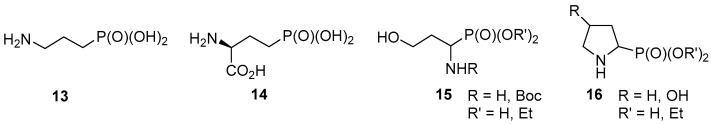
Examples of phosphonate analogues of amino acids.

On the other hand, aziridinephosphonates can be successfully employed as convenient substrates for the synthesis of aminophosphonates, which have attracted tremendous importance in medicinal chemistry as they are structurally related to amino acids and thereby can act as false substrates or inhibitors for enzymes or receptors [20,21,22]. Among the various functionalized phosphonates, α-amino and β-aminophosphonates belong to the well-studied group of compounds. Over decades, numerous phosphonate analogs of amino acids have been synthesized and found application in both organic and medicinal chemistry, including the preparation of more complex compounds (Figure 3). For example, 3-aminopropylphosphonic acid **13** (3-APPA) is an agonist of GABA_B_ receptor [23], while L-2-amino-4-phosphonobutyric acid **14** (L-AP4) was found to be a selective group III metabotropic glutamate receptor agonist that acts at mGlu4, mGlu8, mGlu6, and mGlu7 receptors [24]. Compounds **15** have been designed as phosphonate analogs of homoserine, an important amino acid involved in physiologically relevant transformation [25]. Phosphonate proline analogs **16** were found to serve as chiral catalysts for aldol reaction [26], and some of them have exhibited promising biological activity when incorporated in phosphonodipeptide structures [27].

**Figure 4:**
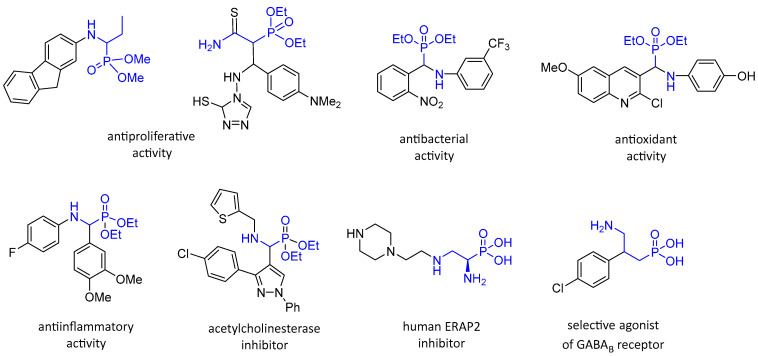
Examples of biologically active aminophosphonates.

Compounds containing an aminophosphonic unit display a wide spectrum of biological activity (Figure 4), e.g., antiproliferative [28,29,30,31], antimicrobial [32,33,34], antiviral [35,36], antioxidant [37,38,39,40], anti-inflammatory [37,41,42] and they act as inhibitors of various enzymes such as acetylcholinesterase [43,44,45], aminopeptidases [46,47] or α-glucosidase [48].

**Scheme 1:**
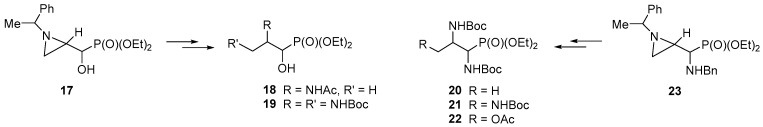
Previous achievements in the synthesis of functionalized α-hydroxyphosphonates **18** and **19** as well as α-aminophosphonates **20**, **21** and **22**.

Several years ago, hydroxyl-[1-(1-phenylethyl)aziridin-2-yl]methylphosphonates **17** [49,50] were successfully obtained in our research group and applied to the preparation of functionalized stereoisomers of α-hydroxyphosphonates **18** and **19**. A similar strategy was also used for the synthesis of functionalized α-aminophosphonates **20**, **21**, and **22** using benzylamino-[1-(1-phenylethyl)aziridin-2-yl]methylphosphonates **23** [51,52] as starting materials (Scheme 1).

**Scheme 2:**
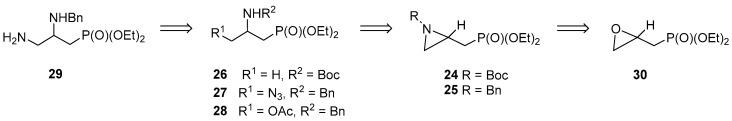
Retrosynthesis of enantiomerically enriched aminophosphonates **26**–**29**.

As a continuation of our studies on the synthesis of phosphonates functionalized with amino and hydroxy groups [49,50,51,52], a new series of enantiomerically enriched (*R*)- and (*S*)-diethyl 2-amino-, 2,3-diamino- and 2-amino-3-hydroxypropylphosphonates has been designed. Our synthetic strategy includes as a key step aziridine ring-opening reaction in both activated (**24**; R = Boc) and non-activated (**25**; R = Bn) (aziridin-2-yl)methylphosphonates, with selected nucleophiles (Scheme 2).

## 2. Results and Discussion

**Scheme 3:**
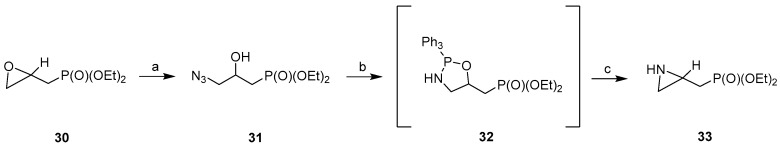
Synthesis of the diethyl (aziridin-2-yl)methylphosphonate (*RS*)-**33**. Reagents and conditions: (**a**) NaN_3_, (NH_4_)_2_SO_4_, MeOH, reflux, 4 h, 95%; (**b**) PPh_3_, toluene, rt, 2 h; (**c**) reflux, 1 h, 53% from two steps.

Elaboration of synthetic procedures for the preparation of designed phosphonates **26**–**29** was preceded by the optimization of reaction conditions using racemic substrates. In this way, the synthesis of racemic (aziridin-2-yl)methylphosphonate (*RS*)-**33** was accomplished starting from 2,3-epoxypropylphosphonate (*RS*)-**30** (Scheme 3). The ring-opening reaction of epoxide (*RS*)-**30** with sodium azide in the presence of ammonium sulfate led to the formation of diethyl 3-azido-2-hydroxypropylphosphonate (*RS*)-**31** [53], which was obtained in 95% yield after filtration through a Celite. Transformation of 3-azido-2-hydroxypropylphosphonate (*RS*)-**31** into (*RS*)-**33** was accomplished in a two-step procedure including the formation of unstable oxazaphospholidine intermediate (*RS*)-**32** and its conversion into (aziridin-2-yl)methylphosphonate (*RS*)-**33** [54]. To remove triphenylphosphine oxide formed as a by-product, the crude reaction mixture was passed through a silica gel, and (aziridin-2-yl)methylphosphonate (*RS*)-**33** was immediately used for the next steps.

**Scheme 4:**
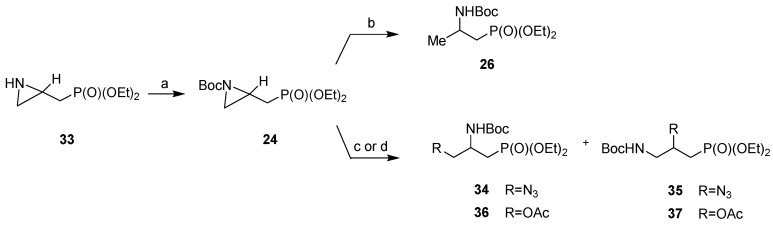
Synthesis of the racemic (aziridin-2-yl)methylphosphonate (*RS*)-**24**, aminopropylphosphonate (*RS*)-**26**, azidophosphonates (*RS*)-**34** and (*RS*)-**35** and acetoxyphosphonates (*RS*)-**36** and (*RS*)-**37**. Reagents and conditions: (**a**) Boc_2_O, EtOH, rt, 3 h, 99%; (**b**) H_2_, 20% Pd(OH)_2_-C, EtOH, 1.01 bar, 18 h, 77%; (**c**) TMSN_3_, rt, 4 d, 27% for (*RS*)-**34** and 3% for (*RS*)-**35**; (**d**) AcOH, rt, 24 h, 95% for a mixture of (*RS*)-**36** and (*RS*)-**37**.

**Scheme 5:**
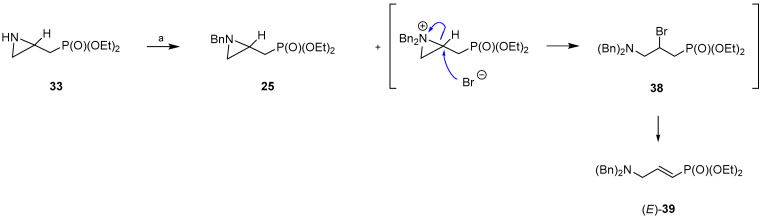
Synthesis of the racemic (aziridin-2-yl)methylphosphonate (*RS*)-**25**. Reagents and conditions: (**a**) BnBr, K_2_CO_3_, THF, rt, 24 h, 62%.

Preparation of both activated (R = Boc) and non-activated (R = Bn) aziridinephosphonates (*RS*)-**24** and (*RS*)-**25** was undertaken (Scheme 4 and Scheme 5) in order to compare the influence of electron-withdrawing and electron-donating groups on the regioselectivity of the nucleophilic ring-opening reaction in respective (aziridin-2-yl)methylphosphonates. When (aziridin-2-yl)methylphosphonate (*RS*)-**33** was subjected to the reaction with di-*tert*-butyl dicarbonate (Boc_2_O) at room temperature for 3 h corresponding *N*-[(*tert*-butoxycarbonyl)aziridin-2-yl]methylphosphonate (*RS*)-**24** was obtained in 99% yield (Scheme 4). The catalytic hydrogenation of (*RS*)-**24** led to the regiospecific formation of diethyl 2-(*tert*-butoxycarbonylamino)propylphosphonate (*RS*)-**26** in 77% yield. However, when aziridinephosphonate (*RS*)-**24** was treated with trimethylsilyl azide at room temperature formation of a 71:29 mixture of 2-amino-3-azidopropylphosphonate (*RS*)-**34** (δ^31^P = 27.44 ppm) and regioisomeric 3-amino-2-azidopropylphosphonate (*RS*)-**35** (δ^31^P = 26.47 ppm) was observed after 4 days. After purification of the crude reaction mixture on a silica gel column both regioisomers (*RS*)-**34** and (*RS*)-**35** were isolated in 27 and 3% yield, respectively. Similarly, treatment of aziridinephosphonate (*RS*)-**24** with glacial acid at room temperature for 24 h resulted in the formation of a 77:23 mixture of two regioisomeric products (*RS*)-**36** (δ^31^P = 27.56 ppm) and (*RS*)-**37** (δ^31^P = 26.25 ppm). Unfortunately, several attempts at chromatographic separation (silica gel column and HPLC) of the mixture of isomeric phosphonate appeared fruitless. The structures of both products have been established based on the analysis of the ^1^H NMR spectra taken for the fractions that were enriched for the respective regioisomers.

Our previous studies proved that non-activated aziridinephosphonates, namely benzylamino-[1-(1-phenylethyl)aziridin-2-yl]methylphosphonates [52] and hydroxy-[1-(1-phenylethyl)aziridin-2-yl]methylphosphonates [50,55], react regiospecifically with nucleophiles on less-hindered C3 atom to form aziridine ring-opening products. Following these observations, analogous *N*-benzylated azidirinephosphonate (*RS*)-**25** was selected as a substrate for the synthesis of designed compounds (*RS*)-**26**, (*RS*)-**27**, and (*RS*)-**28**. When (aziridin-2-yl)methylphosphonate (*RS*)-**33** was treated with benzyl bromide in the presence of potassium carbonate at room temperature for 24 h, aziridinephosphonate (*RS*)-**25** (δ^31^P = 28.55 ppm; 85%) was formed together with 3-(*N*,*N*-dibenzylamino)-2-bromopropylphosphonate as a by-product **38** (δ^31^P = 27.00 ppm; 15%) (Scheme 5). After chromatographic purification of crude reaction mixture *N*-benzyl-(aziridin-2-yl)methylphosphonate (*RS*)-**25** in 62% yield was obtained. However, instead of compound **38** (δ^31^P = 27.00 ppm), (*E*)-3-(*N*,*N*-dibenzylamino)prop-1-enylphosphonate (*E*)-**39** was isolated (δ^31^P = 18.25 ppm). Since crude aziridinephosphonate (*RS*)-**33** was used for the preparation of (*RS*)-**25**, it can be concluded that subsequent benzylation occurred leading to the formation of unstable aziridine ion, which upon reaction with bromine anion and further elimination of HBr on silica gel gave phosphonate (*E*)-**39** (Scheme 5). These observations are in good agreement with the reactivity of aziridinium ion derived from respectively functionalized phosphonates [56,57] and 2-(bromomethyl)aziridines [58].

**Scheme 6:**
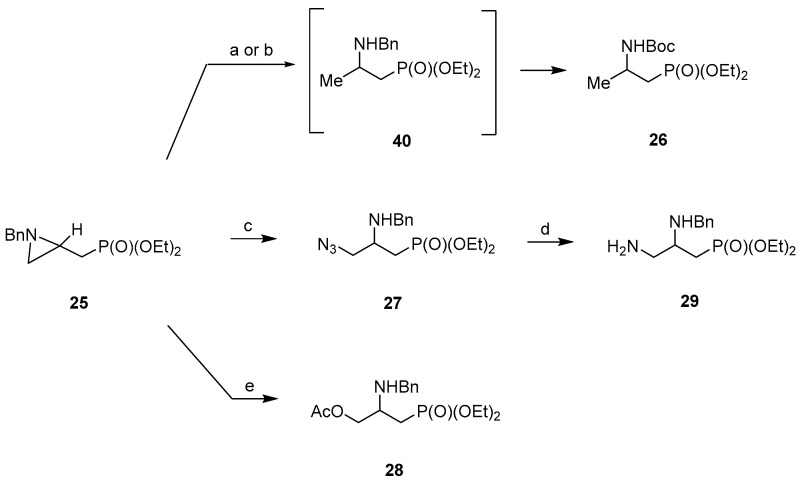
Transformations of the racemic (aziridin-2-yl)methylphosphonate (*RS*)-**25** into (*RS*)-**26**, (*RS*)-**29**, and (*RS*)-**28**. Reagents and conditions: (**a**) H_2_, 20% Pd(OH)_2_-C, Boc_2_O, EtOH, 1.01 bar, 10 d, 52%; (**b**) H_2_, 20% Pd(OH)_2_-C, Boc_2_O, EtOH, 10 bar, 4 d, 53%; (**c**) TMSN_3_, rt, 4 d, 84%; (**d**) PPh_3_, toluene, rt, 2 h, 88%; (**e**) AcOH, rt, 24 h, 77%.

The racemic diethyl *N*-benzyl-(aziridin-2-yl)methylphosphonate (*RS*)-**25** was employed as a starting material in the nucleophilic ring-opening reactions. During catalytic hydrogenation of *N*-Bn-aziridinephosphonate (*RS*)-**25** in the presence of Boc_2_O the regiospecific ring-opening at C3, removal of the *N*-benzyl group, and simultaneous protection of the amino function with Boc group were executed to form compound (*RS*)-**26** which was isolated in 52% yield after column chromatography and crystallization (Scheme 6). When the reaction was conducted under atmospheric pressure complete conversion of (*RS*)-**25** into 2-(*tert*-butoxycarbonylamino)propylphosphonate (*RS*)-**26** was achieved after 10 days. However, when the reaction was discontinued after 24 h, a 47:53 mixture of phosphonate (*RS*)-**26** (δ^31^P = 28.64 ppm) and 2-(benzylamino)propylphosphonate (*RS*)-**40** (δ^31^P = 28.97 ppm) was obtained. When hydrogenolysis was carried out at a pressure of 10 bar, reaction time was significantly shortened (10 days vs. 4 days) and the phosphonate (*RS*)-**26** was obtained in 53% yield after silica gel column chromatography and crystallization. The reactions of *N*-benzyl-(aziridin-2-yl)methylphosphonate (*RS*)-**25** with both trimethylsilyl azide and acetic acid proceeded also regiospecifically with the opening of aziridine ring at less hindered C3 atom (Scheme 6). In this way, 3-azido-2-benzylaminopropylphosphonate (*RS*)-**27** was obtained while treating (*RS*)-**25** with neat trimethylsilyl azide at room temperature for 4 days, and phosphonate (*RS*)-**27** was isolated in 84% yield after purification on a silica gel column. Then the subsequent reduction of azido function in (*RS*)-**27** using triphenylphosphine gave 2,3-diaminopropylphosphonate (*RS*)-**29** which was obtained in 88% after chromatographic purification (Scheme 6). On the other hand, treatment of *N*-benzyl-(aziridin-2-yl)methylphosphonate (*RS*)-**25** with acetic acid gave phosphonate (*RS*)-**28** as the only product.

**Scheme 7:**
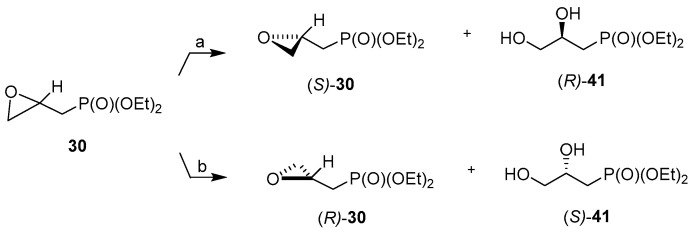
Hydrolytic kinetic resolution (HKR) of the racemic diethyl 2,3-epoxypropylphosphonate (*RS*)-**30**. Reagents and conditions: (**a**) H_2_O, rt, 4 d, (*R*,*R*)-salen-Co(II)-OAc, 45%; (**b**) H_2_O, rt, 4 d, (*S*,*S*)-salen-Co(II)-OAc, 44%.

The application of the above-mentioned protocols for the synthesis of enantiomeric phosphonates respectively functionalized at C2 and C3 required possessing enantiomeric epoxypropylphosphonate (*R*)- and (*S*)-**30**. The enantiomerically enriched (*S*)- and (*R*)-diethyl 2,3-epoxypropylphosphonates (*S*)-**30** (*ee* 96%) and (*R*)-**30** (*ee* 92%) were obtained via the hydrolytic kinetic resolution (HKR) of the racemic epoxide (*RS*)-**30** using Jacobsen’s complexes as respective chiral catalysts based on the methodology described in the literature (Scheme 7) [59].

**Scheme 8:**
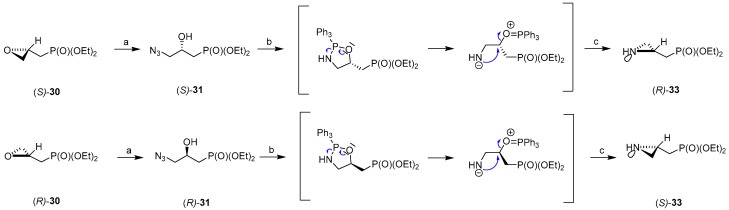
Syntheses of enantiomerically enriched (aziridin-2-yl)methylphosphonates (*R*)-**33** and (*S*)-**33**. Reagents and conditions: (**a**) NaN_3_, (NH_4_)_2_SO_4_, MeOH, reflux, 4 h, 84% for (*S*)-**31** and 92% for (*R*)-**31**; (**b**) PPh_3_, toluene, rt, 2 h; (**c**) reflux, 1 h, 52% for (*R*)-**31** and 47% for (*S*)-**33**.

The epoxides (*S*)-**30** and (*R*)-**30** were transformed into corresponding enantiomerically enriched diethyl (*S*)- and (*R*)-3-azido-2-hydroxypropylphosphonates (*S*)-**31** (*ee* 96%) and (*R*)-**31** (*ee* 96%) respectively, which were then subjected to the two-step reaction sequence leading to the formation of enantiomerically enriched diethyl (*R*)- and (*S*)-(aziridin-2-yl)methylphosphonates (*R*)-**33** and (*S*)-**33** (Scheme 8). As reported in the literature, during the transformation of unstable intermediate oxazaphospholidine into aziridinephosphonate **33** inversion of configuration at C2 occurs, thereby aziridinephosponate (*S*)-**33** was obtained from epoxide (*R*)-**30**, while (*R*)-**33** was produced from epoxide (*S*)-**30** [54,60].

**Scheme 9:**
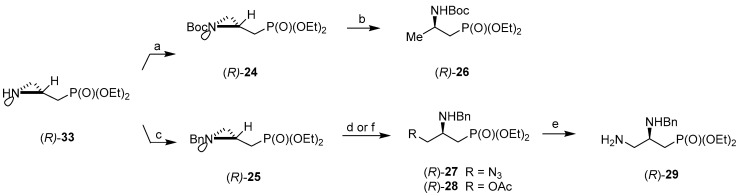
Syntheses of enantiomerically enriched aziridinephosphonates (*R*)-**24** and (*R*)-**25** and their transformations. Reagents and conditions: (**a**) Boc_2_O, EtOH, rt, 3 h, 99%; (**b**) H_2_, 20% Pd(OH)_2_-C, EtOH, 1.01 bar, 18 h, 80%; (**c**) BnBr, K_2_CO_3_, THF, rt, 24 h, 68%; (**d**) TMSN_3_, rt, 4 d, 85% for (*R*)-**27**; (**e**) PPh_3_, toluene, rt, 2 h, 78% from (*R*)-**27**; (**f**) AcOH, rt, 24 h, 82% for (*R*)-**28**.

**Scheme 10:**
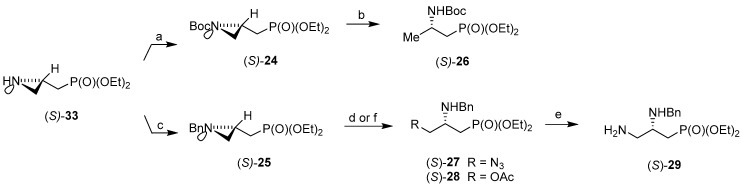
Syntheses of enantiomerically enriched aziridinephosphonates (*S*)-**24** and (*S*)-**25** and their transformations. Reagents and conditions: (**a**) Boc_2_O, EtOH, rt, 3 h, 90%; (**b**) H_2_, 20% Pd(OH)_2_-C, EtOH, 1.01 bar, 18 h, 78%; (**c**) BnBr, K_2_CO_3_, THF, rt, 24 h, 58%; (**d**) TMSN_3_, rt, 4 d, 83% for (*S*)-**27**; (**e**) PPh_3_, toluene, rt, 2 h, 75% from (*S*)-**27**; (**f**) AcOH, rt, 24 h, 83% for (*S*)-**28**.

Aziridinephosphonates (*R*)-**33** and (*S*)-**33** were then transformed into their *N*-protected derivatives (*R*)-**24** and (*S*)-**24** (*N*-Boc) or (*R*)-**25** and (*S*)-**25** (*N*-Bn), to make them suitable for further regiospecific ring-opening reactions with respective nucleophiles (Scheme 9 and Scheme 10).

2-(*N*-Boc-amino)propylphosphonates (*R*)-**26** and (*S*)-**26** were obtained directly via hydrogenolysis of respective *N*-Boc-aziridinephosphonates (*R*)-**24** and (*S*)-**24** (Scheme 9 and Scheme 10). On the other hand, *N*-Bn-aziridinephosphonate (*R*)-**25** was subjected to a regiospecific ring-opening reaction with neat trimethylsilyl azide to form 3-azidophosphonate (*R*)-**27** (*ee* 98%) and subsequently reduced to 2,3-diaminophosphonate (*R*)-**29** with triphenylphosphine, whereas treatment of (*R*)-**26** with glacial acetic acid gave compound (*R*)-**28** (*ee* 97%) (Scheme 9). In an analogous manner, enantiomerically enriched *N*-Bn-aziridinephosphonate (*S*)-**25** was used for the preparation of (*S*)-**27** (*ee* 94%), (*S*)-**28** (*ee* 97%) and (*S*)-**29** (Scheme 10).

**Scheme 11:**
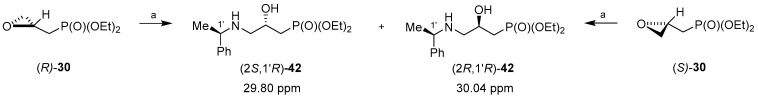
Epoxide ring-opening reaction in enantiomerically enriched epoxides (*S*)-**30** and (*R*)-**30** with (*R*)-1-phenylethylamine. Reagents and conditions: (**a**) Ca(OTf)_2_, (*R*)-1-phenylethylamine, 50 °C, 1 h.

To establish enantiomeric purity of the 2,3-epoxypropylphosphonates (*S*)-**30** and (*R*)-**30**, the direct regiospecific ring-opening reaction of (*S*)-**30** and (*R*)-**30** with enantiomerically pure (*R*)-1-phenylethylamine in the presence of the catalytic amount of Ca(OTf)_2_ [61] was employed (Scheme 11) as a simple and convenient alternative to the two-step procedure described in the literature, i.e., the epoxide ring-opening with dibenzylamine followed by esterification of the resulting aminoalcohol with (*S*)-*O*-methylmandelic acid [59]. The ^31^P NMR spectrum taken for the crude product obtained after completion of the reaction of epoxide (*R*)-**30** with (*R*)-1-phenylethylamine revealed the formation of two products (2*R*,1*R*′)-**42** (δ^31^P = 30.04 ppm) and (2*S*,1*R*′)-**42** (δ^31^P = 29.80 ppm) in a 96:4 ratio, and the enantiomeric excess of epoxide (*R*)-**30** was established as 92%. Analogously, as a 98:2 mixture of chiral aminoalcohols (2*S*,1*R*′)-**42** (δ^31^P = 29.80 ppm) and (2*R*,1*R*′)-**42** (δ^31^P = 30.04 ppm) were obtained from epoxide (*S*)-**30** and (*R*)-1-phenylethylamine, the enantiomeric purity of starting epoxide (*S*)-**30** was determined as 96% (Scheme 11).

Enantiomeric purities of 3-azido-2-hydroxypropylphosphonates (*R*)-**31** (*ee* 96%) and (*S*)-**31** (*ee* 96%) were established based on their enantiodifferentiation in ^31^P NMR using quinine (4 equiv) as discriminating agent [53]. Separation on HPLC with an application of a chiral column was useful for the determination of enantiomeric purity of 3-azido-2-benzylaminopropylphosphonates (*R*)-**27** (*ee* 98%) and (*S*)-**27** (*ee* 94%) as well as 3-acetoxy-2-benzylaminopropylphosphonates, namely *ee* 97% for both (*R*)-**28** and (*S*)-**28** (Supplementary Materials).

## 3. Materials and Methods

### 3.1. General Information

The ^1^H, ^13^C, and ^31^P NMR spectra were taken in CDCl_3_ on the Bruker Avance III spectrometers (600 MHz) with TMS as the internal standard at 600, 151, and 243 MHz, respectively. Coupling constants *J* are given in Hz. The IR spectra were measured on an Infinity MI-60 FT-IR spectrometer. Melting points were determined on a Boetius apparatus and were uncorrected. Elemental analyses were performed by the Microanalytical Laboratory of this faculty on Perkin–Elmer PE 2400 CHNS analyzer, and their results were found to be in good agreement (±0.3%) with the calculated values. Polarimetric measurements were conducted with an Optical Activity PolAAr 3001 apparatus. The HPLC separations were performed using a Waters HPLC system consisting of binary HPLC pump (Waters 2545), a diode array detector (Waters 2998) and an Chiralpack column AD, 0.46 × 25 cm with a particle size of 10 µm. The following adsorbents were used: column chromatography, Merck silica gel 60 (70–230 mesh), analytical TLC, and Merck TLC plastic sheets silica gel 60 F254. The TLC plates were developed in chloroform-methanol, dichloromethane-methanol and hexane-isopropanol solvent systems. Visualization of spots was achieved with iodine vapors. All solvents were purified by methods described in the literature. 2,3-Epoxypropylphosphonates **30** [59,62], 3-azido-2-hydroxypropylphosphonates **31** [53] and (aziridin-2-yl)methylphosphonates **33** [54] were obtained according to procedures described in the literature.

The ^1^H-, ^13^C- and ^31^P-NMR spectra of all newly synthesized compounds are provided in Supplementary Materials.

### 3.2. General Procedure for the Synthesis of Diethyl N-[(tert-Butoxycarbonyl)aziridin-2-yl]methylphosphonates (RS)-**24**, (R)-**24** and (S)-**24**

Crude (aziridin-2-yl)methylphosphonate (*RS*)-**33**, or (*R*)-**33** or (*S*)-**33** (1.00 mmol) and Boc_2_O (1.10 mmol) in anhydrous ethanol (3.6 mL) were stirred at room temperature for 3 h. The crude reaction mixtures were chromatographed on a silica gel column with chloroform-methanol (100:1, 50:1, *v*/*v*) to give pure (*RS*)-**24**, or (*R*)-**24** or (*S*)-**24**.

Diethyl (*RS*)-*N*-[*(tert*-butoxycarbonyl)aziridin-2-yl]methylphosphonate [(*RS*)-**24**]. According to the general procedure from (*RS*)-**33** (0.244 g, 1.26 mmol) and Boc_2_O (0.304 g, 1.39 mmol), (*RS*)-**24** (0.368 g, 99%) was obtained as a colorless oil. IR (film): ν = 2981, 2933, 1720, 1232, 1053 cm^−1^. ^1^H NMR (600 MHz, CDCl_3_): δ = 4.18–4.08 (m, 4H, CH_3_C*H*_2_OP), 2.63 (ddd, *J* = 14.4 Hz, *J* = 8.5 Hz, *J* = 3.9 Hz, 1H, *H*CCP), 2.37–2.31 (m, 1H, *H*_a_CH_b_P), 2.35 (d, *J* = 5.9 Hz, *H*_a_CH_b_CCP), 2.04 (d, *J* = 3.6 Hz, 1H, H_a_C*H*_b_CCP), 1.63 (ddd, *J* = 19.2 Hz, *J* = 15.2 Hz, *J* = 8.3 Hz, 1H, H_a_C*H*_b_P), 1.45 (s, 9H, (C*H*_3_)_3_C), 1.33 (t, *J* = 7.1 Hz, 6H, 2 × C*H*_3_CH_2_OP). ^13^C NMR (151 MHz, CDCl_3_): δ = 161.9 (C=O), 81.5, 61.9 (d, *J* = 6.4 Hz, C*C*OP), 32.0 (d, *J* = 1.6 Hz, *C*CP), 32.0 (d, *J* = 5.6 Hz, *C*CCP), 29.6 (d, *J* = 140.0 Hz, *C*P), 27.9, 16.5 (d, *J* = 6.3 Hz, *C*COP). ^31^P NMR (243 MHz, CDCl_3_): δ = 26.78. Anal. Calcd. for C_12_H_24_NO_5_P × 0.25 H_2_O: C, 48.40; H, 8.29; N, 4.71. Found: C, 48.26; H, 8.46; N, 4.59.

Diethyl (*R*)-*N*-[(*tert*-butoxycarbonyl)aziridin-2-yl]methylphosphonate [(*R*)-**24**]. According to the general procedure from (*R*)-**33** (0.077 g, 0.399 mmol) and Boc_2_O (0.096 g, 0.44 mmol), (*R*)-**24** (0.116 g, 99%) was obtained as a colorless oil. ${\left[\alpha \right]}_{D}^{20}$ = +57.3 (*c* 1.19, CH_2_Cl_2_). Anal. Calcd. for C_12_H_24_NO_5_P × 0.25 H_2_O: C, 48.40; H, 8.29; N, 4.71. Found: C, 48.11; H, 8.44; N, 4.95.

Diethyl (*S*)-*N*-[(*tert*-butoxycarbonyl)aziridin-2-yl]methylphosphonate [(*S*)-**24**]. According to the general procedure from (*S*)-**33** (0.063 g, 0.33 mmol) and Boc_2_O (0.078 g, 0.36 mmol), (*S*)-**24** (0.086 g, 90%) was obtained as a colorless oil. ${\left[\alpha \right]}_{D}^{20}$ = −52.6 (*c* 1.11, CH_2_Cl_2_). Anal. Calcd. for C_12_H_24_NO_5_P × 0.25 H_2_O: C, 48.40; H, 8.29; N, 4.71. Found: C, 48.26; H, 8.56; N, 4.90.

### 3.3. General Procedure for Benzylation of (RS)-**33**, (R)-**33** and (S)-**33**

Crude (aziridin-2-yl)methylphosphonate (*RS*)-**33**, or (*R*)-**33** or (*S*)-**33** (1.00 mmol), benzyl bromide (1.00 mmol) and potassium carbonate (2.50 mmol) in anhydrous THF (9.0 mL) were stirred at room temperature for 24 h. The crude reaction mixtures were chromatographed on a silica gel column with dichloromethane-methanol (100:1, 50:1, *v*/*v*) to give pure (*RS*)-**25**, or (*R*)-**25** or (*S*)-**25**. In addition to the expected aziridinephosphonate **25** traces of alkene **39** were also isolated.

Diethyl (*RS*)-*N*-(benzylaziridin-2-yl)methylphosphonate [(*RS*)-**25**]. According to the general procedure from racemic (*RS*)-**33** (0.254 g, 1.31 mmol), benzyl bromide (0.225 g, 1.31 mmol) and potassium carbonate (0.454 g, 3.29 mmol), (*RS*)-**25** (0.231 g, 62%) was obtained as a colorless oil. IR (film): ν = 3029, 2984, 2831, 1251, 1028, 735 cm^−1^. ^1^H NMR (600 MHz, CDCl_3_): δ = 7.34–7.24 (m, 5H, Ar-*H*), 4.14–4.04 (m, 4H, 2 × CH_3_C*H*_2_OP), 3.61 (d, *J* = 13.3 Hz, 1H, *H*_a_CH_b_Ph), 3.30 (d, *J* = 13.3 Hz, 1H, H_a_C*H*_b_Ph), 2.14 (ddd, *J* = 17.9 Hz, *J* = 15.1 Hz, *J* = 5.2 Hz, 1H, *H*_a_CH_b_P), 1.82–1.70 (m, 3H, *H*CCP, H_a_C*H*_b_P, *H*_a_CH_b_CCP), 1.52 (d, *J* = 6.2 Hz, H_a_C*H*_b_CCP), 1.31 (t, *J* = 7.1 Hz, 6H, 2 × C*H*_3_CH_2_OP). ^13^C NMR (151 MHz, CDCl_3_): δ = 138.8, 128.4, 128.1, 127.2, 64.4, 61.7 (d, *J* = 6.4 Hz, C*C*OP), 61.6 (d, *J* = 6.5 Hz, C*C*OP), 33.9 (d, *J* = 7.1 Hz, *C*CCP), 33.4 (d, *J* = 3.0 Hz, *C*CP), 30.0 (d, *J* = 138.4 Hz, *C*P), 16.46 (d, *J* = 6.0 Hz, *C*COP). ^31^P NMR (243 MHz, CDCl_3_): δ = 28.55. Anal. Calcd. for C_14_H_22_NO_3_P × 0.75 H_2_O: C, 56.66; H, 7.98; N, 4.72. Found: C, 56.56; H, 7.90; N, 4.82.

Diethyl (*R*)-*N*-(benzylaziridin-2-yl)methylphosphonate [(*R*)-**25**]. According to the general procedure from (*R*)-**33** (0.192 g, 0.99 mmol), benzyl bromide (0.170 g, 0.99 mmol) and potassium carbonate (0.344 g, 2.49 mmol), (*R*)-**25** (0.192 g, 68%) was obtained as a colorless oil. ${\left[\alpha \right]}_{D}^{20}$ = +28.6 (*c* 1.53, CH_2_Cl_2_). Anal. Calcd. for C_14_H_22_NO_3_P × 0.5 H_2_O: C, 57.53; H, 7.93; N, 4.79. Found: C, 57.61; H, 7.97; N, 4.84.

Diethyl (*S*)-*N*-(benzylaziridin-2-yl)methylphosphonate [(*S*)-**25**]. According to the general procedure from (*S*)-**33** (0.158 g, 0.82 mmol), benzyl bromide (0.140 g, 0.82 mmol) and potassium carbonate (0.283 g, 2.05 mmol), (*S*)-**25** (0.135 g, 58%) was obtained as a colorless oil. ${\left[\alpha \right]}_{D}^{20}$ = −28.1 (*c* 1.01, CH_2_Cl_2_). Anal. Calcd. for C_14_H_22_NO_3_P × 0.5 H_2_O: C, 57.53; H, 7.93; N, 4.79. Found: C, 57.38; H, 7.94; N, 4.95.

Diethyl (*E*)-3-(*N*,*N*-dibenzylamino)-prop-1-en-1-ylphosphonate [(*E*)-**39**]. According to the general procedure from racemic (*RS*)-**33** (0.254 g, 1.31 mmol), benzyl bromide (0.225 g, 1.31 mmol) and potassium carbonate (0.454 g, 3.29 mmol), (*E*)-**39** (0.059g, 12%) was obtained as a yellow oil. IR (film): ν = 3085, 3061, 3028, 2982, 2714, 1633, 1602 cm^−1^. ^1^H NMR (600 MHz, CDCl_3_): δ = 7.36 (d, *J* = 7.3 Hz, 4H, Ar-*H*), 7.31 (t, *J* = 7.3 Hz, 4H, Ar-*H*), 7.24 (d, *J* = 7.3 Hz, 2H, Ar-*H*), 6.82 (ddt, *J* = 22.5 Hz, *J* = 17.2 Hz, *J* = 5.4 Hz, 1H, *H*CCP), 6.04–5.94 (m, 1H, *H*CP), 4.10–4.01 (m, 4H, 2 × CH_3_C*H*_2_OP), 3.59 (s, 4H, C*H*_2_Ph), 3.25–3.22 (m, C*H*_2_CCP), 1.31 (t, *J* = 7.1 Hz, 6H, 2 × C*H*_3_CH_2_OP). ^13^C NMR (151 MHz, CDCl_3_): δ = 150.8 (d, *J* = 5.0 Hz, *C*CP), 138.9, 128.7, 128.3, 127.1, 118.7 (d, *J* = 187.5 Hz, *C*P), 61.7 (d, *J* = 5.5 Hz, C*C*OP), 58.2, 55.8 (d, *J* = 22.5 Hz, *C*CCP), 16.4 (d, *J* = 6.3 Hz, *C*COP). ^31^P NMR (243 MHz, CDCl_3_): δ = 18.25. Anal. Calcd. for C_21_H_28_NO_3_P × 0.25 H_2_O: C, 66.74; H, 7.60; N, 3.71. Found: C, 66.51; H, 7.54; N, 3.79.

### 3.4. General Procedures for the Synthesis of (RS)-**26**, (R)-**26** and (S)-**26**

Method A: A solution of **24** (1.00 mmol) in ethanol (5 mL) was stirred under an atmospheric pressure of hydrogen over 20% Pd/C (25 mg) at room temperature for 18 h. The suspension was filtered through a layer of Celite, then the solution was concentrated and crystallized from hexane to produce corresponding **26**.

Method B: A solution of **25** (1.00 mmol) in ethanol (5 mL) containing Boc_2_O (1.10 mmol) was stirred under an atmospheric pressure of hydrogen over 20% Pd/C (25 mg) at room temperature for 10 days. The suspension was filtered through a layer of Celite, then the solution was concentrated and chromatographed on a silica gel column with hexane-isopropanol (100:1, 50:1, 30:1, *v*/*v*). The appropriate fractions were collected and crystallized from hexane to produce **26**.

Method C: A solution of **25** (1.00 mmol) in ethanol (5 mL) containing Boc_2_O (1.10 mmol) was stirred in a pressure reactor (10 bar) over 20% Pd(OH)2/C (25 mg) at room temperature for 4 days. The suspension was filtered through a layer of Celite, then the solution was concentrated and chromatographed on a silica gel column with hexane-isopropanol (100:1, 50:1, 30:1, *v*/*v*). The appropriate fractions were collected and crystallized from hexane to produce **26**.

Diethyl (*RS*)-2-(*tert*-butoxycarbonylamino)propylphosphonate [(*RS*)-**26**]. According to the general procedure (method A) from (*RS*)-**24** (0.148 g, 0.50 mmol) (*RS*)-**26** (0.114 g, 77%) was obtained as a white solid. M.p. 58–61 °C. IR (film): ν = 3418, 3050, 2981, 1698, 1542, 1172 cm^−1^. ^1^H NMR (600 MHz, CDCl_3_): δ = 5.07 (bs, 1H, *H*NCHCP), 4.16–4.05 (m, 4H, 2 × CH_3_C*H*_2_OP), 4.05–3.92 (m, 1H, *H*CCP), 2.06 (ddd, *J* = 18.7 Hz, *J* = 15.4 Hz, *J* = 5.6 Hz, 1H, *H_a_*CH_b_P), 1.98 (td, *J* = 16.8 Hz, *J* = 6.2 Hz, H_a_C*H*_b_P), 1.43 (s, 9H, (C*H*_3_)_3_C), 1.36 (t, *J* = 7.1 Hz, 3H, C*H*_3_CH_2_OP), 1.35 (t, *J* = 7.1 Hz, 3H, C*H*_3_CH_2_OP), 1.31 (d, *J* = 6.7 Hz, 3H, C*H*_3_CH). ^13^C NMR (151 MHz, CDCl_3_): δ = 155.0 (C=O), 79.2, 61.7 (d, *J* = 6.4 Hz, C*C*OP), 61.6 (d, *J* = 6.6 Hz, C*C*OP), 42.5, 32.4 (d, *J* = 133.4 Hz, *C*P), 28.4 (3 × *C*H_3_), 21.7, 16.4 (d, *J* = 6.1 Hz, *C*COP). ^31^P NMR (243 MHz, CDCl_3_): δ = 28.64. Anal. Calcd. for C_12_H_26_NO_5_P × 0.5 H_2_O: C, 48.08; H, 8.91; N, 4.67. Found: C, 48.33; H, 9.04; N, 4.77.

Diethyl (*RS*)-2-(*tert*-butoxycarbonylamino)propylphosphonate [(*RS*)-**26**]. According to the general procedure (method B) from (*RS*)-**25** (0.064 g, 0.23 mmol) and Boc_2_O (0.054 g, 0.25 mmol) (*RS*)-**26** (0.035 g, 52%) was obtained as a white solid.

Diethyl (*RS*)-2-(*tert*-butoxycarbonylamino)propylphosphonate [(*RS*)-**26**]. According to the general procedure (method C) from (*RS*)-**25** (0.063 g, 0.22 mmol) and Boc_2_O (0.053 g, 0.24 mmol) (*RS*)-**26** (0.035 g, 53%) was obtained as a white solid.

Diethyl (*R*)-2-(*tert*-butoxycarbonylamino)propylphosphonate [(*R*)-**26**]. According to the general procedure (method A) from (*R*)-**24** (0.041 g, 0.140 mmol) (*R*)-**26** (0.033 g, 80%) was obtained as a white solid. M.p. 71–73 °C. ${\left[\alpha \right]}_{D}^{20}$ = +10.90 (*c* 1.10, CH_2_Cl_2_). Anal. Calcd. for C_12_H_26_NO_5_P × 0.75 H_2_O: C, 46.68; H, 8.98; N, 4.54. Found: C, 46.67; H, 8.68; N, 4.42.

Diethyl (*S*)-2-(*tert*-butoxycarbonylamino)propylphosphonate [(*S*)-**26**]. According to the general procedure (method A) from (*S*)-**24** (0.041 g, 0.14 mmol) (*S*)-**26** (0.032 g, 78%) was obtained as a white solid. M.p. 67–69 °C. ${\left[\alpha \right]}_{D}^{20}$ = −10.90 (*c* 1.04, CH_2_Cl_2_). Anal. Calcd. for C_12_H_26_NO_5_P × 0.25 H_2_O: C, 48.08; H, 8.91; N, 4.67. Found: C, 48.37; H, 9.14; N, 4.62.

### 3.5. Ring-Opening Reaction of Diethyl N-[(tert-Butoxycarbonyl)aziridin-2-yl]methylphosphonate (RS)-**24** with Trimethylsilyl Azide

A mixture of (*RS*)-**24** (0.134 g, 0.46 mmol) and neat TMSN_3_ (0.158 g, 1.37 mmol) was stirred at room temperature for 4 days. The crude product was chromatographed on a silica gel column with chloroform-hexane (1:1, *v*/*v*) to give pure 3-azidopropylphosphonate (*RS*)-**34** (0.042 g, 27%) and 2-azidopropylphosphonate (*RS*)-**35** (0.005 g, 3%).

Diethyl (*RS*)-3-azido-2-(*tert*-butoxycarbonylamino)propylphosphonate [(*RS*)-**34**]. Colorless oil. IR (film): ν = 3284, 2981, 2871, 2102, 1710, 1528, 1168 cm^−1^. ^1^H NMR (600 MHz, CDCl_3_): δ = 5.23 (bs, 1H, *H*NCHP), 4.17–4.07 (m, 4H, 2 × CH_3_C*H*_2_OP), 4.07–3.98 (m, 1H, C*H*CP), 3.60 (dd, *J* = 12.2 Hz, *J* = 4.8 Hz, *H*_a_CH_b_CCP), 3.50 (dd, *J* = 12.2 Hz, *J* = 5.7 Hz, H_a_C*H_b_*CCP), 2.13–2.07 (m, 1H, *H*_a_CH_b_P), 2.02 (ddd, *J* = 18.1 Hz, *J* = 15.4 Hz, *J* = 6.4 Hz, 1H, H_a_C*H*_b_P), 1.43 (s, 9H, (C*H*_3_)_3_C), 1.34 and 1.33 (2 × t, *J* = 7.0 Hz, 6H, 2 × C*H*_3_CH_2_OP). ^13^C NMR (151 MHz, CDCl_3_): δ = 154.9 (C=O), 79.9, 62.1 (d, *J* = 6.4 Hz, C*C*OP), 61.9 (d, *J* = 6.6 Hz, C*C*OP), 54.1 (d, *J* = 8.3 Hz, *C*CCP), 46.3, 28.3, 27.9 (d, *J* = 138.3 Hz, *C*P), 16.4 (d, *J* = 5.8 Hz, *C*COP). ^31^P NMR (243 MHz, CDCl_3_): δ = 27.44. Anal. Calcd. for C_12_H_25_N_4_O_5_P: C, 42.85; H, 7.49; N, 16.66. Found: C, 42.80; H, 7.78; N, 16.42.

Diethyl (*RS*)-2-azido-3-(*tert*-butoxycarbonylamino)propylphosphonate [(*RS*)-**35**]. Colorless oil. IR (film): ν = 3317, 2978, 2926, 2113, 1707, 1522, 1165 cm^−1^. ^1^H NMR (600 MHz, CDCl_3_): δ = 4.95 (bs, 1H, *H*NCHP), 4.19–4.09 (m, 4H, 2 × CH_3_C*H*_2_OP), 3.90–3.81 (m, 1H, C*H*CP), 3.42 (ddd, *J* = 14.1 Hz, *J* = 6.2 Hz, *J* = 4.8 Hz, *H*_a_CH_b_CCP), 3.25–3.18 (m, 1H, H_a_C*H_b_*CCP), 2.01 (dd, *J* = 18.6 Hz, *J* = 6.6 Hz, 2H, *H*_a_CH_b_P and H_a_C*H*_b_P), 1.44 (s, 9H, (C*H*_3_)_3_C), 1.35 (t, *J* = 7.1 Hz, 6H, 2 × C*H*_3_CH_2_OP). ^13^C NMR (151 MHz, CDCl_3_): δ = 155.8 (C=O), 79.9, 62.1 (d, *J* = 6.6 Hz, C*C*OP), 57.6, 45.0 (d, *J* = 13.3 Hz, *C*CCP), 28.9 (d, *J* = 142.9 Hz, *C*P), 28.3, 16.4 (d, *J* = 6.0 Hz, *C*COP), 16.4 (d, *J* = 6.0 Hz, *C*COP). ^31^P NMR (243 MHz, CDCl_3_): δ = 26.47. Anal. Calcd. for C_12_H_25_N_4_O_5_P: C, 42.85; H, 7.49; N, 16.66. Found: C, 42.75; H, 7.68; N, 16.39.

### 3.6. Ring-Opening of Diethyl N-[(tert-Butoxycarbonyl)aziridin-2-yl]methylphosphonate (RS)-**24** with Acetic Acid

A mixture of (*RS*)-**24** (0.093 g, 0.32 mmol) and glacial AcOH (0.636 mL, 11.11 mmol) was stirred at room temperature for 24 h. The reaction mixture was then concentrated in vacuo with toluene (3 × 10 mL). The crude product was chromatographed on a silica gel column with dichloromethane-methanol (200:1, 50:1 *v*/*v*) to give an inseparable mixture of 3-acetoxypropylphosphonate (*RS*)-**36** and 2-acetoxypropylphosphonate (*RS*)-**37** (0.106 g, 95%). ^31^P NMR (243 MHz, CDCl_3_): δ = 27.56 ppm (major regioisomer) and 26.25 ppm (minor regioisomer).

### 3.7. General Procedure for the Ring-Opening Reaction of Diethyl N-(Benzylaziridin-2-yl]methylphosphonates (RS)-**25**, (R)-**25** and (S)-**25** with Trimethylsilyl Azide

A mixture of (*RS*)-**25**, or (*R*)-**25**, or (*S*)-**25** (1.00 mmol) and neat TMSN_3_ (3.00 mmol) was stirred at room temperature for 4 days. The crude product was chromatographed on a silica gel column with dichloromethane-methanol (200:1, *v*/*v*) to give pure (*RS*)-**27**, or (*R*)-**27**, or (*S*)-**27**.

Diethyl (*RS*)-3-azido-2-(benzylamino)propylphosphonate [(*RS*)-**27**]. According to the general procedure from (*RS*)-**25** (0.106 g, 0.37 mmol) and TMSN_3_ (0.129 g, 1.12 mmol) azidophosphonate (*RS*)-**27** (0.103g, 84%) was obtained as a colorless oil. IR (film): ν = 3454, 3028, 2983, 2868, 2103, 1453, 1236, 1026, 737, 699 cm^−1^. ^1^H NMR (600 MHz, CDCl_3_): δ = 7.35–7.30 (m, 4H, Ar-*H*), 7.25–7.23 (m, 1H, Ar-*H*), 4.13–4.03 (m, 4H, 2 × CH_3_C*H*_2_OP), 3.83 (d, *J* = 13.1 Hz, 1H, *H*_a_CH_b_Ph), 3.80 (d, *J* = 13.1 Hz, 1H, H_a_C*H*_b_Ph), 3.53 (dd, *J* = 12.3 Hz, *J* = 4.8 Hz, *H*_a_CH_b_CCP), 3.40 (ddd, *J* = 12.3 Hz, *J* = 4.9 Hz, *J* = 0.5 Hz, 1H, H_a_C*H*_b_CCP), 3.16–3.10 (m, 1H, *H*CCP), 2.00 (ddd, *J* = 18.2 Hz, *J* = 15.6 Hz, *J* = 6.7 Hz, 1H, *H_a_*CH_b_P), 1.96 (ddd, *J* = 18.5 Hz, *J* = 15.6 Hz, *J* = 6.2 Hz, 1H, H_a_C*H*_b_P), 1.30 and 1.29 (2 × t, *J* = 7.0 Hz, 6H, 2 × C*H*_3_CH_2_OP). ^13^C NMR (151 MHz, CDCl_3_): δ = 139.6, 128.5, 128.2, 127.2, 61.8 (d, *J* = 6.1 Hz, C*C*OP), 61.8 (d, *J* = 6.5 Hz, C*C*OP), 54.4 (d, *J* = 10.3 Hz, *C*CCP), 52.4 (d, *J* = 2.2 Hz, *C*CP), 51.2, 28.8 (d, *J* = 139.7 Hz, *C*P), 16.4 (d, *J* = 5.9 Hz, *C*COP). ^31^P NMR (243 MHz, CDCl_3_): δ = 28.94. Anal. Calcd. for C_14_H_23_N_4_O_3_P × 0.25 H_2_O: C, 50.83; H, 7.16; N, 16.94. Found: C, 50.92; H, 7.23; N, 16.96.

Diethyl (*R*)-3-azido-2-(benzylamino)propylphosphonate [(*R*)-**27**]. According to the general procedure from (*R*)-**25** (0.130 g, 0.46 mmol) and TMSN_3_ (0.159 g, 1.38 mmol) azidophosphonate (*R*)-**27** (0.128 g, 85%) was obtained as a colorless oil.$\;{\left[\alpha \right]}_{D}^{20}$ = −6.50 (*c* 1.20, CH_2_Cl_2_). Anal. Calcd. for C_14_H_23_N_4_O_3_P × 0.25 H_2_O: C, 50.83; H, 7.16; N, 16.94. Found: C, 51.05; H, 7.34; N, 16.66.

Diethyl (*S*)-3-azido-2-(benzylamino)propylphosphonate [(*S*)-**27**]. According to the general procedure from (*S*)-**25** (0.073 g, 0.26 mmol) and TMSN_3_ (0.089 g, 0.77 mmol) azidophosphonate (*S*)-**27** (0.070g, 83%) was obtained as a colorless oil.$\;{\left[\alpha \right]}_{D}^{20}$ = +7.55 (*c* 1.06, CH_2_Cl_2_). Anal. Calcd. for C_14_H_23_N_4_O_3_P × 0.25 H_2_O: C, 50.83; H, 7.16; N, 16.94. Found: C, 50.99; H, 7.21; N, 16.69.

### 3.8. General Procedure for the Synthesis of Diethyl 2,3-Diaminopropylphosphonates (RS)-**29**, (R)-**29** and (S)-**29**

A mixture of (*RS*)-**27**, or (*R*)-**27**, or (*S*)-**27** (1.00 mmol) and triphenylphosphine (1.00 mmol) in toluene (6 mL) was stirred at room temperature for 2 h. The reaction mixture was concentrated in vacuo and then the crude product was chromatographed on a silica gel column with dichloromethane-methanol (50:1, 30:1, 15:1, 10:1, *v*/*v*) to give pure 2,3-diaminopropylphosphonates (*RS*)-**29**, or (*R*)-**29**, or (*S*)-**29**.

Diethyl (*RS*)-3-amino-2-(benzylamino)propylphosphonate [(*RS*)-**29**]. According to the general procedure from (*RS*)-**27** (0.081 g, 0.248 mmol) and PPh_3_ (0.065 g, 0.248 mmol) 2,3-diaminopropylphosphonate (*RS*)-**29** (0.065 g, 88%) was obtained as a yellowish oil. IR (film): ν = 3442, 3287, 3061, 3028, 2984, 2968, 1639, 1619, 1225, 1026, 700. ^1^H NMR (600 MHz, CDCl_3_): δ = 7.35–7.30 (m, 4H, Ar-*H*), 7.25–7.22 (m, 1H, Ar-*H*), 4.13–4.03 (m, 4H, 2 × CH_3_C*H*_2_OP), 3.81 (d, *J* = 13.1 Hz, 1H, *H*_a_CH_b_Ph), 3.77 (d, *J* = 13.1 Hz, 1H, H_a_C*H*_b_Ph), 3.02–2.94 (m, 2H, *H*_a_CH_b_CCP, *H*CCP), 2.71 (dd, *J* = 13.0 Hz, *J* = 5.6 Hz, 1H, H_a_C*H*_b_CCP), 2.02 (ddd, *J* = 18.2 Hz, *J* = 15.4 Hz, *J* = 6.8 Hz, 1H, *H_a_*CH_b_P), 1.92 (ddd, *J* = 18.5 Hz, *J* = 15.4 Hz, *J* = 5.8 Hz, 1H, H_a_C*H*_b_P), 1.30 (t, *J* = 7.1 Hz, 6H, C*H*_3_CH_2_OP), 1.29 (t, *J* = 7.0 Hz, 6H, C*H*_3_CH_2_OP). ^13^C NMR (151 MHz, CDCl_3_): δ = 140.2, 128.4, 128.2, 127.0, 61.7 (d, *J* = 6.6 Hz, C*C*OP), 61.7 (d, *J* = 6.6 Hz, C*C*OP), 53.9 (d, *J* = 2.6 Hz, *C*CP), 51.0, 44.9 (d, *J* = 10.2 Hz, *C*CCP), 28.6 (d, *J* = 139.7 Hz, *C*P), 16.5 (d, *J* = 5.9 Hz, *C*COP). ^31^P NMR (243 MHz, CDCl_3_): δ = 30.33. Anal. Calcd. for C_14_H_25_N_2_O_3_P × H_2_O: C, 52.82; H, 8.55; N, 8.80. Found: C, 52.67; H, 8.44; N, 8.58.

Diethyl (*R*)-3-amino-2-(benzylamino)propylphosphonate [(*R*)-**29**]. According to the general procedure from (*R*)-**27** (0.050 g, 0.15 mmol) and PPh_3_ (0.040 g, 0.15 mmol) (*R*)-2,3-diaminopropylphosphonate (*R*)-**29** (0.036 g, 78%) was obtained as a yellowish oil. ${\left[\alpha \right]}_{D}^{20}$ = +4.65 (*c* 0.93, CH_2_Cl_2_). Anal. Calcd. for C_14_H_25_N_2_O_3_P × 1.5 H_2_O: C, 51.37; H, 8.62; N, 8.56. Found: C, 51.48; H, 8.36; N, 8.81.

Diethyl (*S*)-3-amino-2-(benzylamino)propylphosphonate [(*S*)-**29**]. According to the general procedure from (*S*)-**27** (0.026 g, 0.08 mmol) and PPh_3_ (0.021 g, 0.08 mmol) (*S*)-2,3-diaminopropylphosphonate (*S*)-**29** (0.018 g, 75%) was obtained as a yellowish oil. ${\left[\alpha \right]}_{D}^{20}$ = −3.70 (c 0.84, CH_2_Cl_2_). Anal. Calcd. for C_14_H_25_N_2_O_3_P × 1.25 H_2_O: C, 52.09; H, 8.59; N, 8.68. Found: C, 52.07; H, 8.83; N, 8.39.

### 3.9. General Procedure for the Ring-Opening Reaction of Diethyl N-(Benzylaziridin-2-yl)methylphosphonates (RS)-**25**, (R)-**25** and (S)-**25** with Acetic Acid

A mixture of (*RS*)-**25**, or (*R*)-**25** or (*S*)-**25** (1.00 mmol) and glacial acetic acid (35.00 mmol) was stirred at room temperature for 24 h. The reaction mixture was concentrated in vacuo with toluene (3 × 10 mL) and then the crude product was chromatographed on a silica gel column with dichloromethane-methanol (100:1, *v*/*v*) to give pure 3-acetoxypropylphosphonates (*RS*)-**28**, or (*R*)-**28** or (*S*)-**28**.

Diethyl (*RS*)-3-acetoxy-2-(benzylamino)propylphosphonate [(*RS*)-**28**]. According to the general procedure from racemic (*RS*)-**25** (0.101 g, 0.36 mmol) and AcOH (0.714 mL, 12.48 mmol) racemic 3-acetoxyphosphonate (*RS*)-**28** (0.094 g, 77%) was obtained as a colorless oil. IR (film): ν = 3457, 3028, 2982, 2926, 1739, 1453, 1239, 1028, 964, 737, 700 cm^−1^. ^1^H NMR (600 MHz, C_6_D_6_): δ = 7.36–7.06 (m, 5H, Ar-*H*), 4.21–4.15 (m, 2H, *H*_a_CH_b_CCP, H_a_C*H*_b_CCP), 3.93–3.80 (m, 4H, 2 × CH_3_C*H*_2_OP), 3.73 (d, *J* = 13.3 Hz, 1H, *H*_a_CH_b_Ph), 3.69 (d, *J* = 13.3 Hz, 1H, H_a_C*H*_b_Ph), 3.25 (ddq, *J* = 12.6 Hz, *J* = 7.6 Hz, *J* = 5.3 Hz, 1H, *H*CCP), 1.91 (ddd, *J* = 18.7 Hz, *J* = 15.3 Hz, *J* = 5.3 Hz, 1H, *H_a_*CH_b_P), 1.86 (ddd, *J* = 17.7 Hz, *J* = 15.3 Hz, *J* = 7.6 Hz, 1H, H_a_C*H*_b_P), 1.61 (s, 3H, C*H*_3_CO), 0.99 (t, *J* = 7.1 Hz, 6H, C*H*_3_CH_2_OP). ^13^C NMR (151 MHz, CDCl_3_): δ = 170.9 (*C*=O), 139.8, 128.5, 128.2, 127.1, 65.9 (d, *J* = 12.2 Hz, *C*CCP), 61.8 (d, *J* = 6.5 Hz, C*C*OP), 51.4 (d, *J* = 3.1 Hz, *C*CP), 51.3, 28.5 (d, *J* = 140.3 Hz, *C*P), 20.9, 16.4 (d, = 6.4 Hz, *C*COP). ^31^P NMR (243 MHz, CDCl_3_): δ = 29.24. Anal. Calcd. for C_16_H_26_NO_5_P × 0.25 H_2_O: C, 55.25; H, 7.68; N, 4.03. Found: C, 55.05; H, 7.92; N, 4.19.

Diethyl (*R*)-3-acetoxy-2-(benzylamino)propylphosphonate [(*R*)-**28**]. According to the general procedure from (*R*)-**25** (0.058 g, 0.205 mmol) and AcOH (0.410 mL, 7.17 mmol) 3-acetoxyphosphonate (*R*)-**28** (0.057 g, 82%) was obtained as a colorless oil.$\;{\left[\alpha \right]}_{D}^{20}$ = –14.40 (*c* 1.00, CH_2_Cl_2_). Anal. Calcd. for C_16_H_26_NO_5_P × 0.5 H_2_O: C, 54.54; H, 7.73; N, 3.98. Found: C, 54.84; H, 7.90; N, 4.17.

Diethyl (*S*)-3-acetoxy-2-(benzylamino)propylphosphonate [(*S*)-**28**]. According to the general procedure from (*S*)-**25** (0.040 g, 0.14 mmol) and AcOH (0.283 mL, 4.94 mmol) 3-acetoxyphosphonate (*S*)-**28** (0.04 g, 83%) was obtained as a colorless oil.$\;{\left[\alpha \right]}_{D}^{20}$ = +19.39 (*c* 0.98, CH_2_Cl_2_). Anal. Calcd. for C_16_H_26_NO_5_P × 0.5 H_2_O: C, 54.54; H, 7.73; N, 3.98. Found: C, 54.79; H, 7.96; N, 4.13.

### 3.10. General Procedure for the Ring-Opening Reaction of 2,3-Epoxypropylphosphonates (RS)-**30**, (S)-**30** and (R)-**30** with (R)-1-Phenylethylamine (Determination of ee)

A mixture of crude 2,3-epoxypropylphosponate (*RS*)-**30**, or (*S*)-**30**, or (*R*)-**30** (1.00 mmol) and (*R*)-1-phenylethylamine (1.10 mmol) in the presence of Ca(OTf)_2_ (0.05 mmol) was stirred at 50 °C for 1 h.

From 2,3-epoxypropylphosponate (*RS*)-**30** (0.062 g, 0.32 mmol) and (*R*)-1-phenylethylamine (0.044 mL, 0.35 mmol) a 1:1 mixture of diethyl (*R*)-2-hydroxy-3-[(*R*)-1-phenylethylamino]propylphosphonate (2*R*,1*R*′)-**42** (δ^31^P = 30.04 ppm) and diethyl (*S*)-2-hydroxy-3-[(*R*)-1-phenylethylamino]propylphosphonate (2*S*,1*R*′)-**42** (δ^31^P = 29.80 ppm) was produced.

From (*S*)-2,3-epoxypropylphosponate (*S*)-**30** (0.020 g, 0.10 mmol) and (*R*)-1-phenylethylamine (0.015 mL, 0.11 mmol) a 98:2 mixture of (2*S*,1*R*′)-**42** (δ^31^P = 29.80 ppm) and (2*R*,1*R*′)-**42** (δ^31^P = 30.04 ppm) was produced, and the enantiomeric purity of the starting epoxide (*S*)-**30** was determined (*ee* = 96%).

From (*R*)-2,3-epoxypropylphosponate (*R*)-**30** (0.031 g, 0.16 mmol) and (*R*)-1-phenylethylamine (0.022 mL, 0.18 mmol) a 96:4 mixture of (2*R*,1*R*′)-**31** (δ^31^P = 30.04 ppm) and (2*S*,1*R*′)-**42** (δ^31^P = 29.80 ppm) was produced, and the enantiomeric purity of the starting epoxide (*R*)-**30** was determined (*ee* = 92%).

## 4. Conclusions

Racemic and enantiomerically enriched (aziridin-2-yl)methylphosphonates **33** were transformed into N-protected derivatives **24** (N-Boc) and **25** (N-Bn), which were then used as convenient substrates for the synthesis of propylphosphonates respectively functionalized with amino and hydroxyl groups at C2 and C3.

While catalytic hydrogenation of *N*-Boc-aziridine **24** gave diethyl 2-(*tert*-butoxycarbonylamino)propylphosphonate **26** as a single product, nucleophilic ring-opening reactions in **24** with both trimethylsilyl azide and acetic acid occurred at C2 and C3 atoms of the aziridine ring leading to the formation of two regioismers, i.e., 2-amino-3-azidopropylphosphonate **34** and 3-amino-2-azidopropylphosphonate **35** from **24** and trimethylsilyl azide, and 3-acetoxy-2-aminopropylphosphonate **36** and 2-acetoxy-3-aminopropylphosphonate **37** from **24** and acetic acid. The reaction of *N*-Bn-aziridine **25** with both trimethylsilyl azide and acetic acid proceeded also regiospecifically with the opening of aziridine ring at less-hindered C3 atom to give 3-azido-2-benzylaminopropylphosphonate **27** and 3-acetoxy-2-benzylaminopropylphosphonate **28**, respectively.

Enantiomerically enriched (aziridin-2-yl)methylphosphonates (*R*)-**33** and (*S*)-**33** were obtained from racemic epoxide **30** via its hydrolytic kinetic resolution (HKR) followed by epoxide ring-opening in (*S*)-**30** and (*R*)-**30** with sodium azide and further transformation of 3-azido-2-hydroxypropylphosphonates (*S*)-**31** and (*R*)-**31** into (aziridin-2-yl)methylphosphonates (*R*)- and (*S*)-**33**. (Aziridin-2-yl)methylphosphonates (*R*)-**33** and (*S*)-**33** were successfully converted into respectively protected derivatives (*R*)-**24** and (*S*)-**24** (N-Boc) as well as (*R*)-**25** and (*S*)-**25** (N-Bn).

Enantiomerically enriched 2-(*N*-Boc-amino)propylphosphonates (*R*)-**26** and (*S*)-**26** were efficiently obtained from *N*-Boc-(aziridin-2-yl)methylphosphonates (*R*)-**24** and (*S*)-**24**, while *N*-Bn-(aziridin-2-yl)methylphosphonates (*R*)-**25** and (*S*)-**25** was used effective preparation of N-Boc-2,3-diaminopropylphosphonates (*R*)-**29** and (*S*)-**29** as well as 2-(*N*-Bn-amino)-3-acetoxypropylphosphonates (*R*)-**28** and (S)-**28**.

## Data Availability

Data is contained within the article.

## Supplementary Materials

The following supporting information can be downloaded at https://www.mdpi.com/article/10.3390/molecules28031466/s1. Figure S1: ^1^H NMR Spectrum for racemic **24** in CDCl_3_; Figure S2: ^13^C NMR Spectrum for racemic **24** in CDCl_3_; Figure S3: ^31^P NMR Spectrum for racemic **24** in CDCl_3_; Figure S4: ^1^H NMR Spectrum for (*R*)-**24** in CDCl_3_; Figure S5: ^31^P NMR Spectrum for (*R*)-**24** in CDCl_3_; Figure S6: ^1^H NMR Spectrum for (*S*)-**24** in CDCl_3_; Figure S7: ^31^P NMR Spectrum for (*S*)-**24** in CDCl_3_; Figure S8: ^1^H NMR Spectrum for racemic **25** in CDCl_3_; Figure S9: ^13^C NMR Spectrum for racemic **25** in CDCl_3_; Figure S10: ^31^P NMR Spectrum for racemic **25** in CDCl_3_; Figure S11: ^1^H NMR Spectrum for (*R*)-**25** in CDCl_3_; Figure S12: ^31^P NMR Spectrum for (*R*)-**25** in CDCl_3_; Figure S13: ^1^H NMR Spectrum for (*S*)-**25** in CDCl_3_; Figure S14: ^31^P NMR Spectrum for (*S*)-**25** in CDCl_3_; Figure S15: ^1^H NMR Spectrum for racemic **26** in CDCl_3_; Figure S16: ^13^C NMR Spectrum for racemic **26** in CDCl_3_; Figure S17: ^31^P NMR Spectrum for racemic **26** in CDCl_3_; Figure S18: ^1^H NMR Spectrum for (*R*)-**26** in CDCl_3_; Figure S19: ^31^P NMR Spectrum for (*R*)-**26** in CDCl_3_; Figure S20: ^1^H NMR Spectrum for (*S*)-**26** in CDCl_3_; Figure S21: ^31^P NMR Spectrum for (*S*)-**26** in CDCl_3_; Figure S22: ^1^H NMR Spectrum for racemic **27** in CDCl_3_; Figure S23: ^13^C NMR Spectrum for racemic **27** in CDCl_3_; Figure S24: ^31^P NMR Spectrum for racemic **27** in CDCl_3_; Figure S25: ^1^H NMR Spectrum for (*R*)-**27** in CDCl_3_; Figure S26: ^31^P NMR Spectrum for (*R*)-**27** in CDCl_3_; Figure S27: ^1^H NMR Spectrum for (*S*)-**27** in CDCl_3_; Figure S28: ^31^P NMR Spectrum for (*S*)-**27** in CDCl_3_; Figure S29: ^1^H NMR Spectrum for racemic **28** in CDCl_3_; Figure S30: ^13^C NMR Spectrum for racemic **28** in CDCl_3_; Figure S31: ^31^P NMR Spectrum for racemic **28** in CDCl_3_; Figure S32: ^1^H NMR Spectrum for (*R*)-**28** in CDCl_3_; Figure S33: ^31^P NMR Spectrum for (*R*)-**28** in CDCl_3_; Figure S34: ^1^H NMR Spectrum for (*S*)-**28** in CDCl_3_; Figure S35: ^31^P NMR Spectrum for (*S*)-**28** in CDCl_3_; Figure S36: ^1^H NMR Spectrum for racemic **29** in CDCl_3_; Figure S37: ^13^C NMR Spectrum for racemic **29** in CDCl_3_; Figure S38: ^31^P NMR Spectrum for racemic **29** in CDCl_3_; Figure S39: ^1^H NMR Spectrum for (*R*)-**29** in CDCl_3_; Figure S40: ^31^P NMR Spectrum for (*R*)-**29** in CDCl_3_; Figure S41: ^1^H NMR Spectrum for (*S*)-**29** in CDCl_3_; Figure S42: ^31^P NMR Spectrum for (*S*)-**29** in CDCl_3_; Figure S43: ^1^H NMR Spectrum for racemic **34** in CDCl_3_; Figure S44: ^13^C NMR Spectrum for racemic **34** in CDCl_3_; Figure S45: ^31^P NMR Spectrum for racemic **34** in CDCl_3_; Figure S46: ^1^H NMR Spectrum for racemic **35** in CDCl_3_; Figure S47: ^13^C NMR Spectrum for racemic **35** in CDCl_3_; Figure S48: ^31^P NMR Spectrum for racemic **35** in CDCl_3_; Figure S49: ^1^H NMR Spectrum for (*E*)-**39** in CDCl_3_; Figure S50: ^13^C NMR Spectrum for (*E*)-**39** in CDCl_3_; Figure S51: ^31^P NMR Spectrum for (*E*)-**39** in CDCl_3_; Figure S52: ^31^P NMR Spectrum for crude mixture after reaction of racemic **30** with (*R*)-1-phenylethylamine in CDCl_3_; Figure S53: ^31^P NMR Spectrum for crude mixture after reaction of (*S*)-**30** with (*R*)-1-phenylethylamine in CDCl_3_; Figure S54: ^31^P NMR Spectrum for crude mixture after reaction of (*R*)-**30** with (*R*)-1-phenylethylamine in CDCl_3_; Figure S55: ^31^P NMR Spectrum for the mixture of crude (*S*)-**31** with 4 equiv: of quinine in CDCl_3_; Figure S56: ^31^P NMR Spectrum for the mixture of crude (*R*)-**31** with 4 equiv: of quinine in CDCl_3_; Figure S57: Analytical chromatogram for racemic **27**; Figure S58: Analytical chromatogram for (*R*)-**27**; Figure S59: Analytical chromatogram for (*S*)-**27**; Figure S60: Analytical chromatogram for racemic **28**; Figure S61: Analytical chromatogram for (*R*)-**28**; Figure S62: Analytical chromatogram for (*S*)-**28**.

## Sample Availability

Samples are not available.
