# Supplementary material for: Synthesis of Enantiomerically Enriched Protected 2-Amino-, 2,3-Diamino- and 2-Amino-3-Hydroxypropylphosphonates
Source: Molecules. 2023 Feb 2;28(3):1466. doi: 10.3390/molecules28031466 (PMC9921368; doi:10.3390/molecules28031466)

Synthesis of Enantiomerically Enriched Protected 2-Amino-, 2,3-Diamino- and 2-Amino-3-Hydroxypropylphosphonates

Aleksandra Trocha [1], Dorota G. Piotrowska [1] and Iwona E. Głowacka [1,*]

Bioorganic Chemistry Laboratory, Faculty of Pharmacy, Medical University of Lodz, 90-151 Lodz, Muszynskiego 1, Poland

*Correspondence: iwona.glowacka@umed.lodz.pl (I.E.G.)

Contents

NMR spectra for compounds **24**, (*R*)-**24**, (*S*)-**24**, **25**, (*R*)-**25**, (*S*)-**25**, **26**, (*R*)-**26**, (*S*)-**26**, **27**, (*R*)-**27**, (*S*)-**27**, **28**, (*R*)-**28**, (*S*)-**28**, **29**, (*R*)-**29**, (*S*)-**29**, **34**, **35** and (*E*)-**39.**

NMR spectra for crude mixtures after reactions of racemic **30**, (*S*)-**30,** and (*R*)-**30** with (*R*)-1-phenylethylamine and for crude mixtures of (*S*)-**31** and (*R*)-**31** with quinine.

Analytical chromatograms for **27**, (*R*)-**27**, (*S*)-**27**, **28**, (*R*)-**28**, and (*S*)-**28**.

Figure S1. ^{1}H NMR Spectrum for racemic **24** in $CDCl_3$

Figure S2. ^{13}C NMR Spectrum for racemic **24** in $CDCl_3$

Figure S3. ^{31}P NMR Spectrum for racemic **24** in $CDCl_3$

Figure S4. ^{1}H NMR Spectrum for (*R*)-**24** in $CDCl_3$

Figure S5. ^{31}P NMR Spectrum for (*R*)-**24** in $CDCl_3$

Figure S6. ^{1}H NMR Spectrum for (*S*)-**24** in $CDCl_3$

Figure S7. ^{31}P NMR Spectrum for (*S*)-**24** in $CDCl_3$

Figure S8. ^{1}H NMR Spectrum for racemic **25** in $CDCl_3$

Figure S9. ^{13}C NMR Spectrum for racemic **25** in $CDCl_3$

Figure S10. ^{31}P NMR Spectrum for racemic **25** in $CDCl_3$

Figure S11. ^{1}H NMR Spectrum for (*R*)-**25** in $CDCl_3$

Figure S12. ^{31}P NMR Spectrum for (*R*)-**25** in $CDCl_3$

Figure S13. ^{1}H NMR Spectrum for (*S*)-**25** in $CDCl_3$

Figure S14. ^{31}P NMR Spectrum for (*S*)-**25** in $CDCl_3$

Figure S15. ^{1}H NMR Spectrum for racemic **26** in $CDCl_3$

Figure S16. ^{13}C NMR Spectrum for racemic **26** in $CDCl_3$

Figure S17. ^{31}P NMR Spectrum for racemic **26** in $CDCl_3$

Figure S18. ^{1}H NMR Spectrum for (*R*)-**26** in $CDCl_3$

Figure S19. ^{31}P NMR Spectrum for (*R*)-**26** in $CDCl_3$

Figure S20. ^{1}H NMR Spectrum for (*S*)-**26** in $CDCl_3$

Figure S21. ^{31}P NMR Spectrum for (*S*)-**26** in $CDCl_3$

Figure S22. ^{1}H NMR Spectrum for racemic **27** in $CDCl_3$

Figure S23. ^{13}C NMR Spectrum for racemic **27** in $CDCl_3$

Figure S24. ^{31}P NMR Spectrum for racemic **27** in $CDCl_3$

Figure S25. ^{1}H NMR Spectrum for (*R*)-**27** in $CDCl_3$

Figure S26. ^{31}P NMR Spectrum for (*R*)-**27** in $CDCl_3$

Figure S27. ^{1}H NMR Spectrum for (*S*)-**27** in $CDCl_3$

Figure S28. ^{31}P NMR Spectrum for (*S*)-**27** in $CDCl_3$

Figure S29. ^{1}H NMR Spectrum for racemic **28** in $CDCl_3$

Figure S30. ^{13}C NMR Spectrum for racemic **28** in $CDCl_3$

Figure S31. ^{31}P NMR Spectrum for racemic **28** in $CDCl_3$

Figure S32. ^{1}H NMR Spectrum for (*R*)-**28** in $CDCl_3$

Figure S33. ^{31}P NMR Spectrum for (*R*)-**28** in $CDCl_3$

Figure S34. ^{1}H NMR Spectrum for (*S*)-**28** in $CDCl_3$

Figure S35. ^{31}P NMR Spectrum for (*S*)-**28** in $CDCl_3$

Figure S36. ^{1}H NMR Spectrum for racemic **29** in $CDCl_3$

Figure S37. ^{13}C NMR Spectrum for racemic **29** in $CDCl_3$

Figure S38. ^{31}P NMR Spectrum for racemic **29** in $CDCl_3$

Figure S39. ^{1}H NMR Spectrum for (*R*)-**29** in $CDCl_3$

Figure S40. ^{31}P NMR Spectrum for (*R*)-**29** in $CDCl_3$

Figure S41. ^{1}H NMR Spectrum for (*S*)-**29** in $CDCl_3$

Figure S42. ^{31}P NMR Spectrum for (*S*)-**29** in $CDCl_3$

Figure S43. ^{1}H NMR Spectrum for racemic **34** in $CDCl_3$

Figure S44. ^{13}C NMR Spectrum for racemic **34** in $CDCl_3$

Figure S45. ^{31}P NMR Spectrum for racemic **34** in $CDCl_3$

Figure S46. ^{1}H NMR Spectrum for racemic **35** in $CDCl_3$

Figure S47. ^{13}C NMR Spectrum for racemic **35** in $CDCl_3$

Figure S48. ^{31}P NMR Spectrum for racemic **35** in $CDCl_3$

Figure S49. ^{1}H NMR Spectrum for (*E*)-**39** in $CDCl_3$

Figure S50. ^{13}C NMR Spectrum for (*E*)-**39** in $CDCl_3$

Figure S51. ^{31}P NMR Spectrum for (*E*)-**39** in $CDCl_3$

Figure S52. ^{31}P NMR Spectrum for crude mixture after reaction of racemic **30** with (*R*)-1-phenylethylamine in $CDCl_3$

Figure S53. ^{31}P NMR Spectrum for crude mixture after reaction of (*S*)-**30** with (*R*)-1-phenylethylamine in $CDCl_3$

Figure S54. ^{31}P NMR Spectrum for crude mixture after reaction of (*R*)-**30** with (*R*)-1-phenylethylamine in $CDCl_3$

Figure S55. ^{31}P NMR Spectrum for the mixture of crude (*S*)-**31** with 4 equiv. of quinine in $CDCl_3$

Figure S56. ^{31}P NMR Spectrum for the mixture of crude (*R*)-**31** with 4 equiv. of quinine in $CDCl_3$

Figure S57. Analytical chromatogram for racemic **27**

Figure S58. Analytical chromatogram for (*R*)-**27**

Figure S59. Analytical chromatogram for (*S*)-**27**

Figure S60. Analytical chromatogram for racemic **28**

Figure S61. Analytical chromatogram for (*R*)-**28**

Figure S62. Analytical chromatogram for (*S*)-**28**

Figure S1. ^{1}H NMR Spectrum for racemic **24** in $CDCl_3$

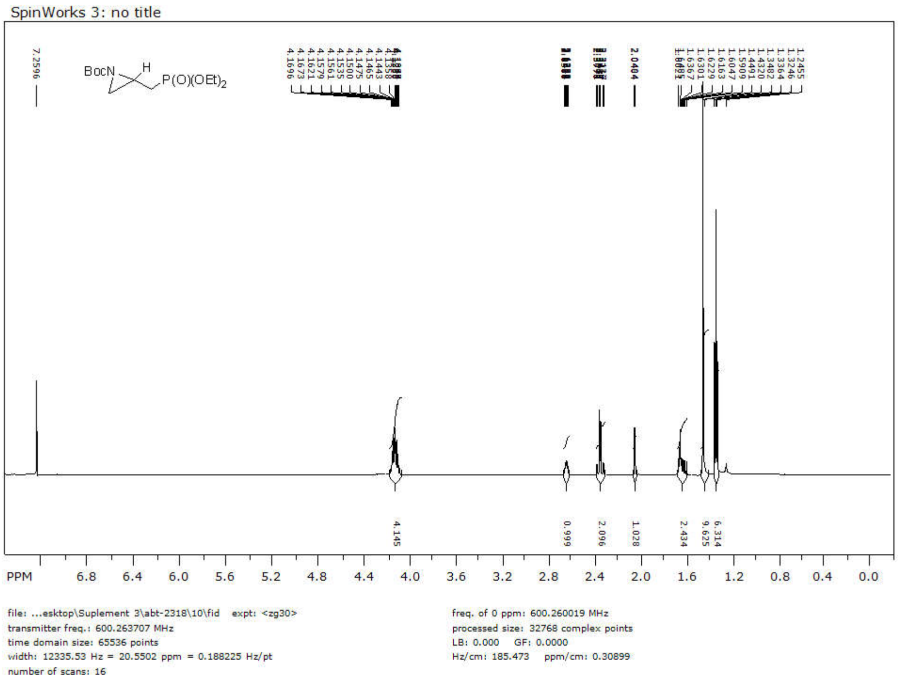

Figure S2. ^{13}C NMR Spectrum for racemic **24** in $CDCl_3$

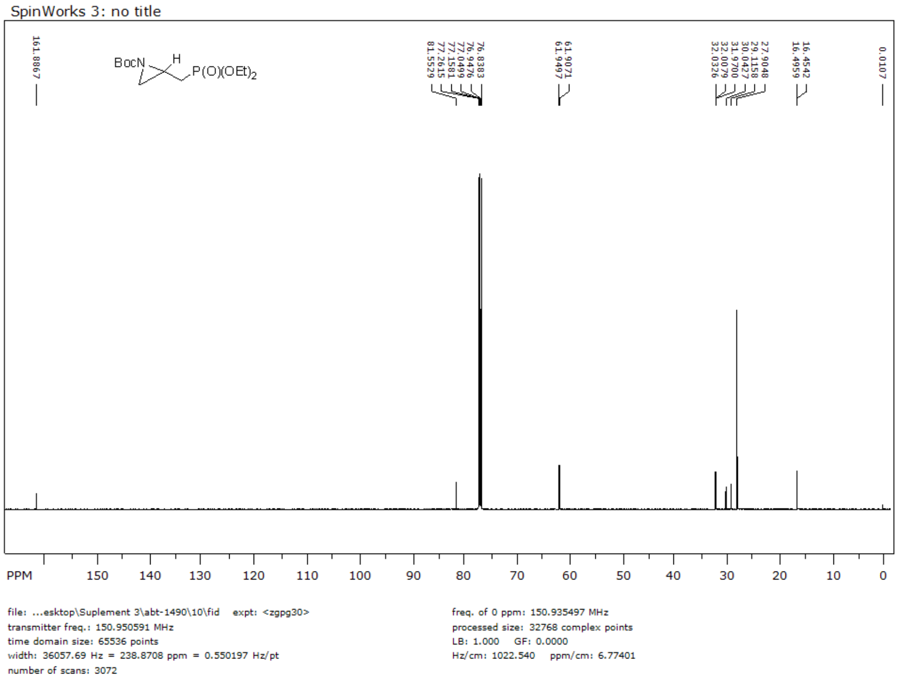

Figure S3. ^{31}P NMR Spectrum for racemic **24** in $CDCl_3$

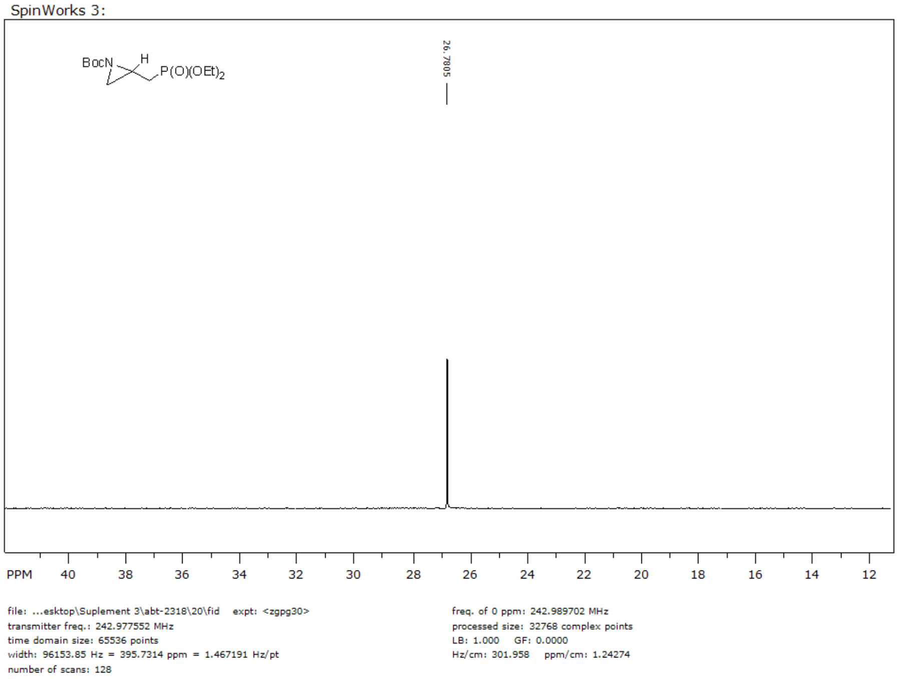

Figure S4. ^{1}H NMR Spectrum for (*R*)-**24** in $CDCl_3$

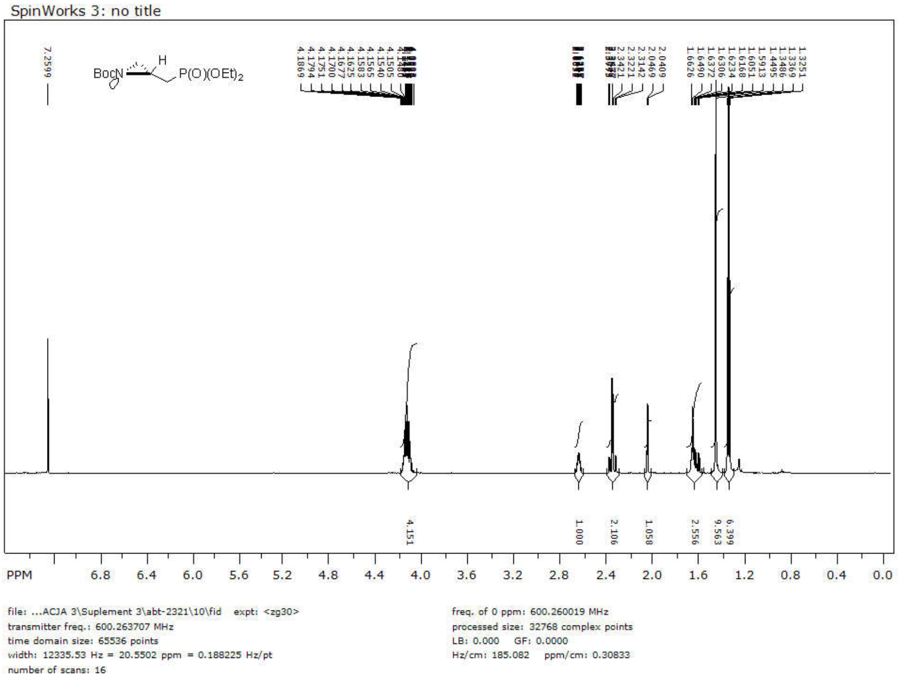

Figure S5. ^{31}P NMR Spectrum for (*R*)-**24** in $CDCl_3$

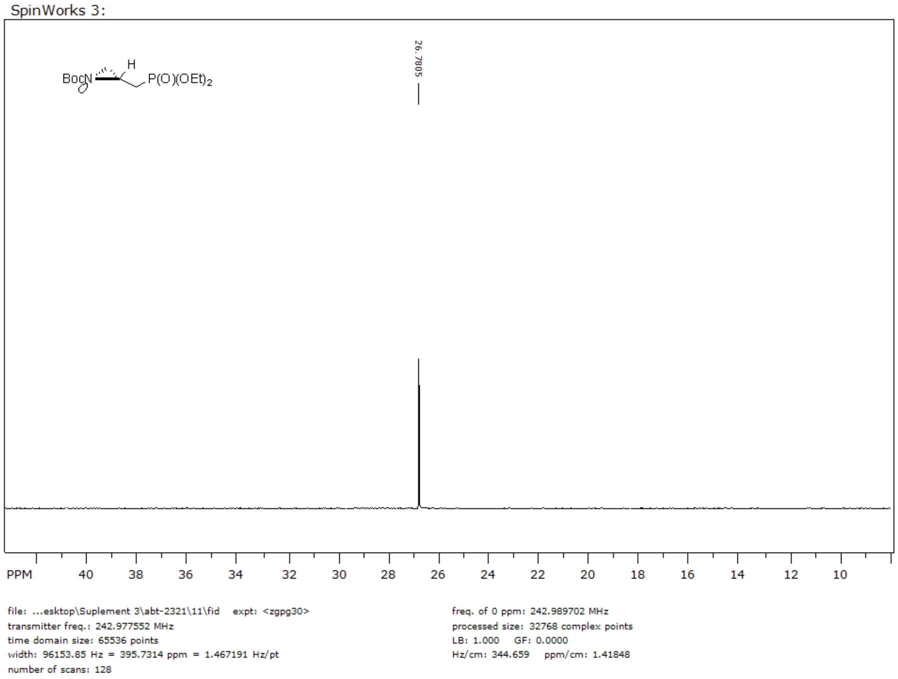

Figure S6. ^{1}H NMR Spectrum for (*S*)-**24** in $CDCl_3$

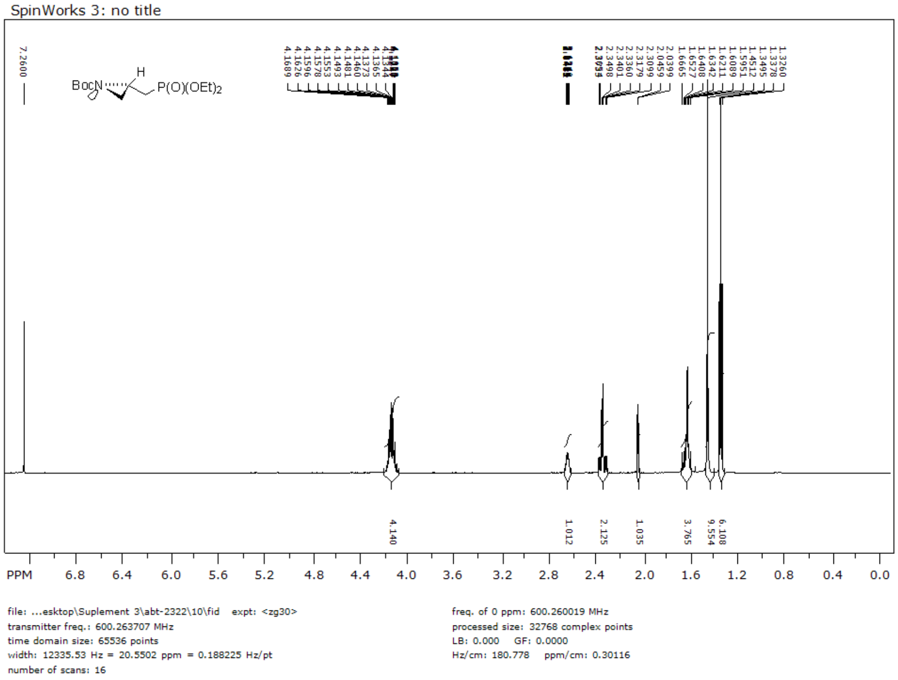

Figure S7. ^{31}P NMR Spectrum for (*S*)-**24** in $CDCl_3$

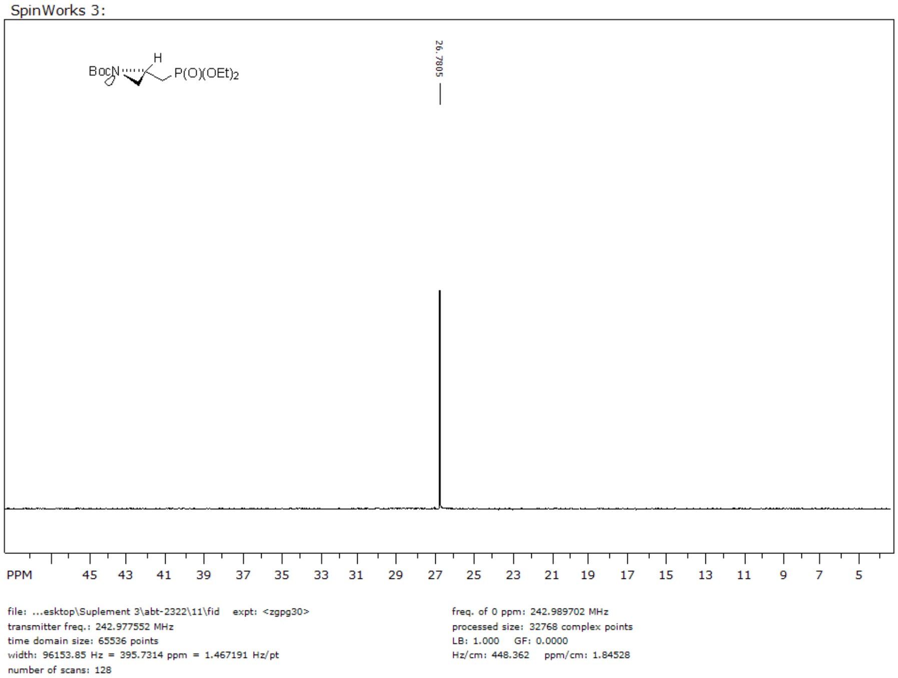

Figure S8. ^{1}H NMR Spectrum for racemic **25** in $CDCl_3$

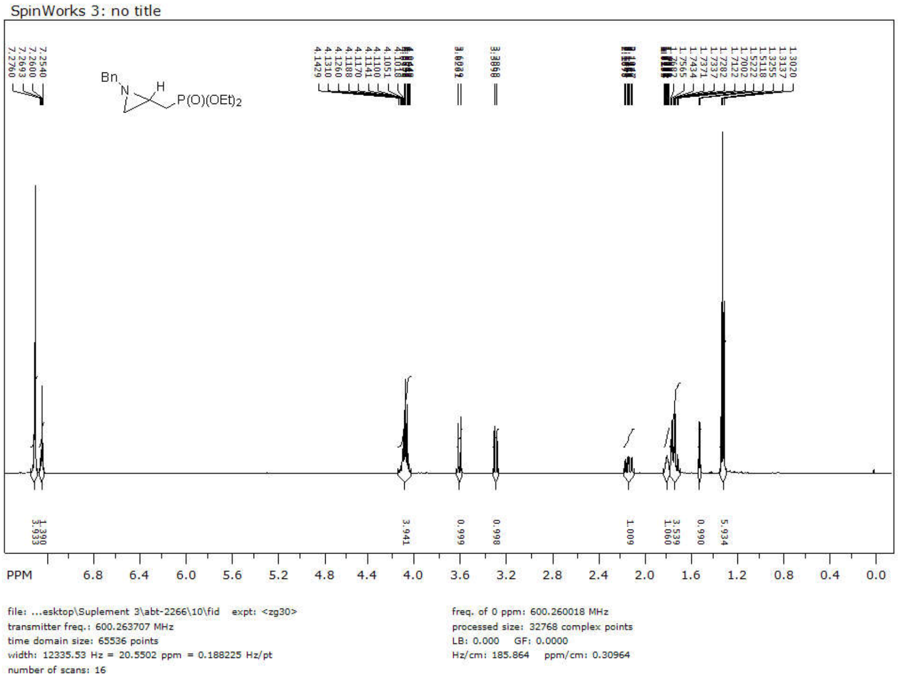

Figure S9. ^{13}C NMR Spectrum for racemic **25** in $CDCl_3$

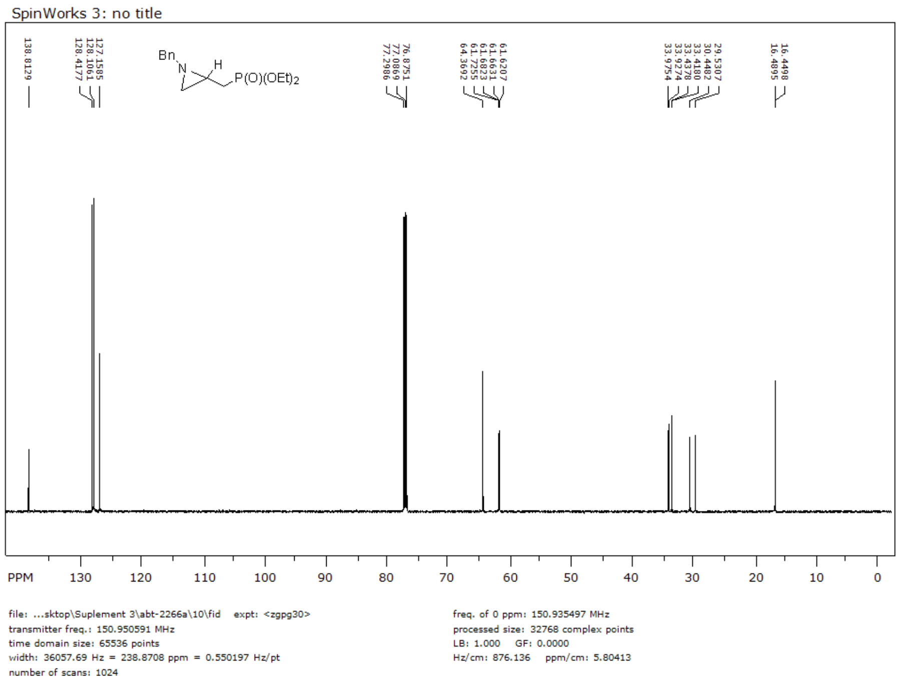

Figure S10. ^{31}P NMR Spectrum for racemic **25** in $CDCl_3$

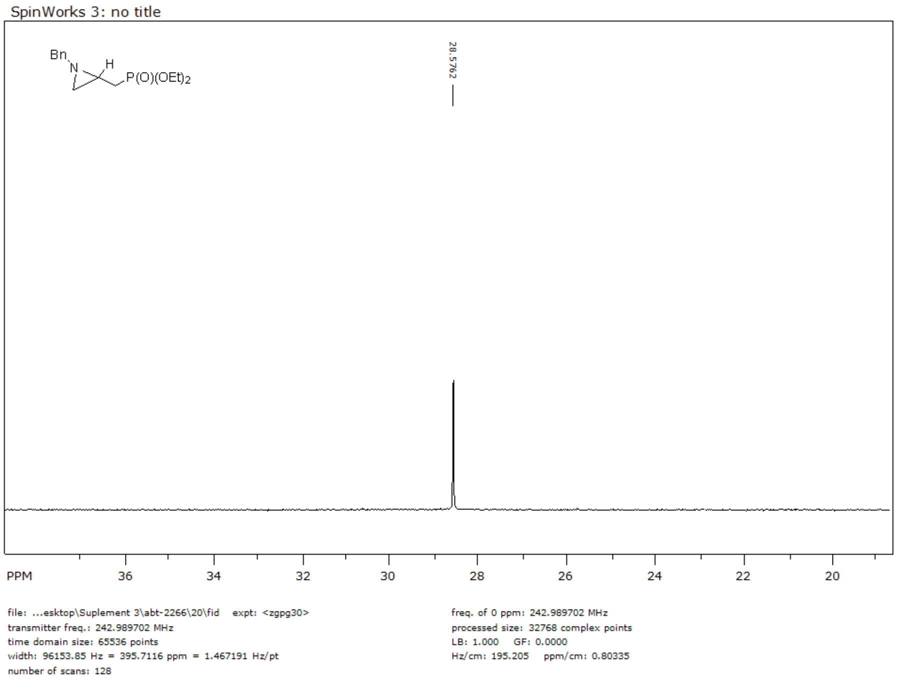

Figure S11. ^{1}H NMR Spectrum for (R)-**25** in $CDCl_3$

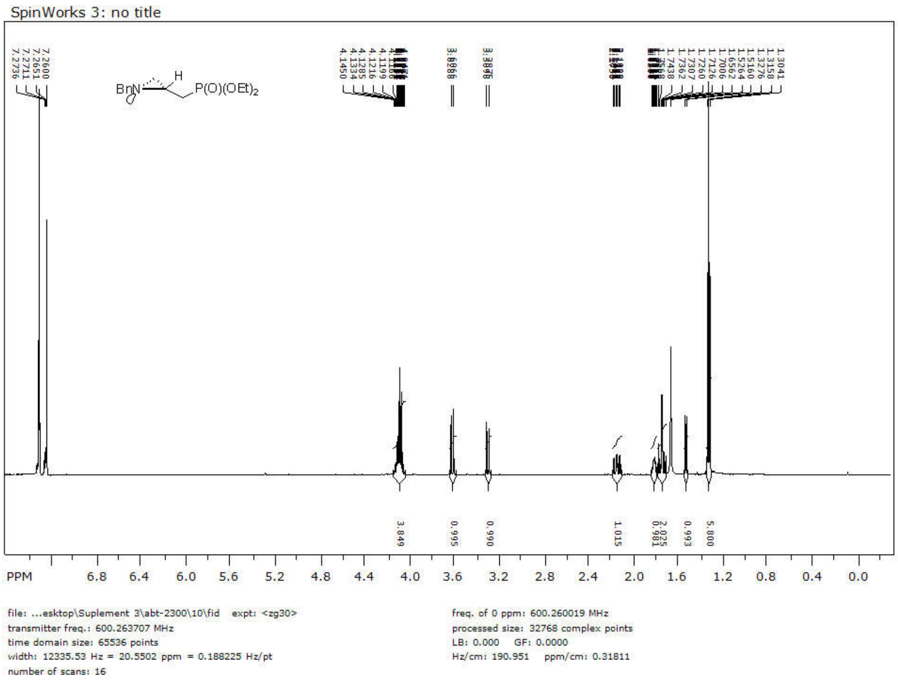

Figure S12. ^{31}P NMR Spectrum for (R)-**25** in $CDCl_3$

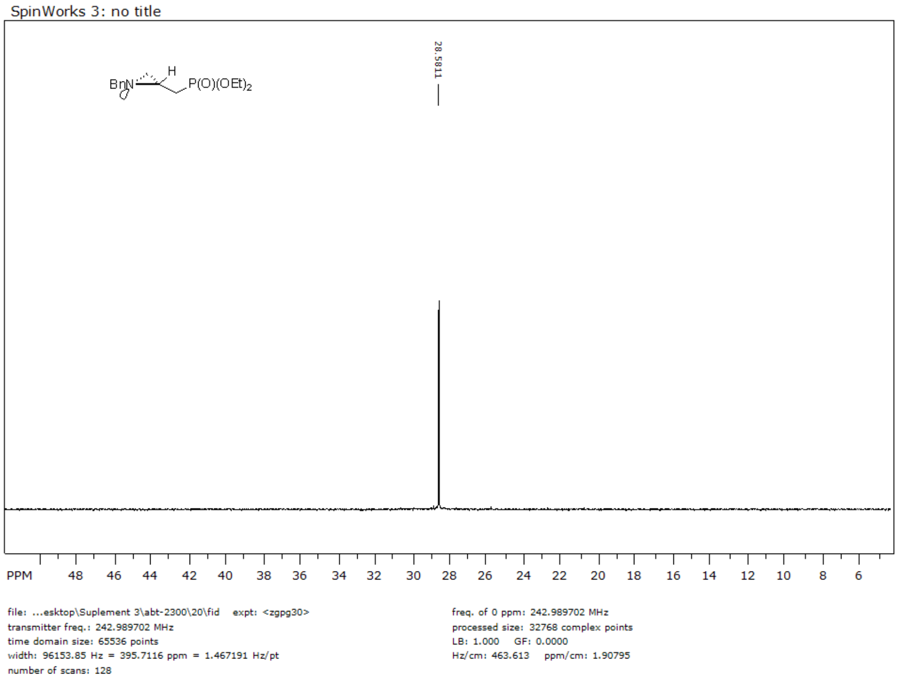

Figure S13. ^{1}H NMR Spectrum for (*S*)-**25** in $CDCl_3$

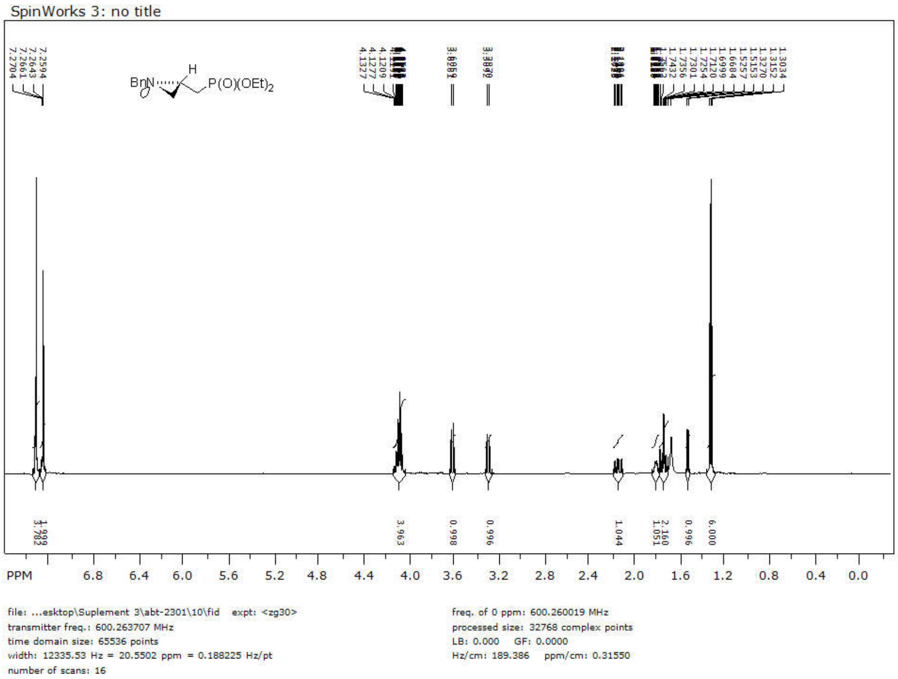

Figure S14. ^{31}P NMR Spectrum for (*S*)-**25** in $CDCl_3$

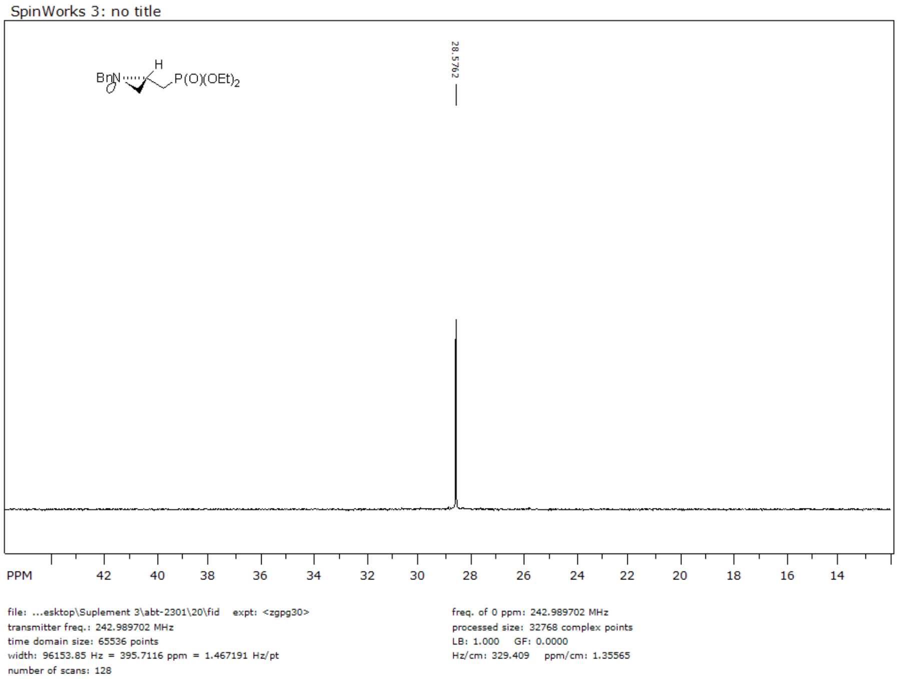

Figure S15. ^{1}H NMR Spectrum for racemic **26** in $CDCl_3$

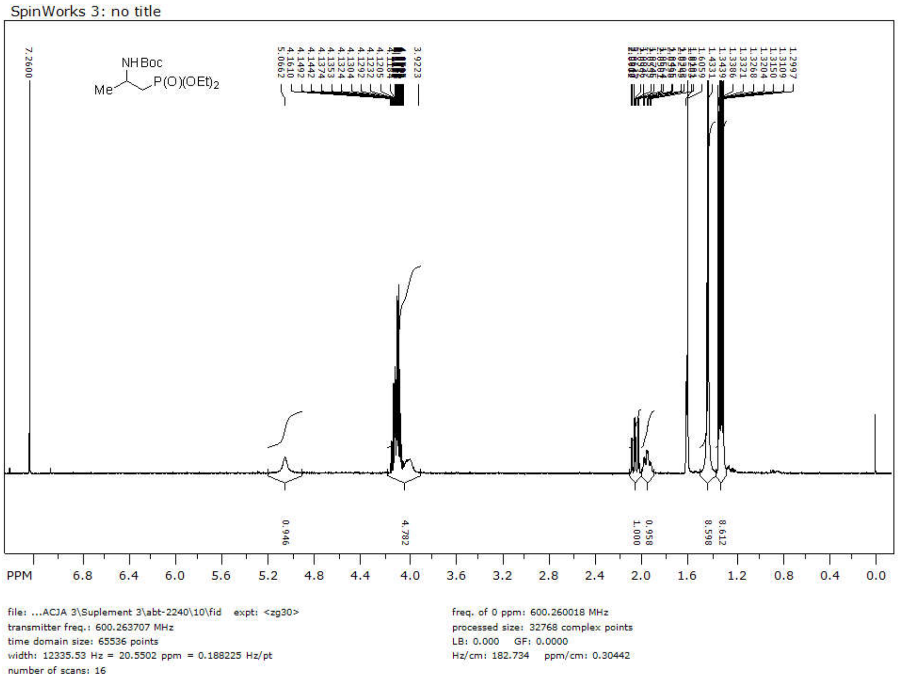

Figure S16. ^{13}C NMR Spectrum for racemic **26** in $CDCl_3$

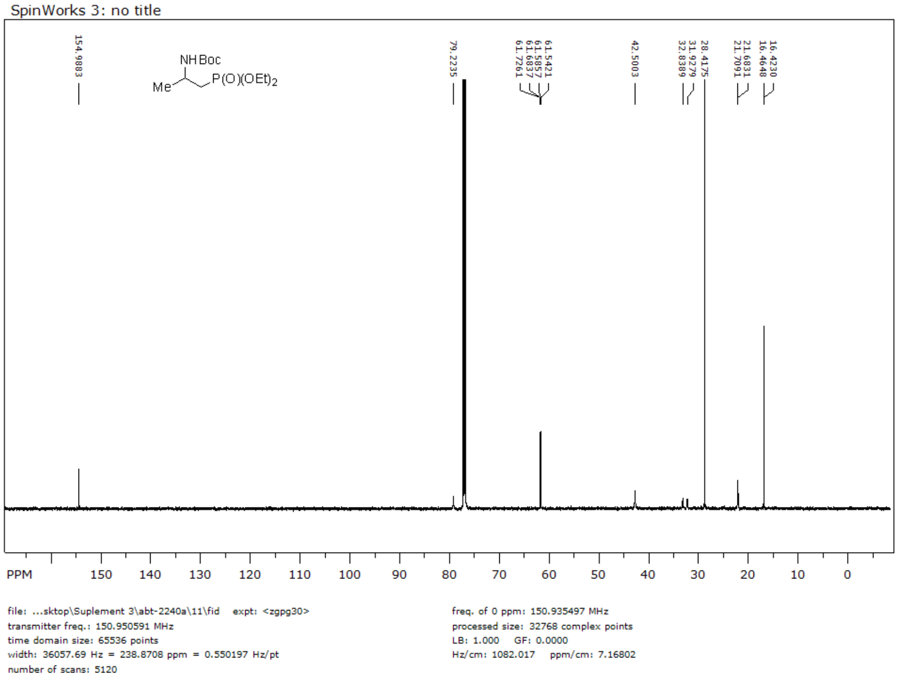

Figure S17. ^{31}P NMR Spectrum for racemic **26** in $CDCl_3$

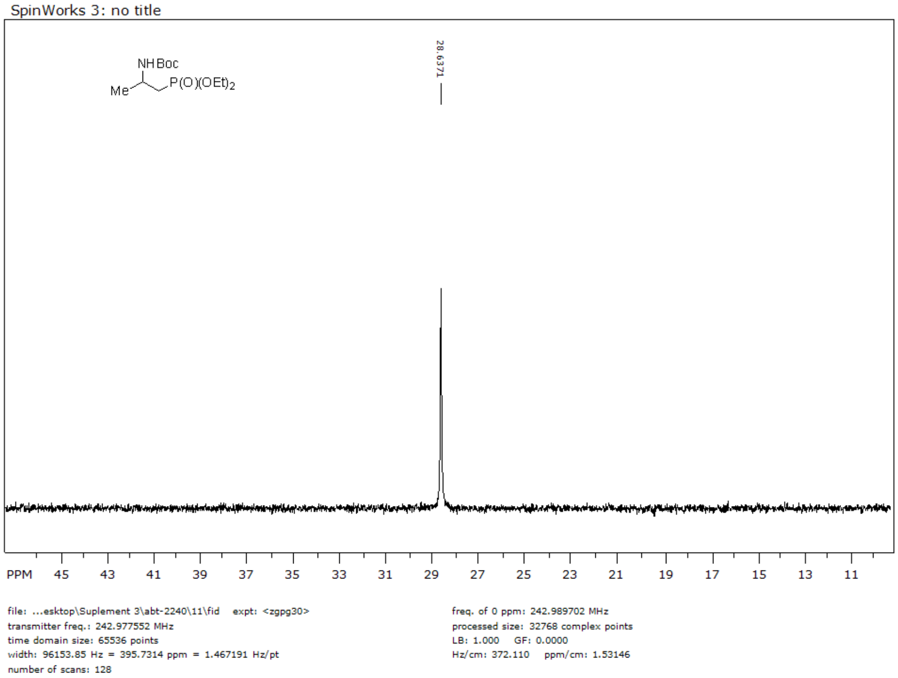

Figure S18. ^{1}H NMR Spectrum for (*R*)-**26** in $CDCl_3$

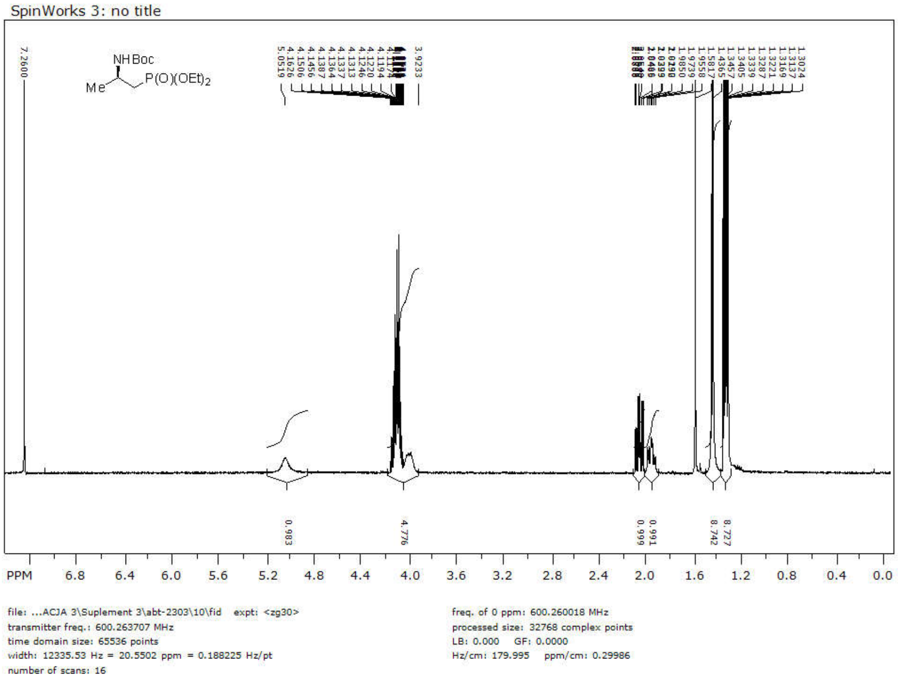

Figure S19. ^{31}P NMR Spectrum for (*R*)-**26** in $CDCl_3$

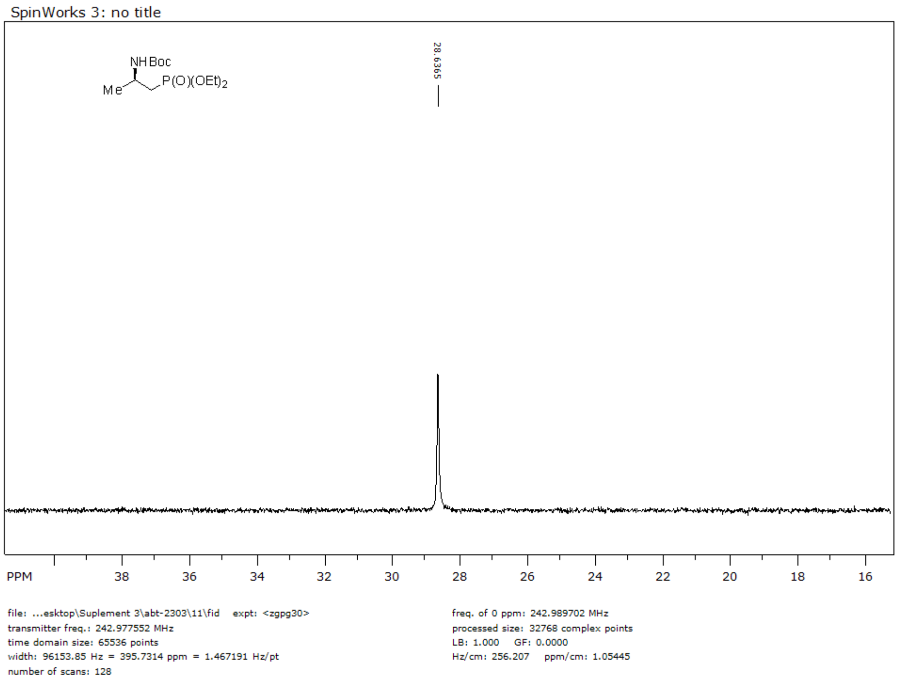

Figure S20. ^{1}H NMR Spectrum for (*S*)-**26** in $CDCl_3$

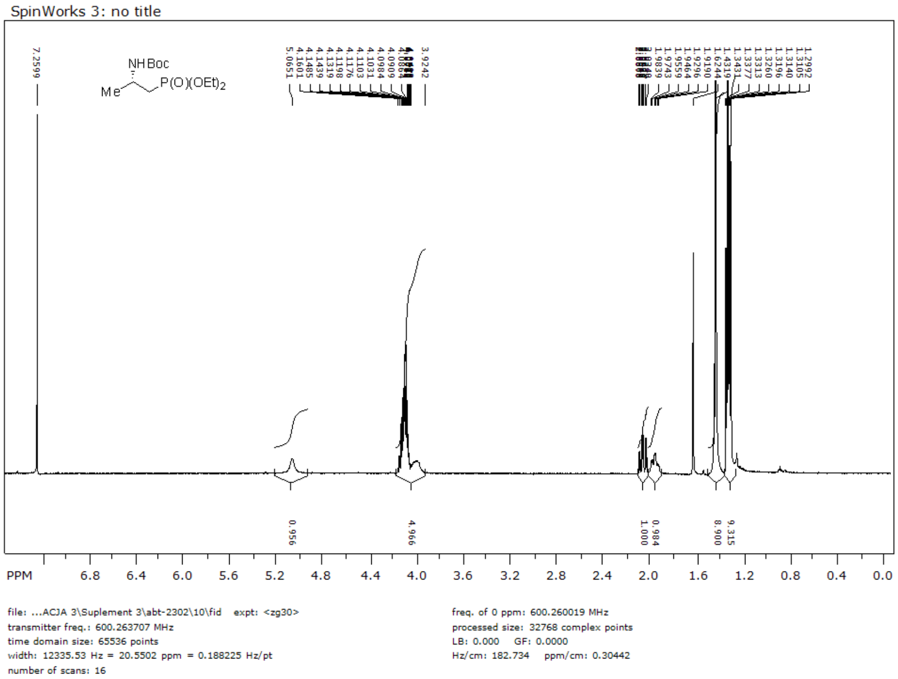

Figure S21. ^{31}P NMR Spectrum for (*S*)-**26** in $CDCl_3$

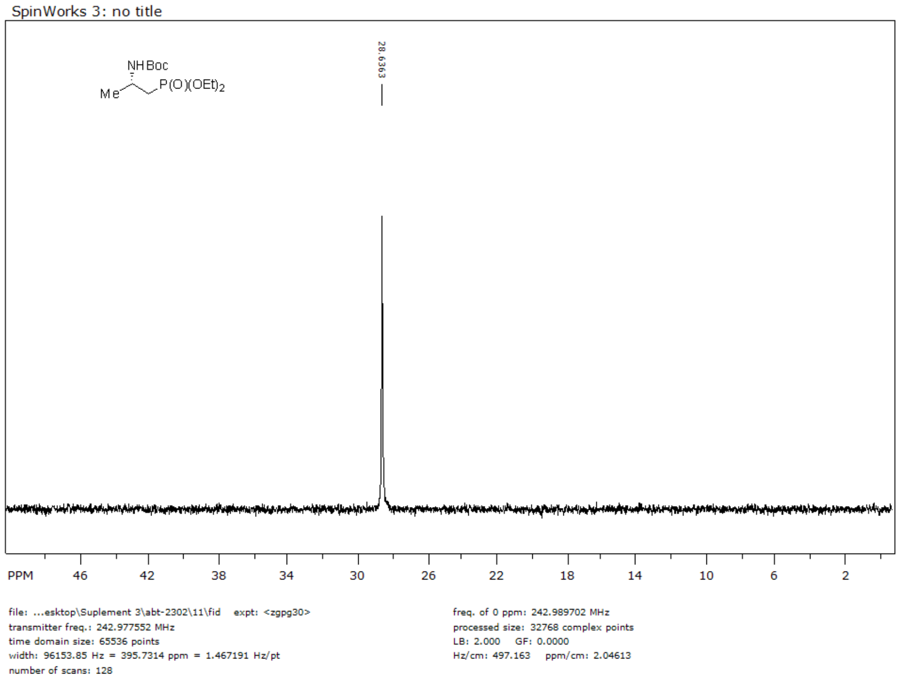

Figure S22. ^{1}H NMR Spectrum for racemic **27** in $CDCl_3$

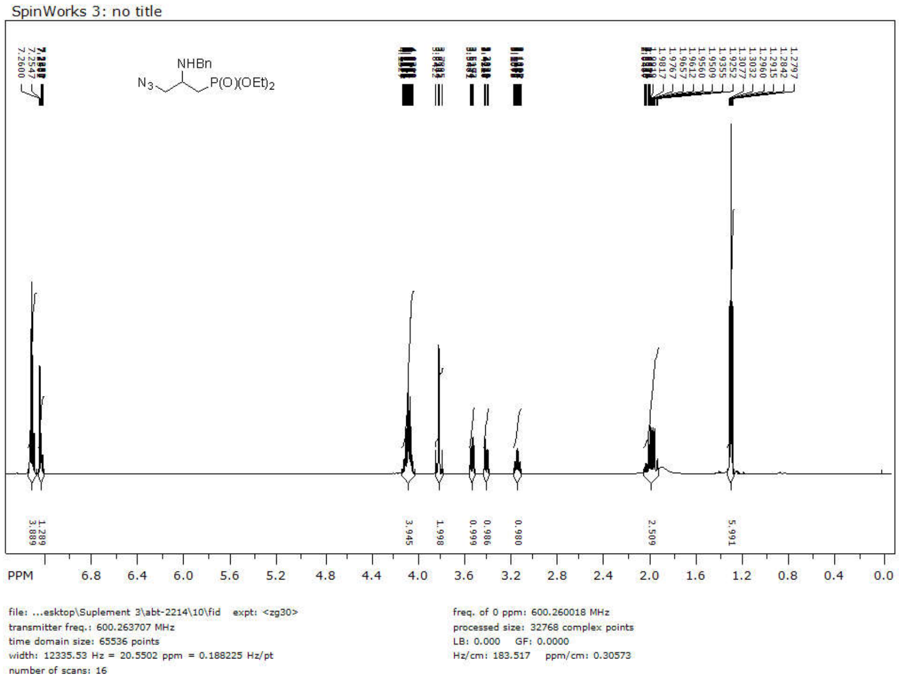

Figure S23. ^{13}C NMR Spectrum for racemic **27** in $CDCl_3$

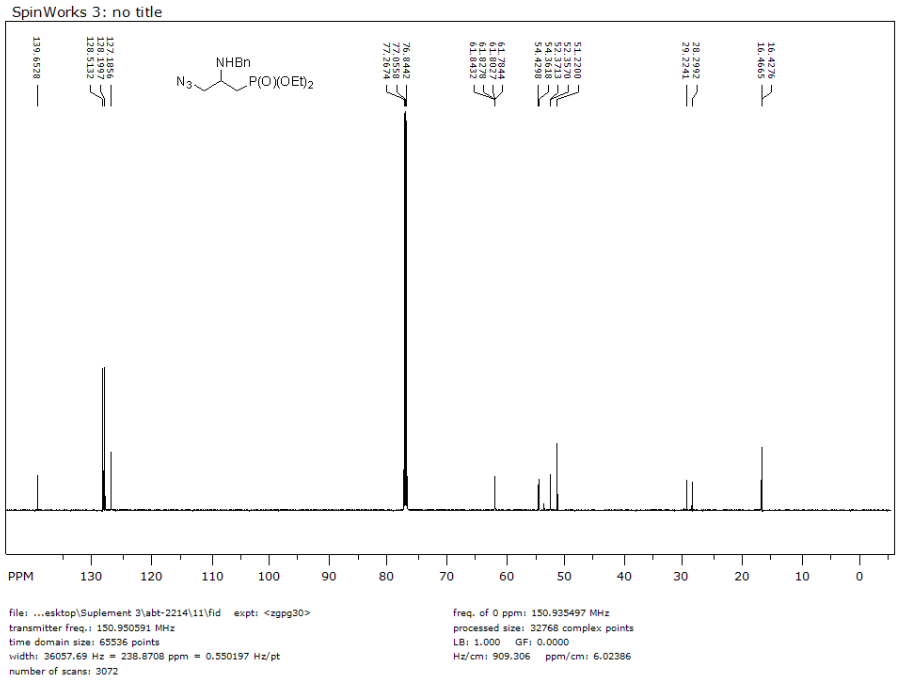

Figure S24. ^{31}P NMR Spectrum for racemic **27** in $CDCl_3$

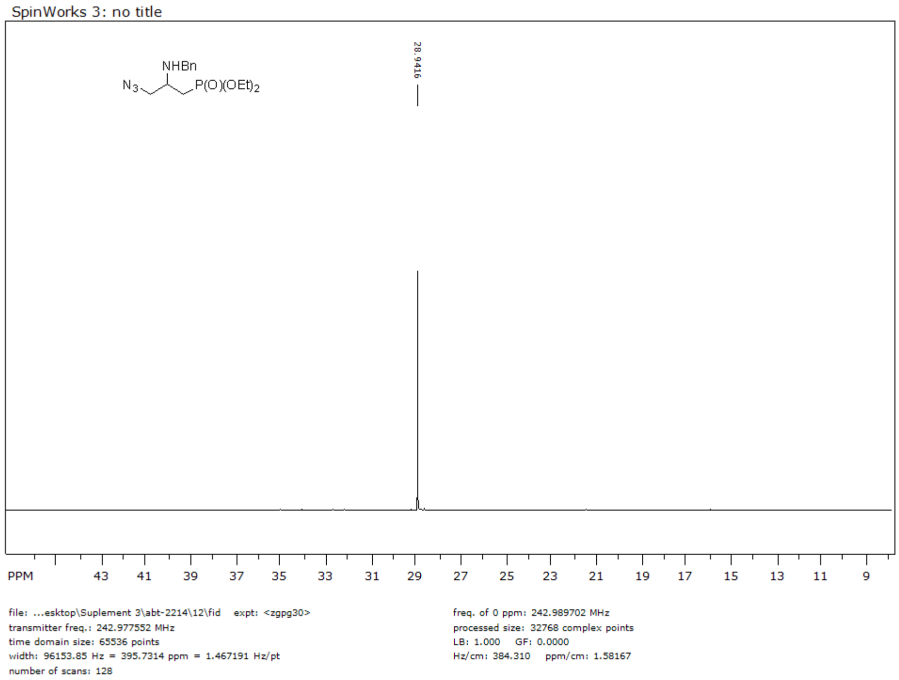

Figure S25. ^{1}H NMR Spectrum for (*R*)-**27** in $CDCl_3$

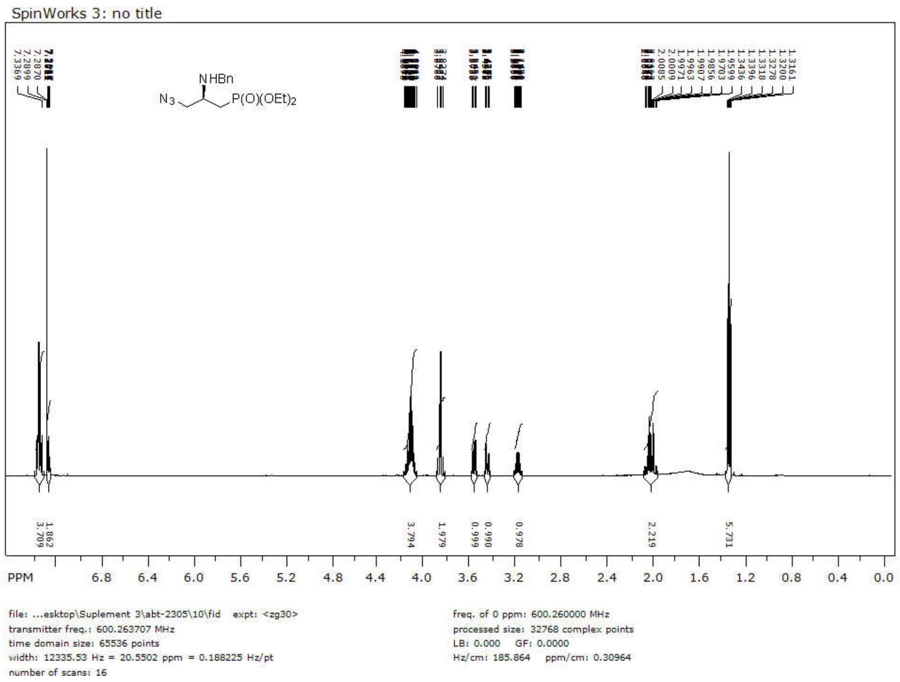

Figure S26. ^{31}P NMR Spectrum for (*R*)-**27** in $CDCl_3$

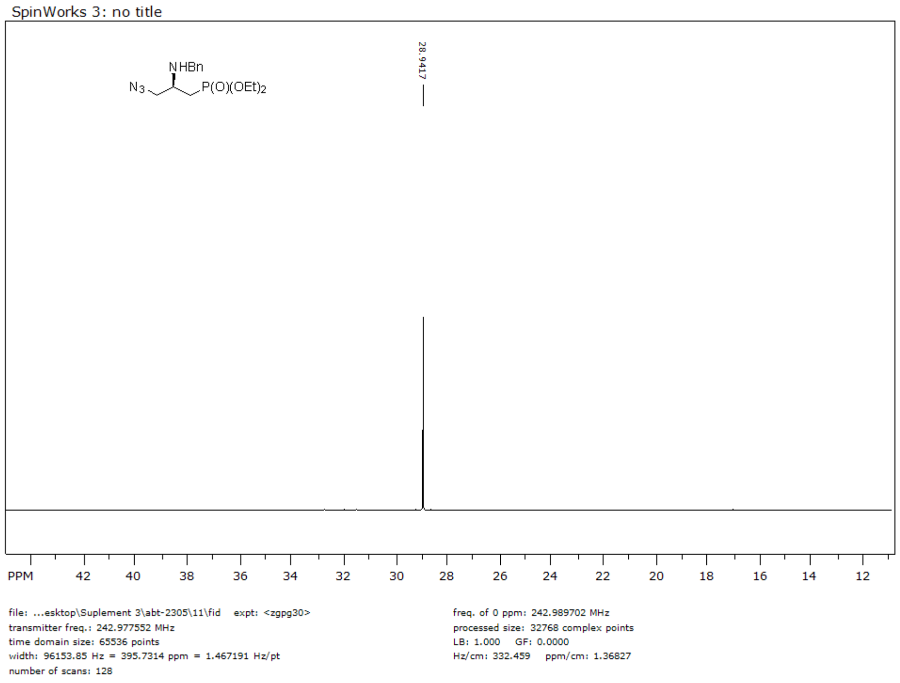

Figure S27. ^{1}H NMR Spectrum for (*S*)-**27** in $CDCl_3$

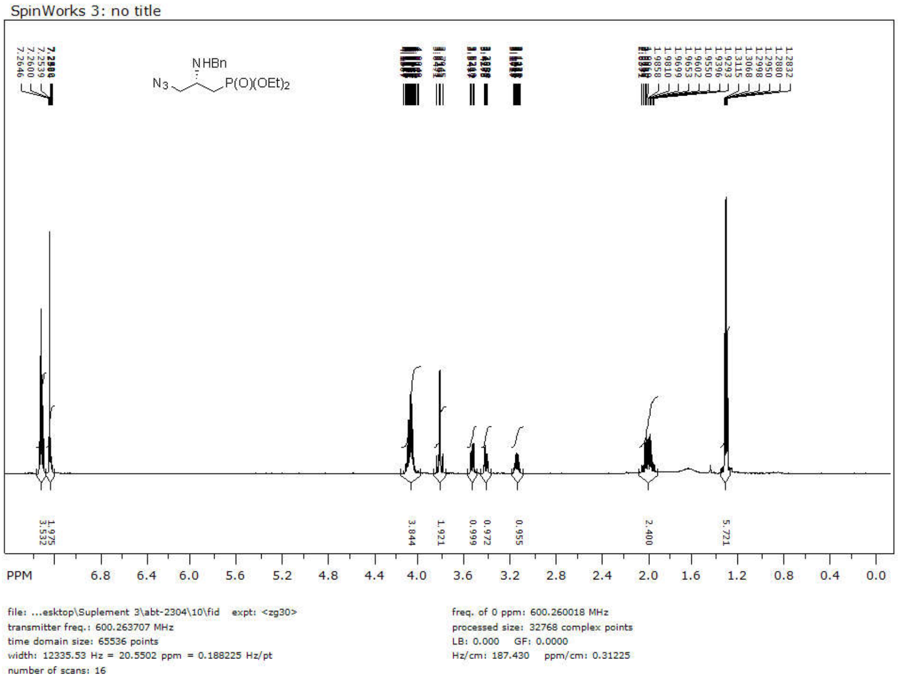

Figure S28. ^{31}P NMR Spectrum for (*S*)-**27** in $CDCl_3$

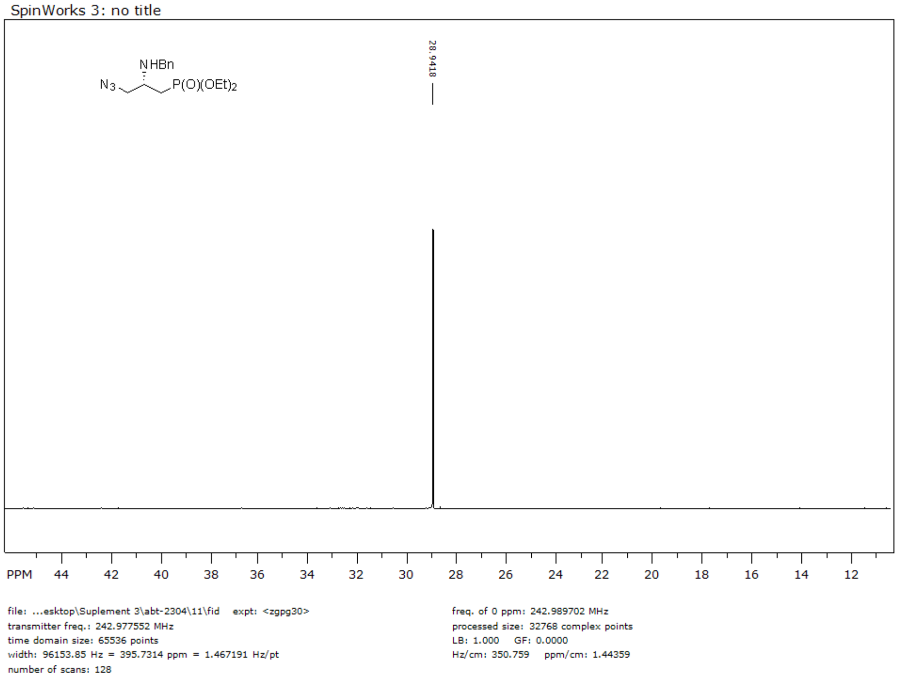

Figure S29. ^{1}H NMR Spectrum for racemic **28** in $CDCl_3$

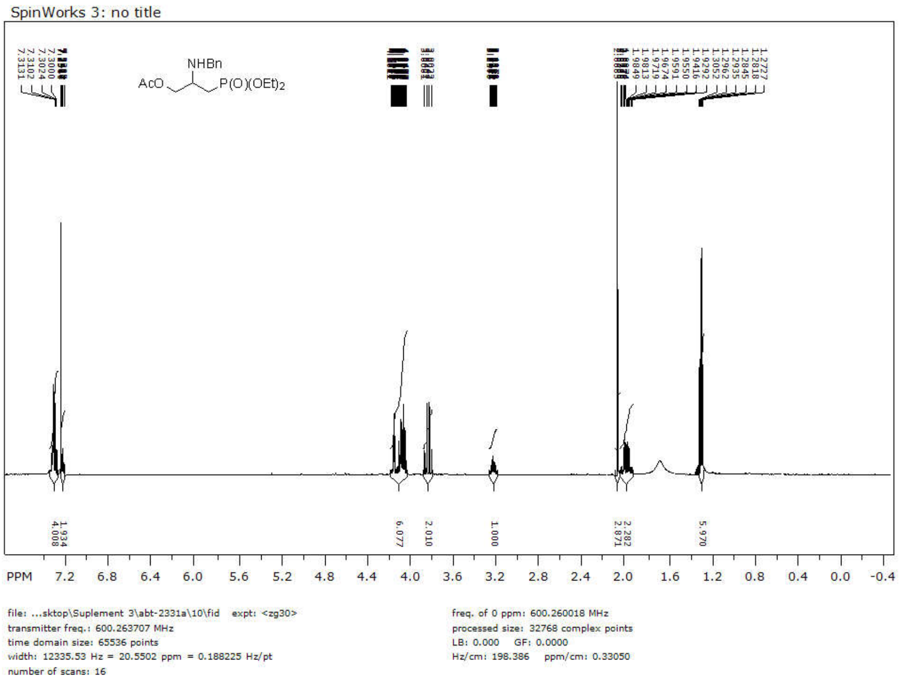

Figure S30. ^{13}C NMR Spectrum for racemic **28** in $CDCl_3$

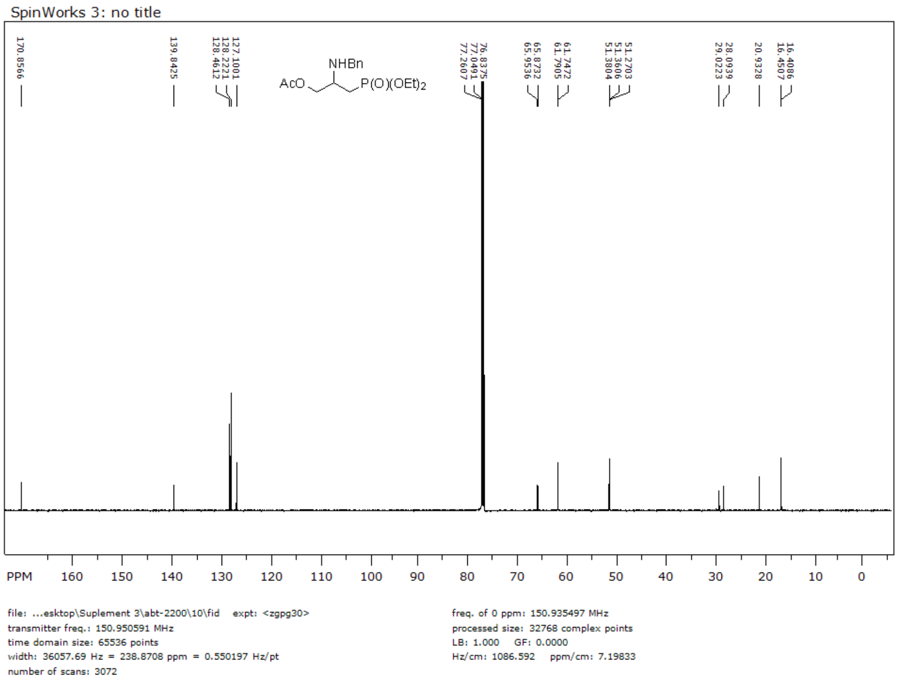

Figure S31. ^{31}P NMR Spectrum for racemic **28** in $CDCl_3$

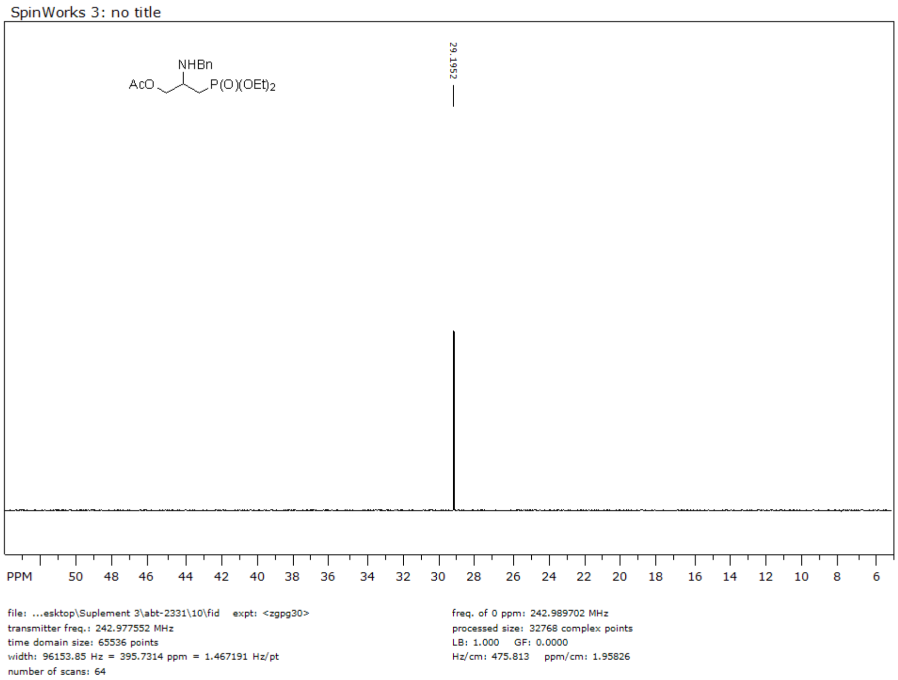

Figure S32. ^{1}H NMR Spectrum for (*R*)-**28** in $CDCl_3$

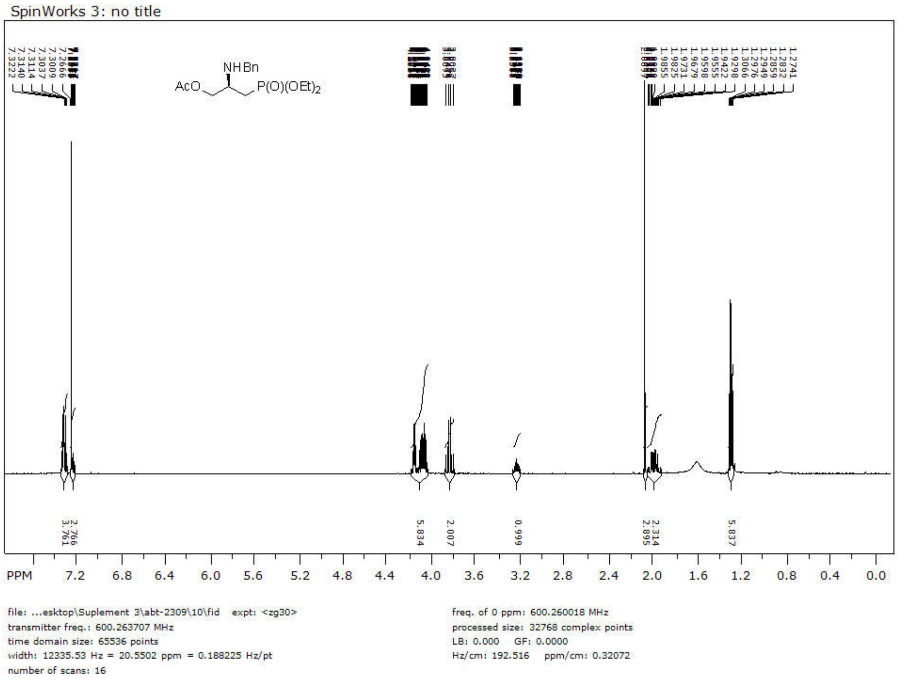

Figure S33. ^{31}P NMR Spectrum for (*R*)-**28** in $CDCl_3$

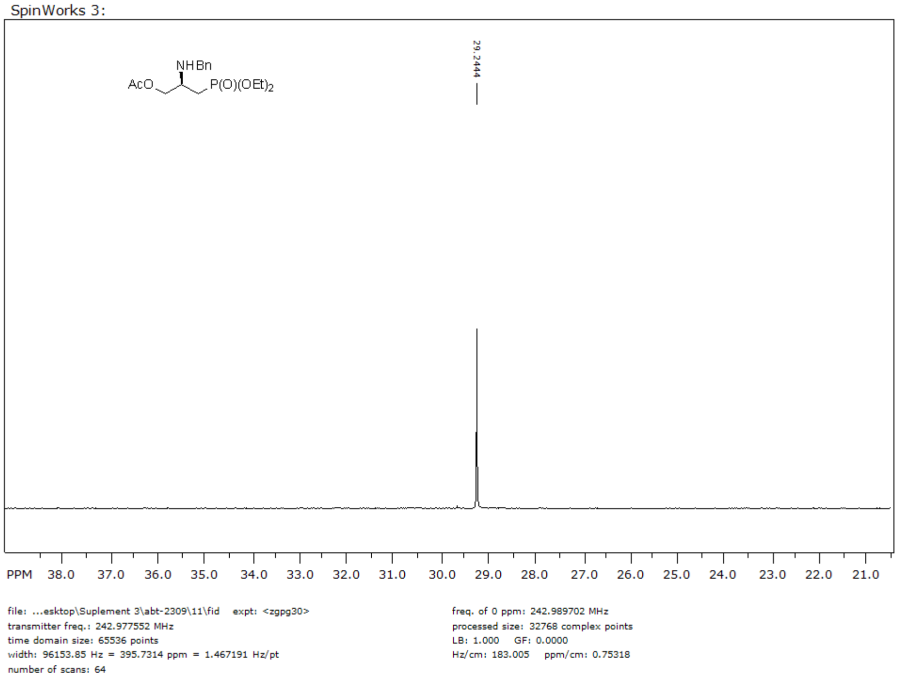

Figure S34. ^{1}H NMR Spectrum for (*S*)-**28** in $CDCl_3$

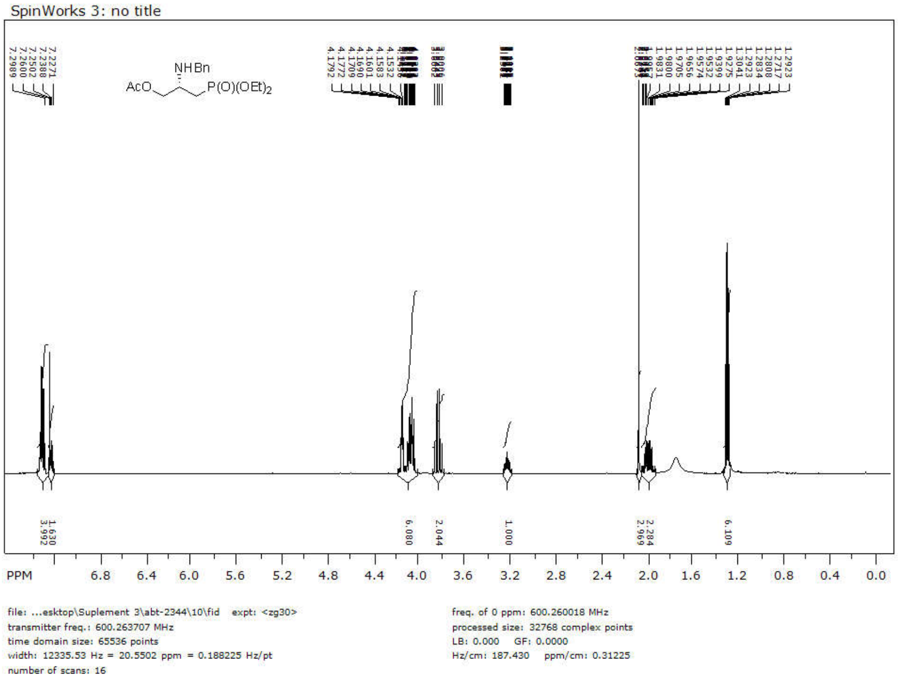

Figure S35. ^{31}P NMR Spectrum for (*S*)-**28** in $CDCl_3$

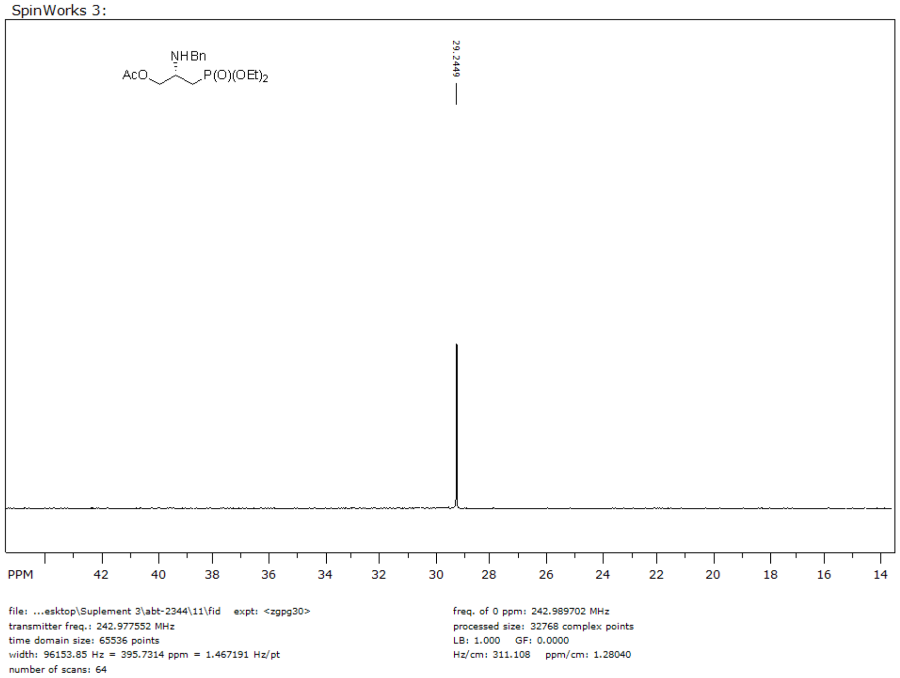

Figure S36. ^{1}H NMR Spectrum for racemic **29** in $CDCl_3$

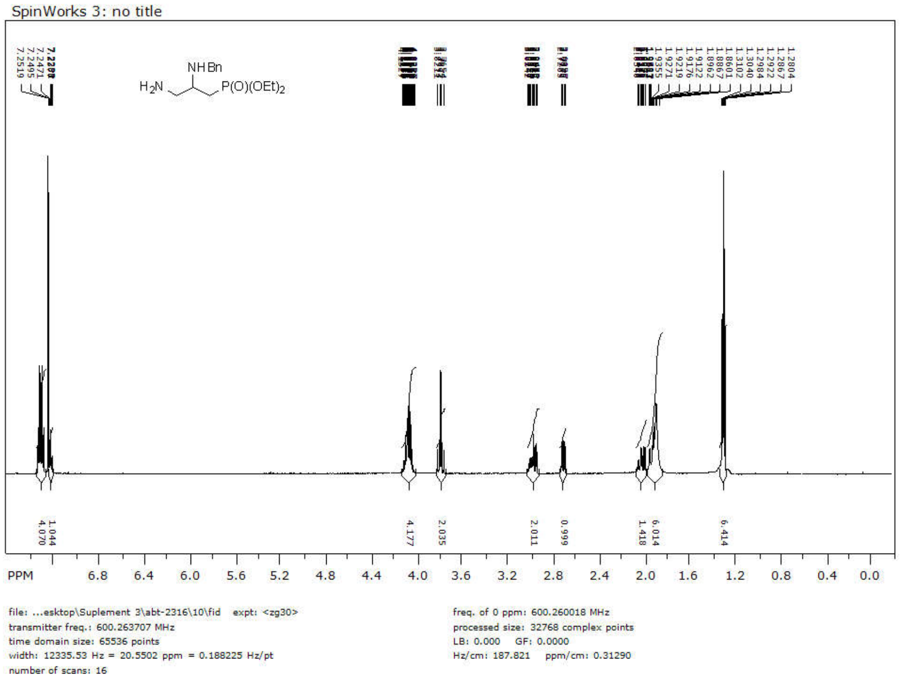

Figure S37. ^{13}C NMR Spectrum for racemic **29** in $CDCl_3$

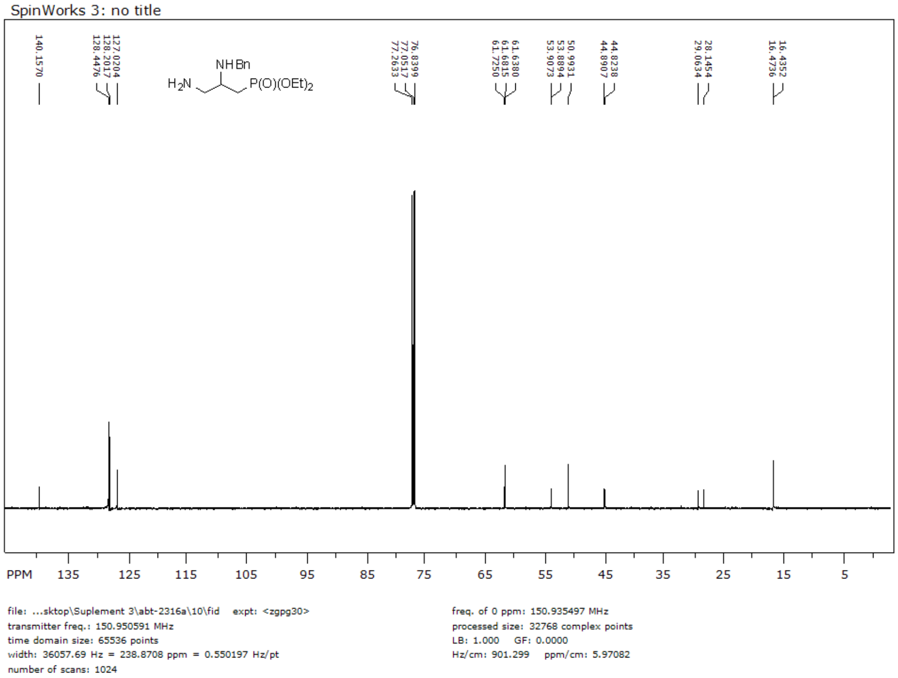

Figure S38. ^{31}P NMR Spectrum for racemic **29** in $CDCl_3$

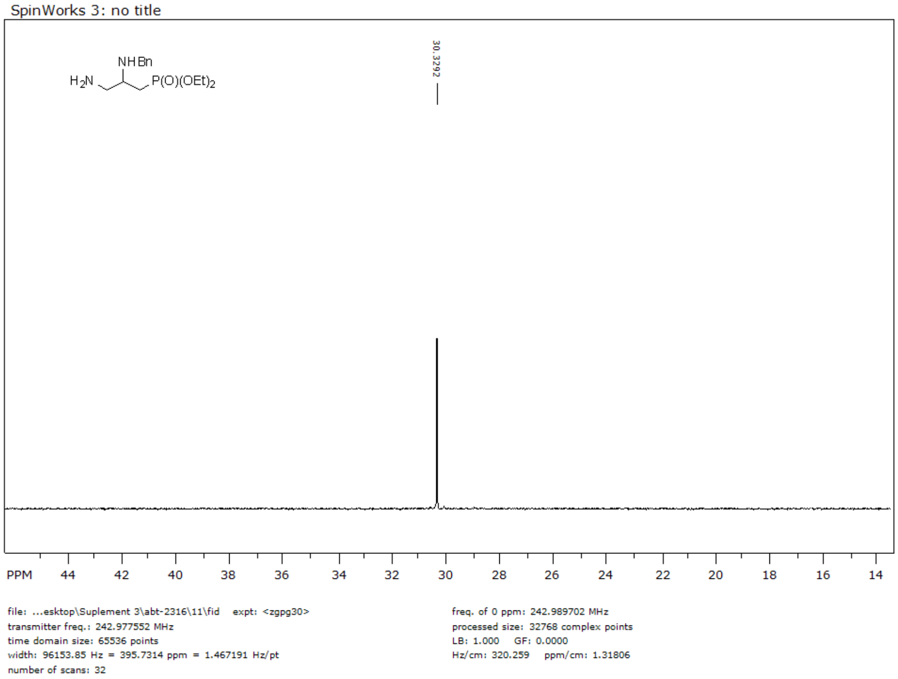

Figure S39. ^{1}H NMR Spectrum for (*R*)-**29** in $CDCl_3$

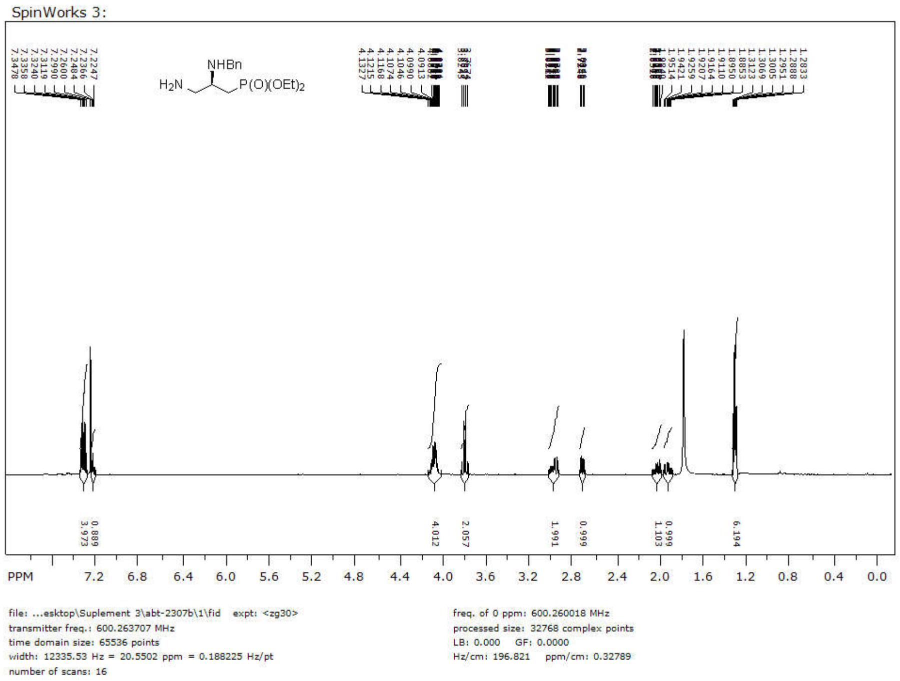

Figure S40. ^{31}P NMR Spectrum for (*R*)-**29** in $CDCl_3$

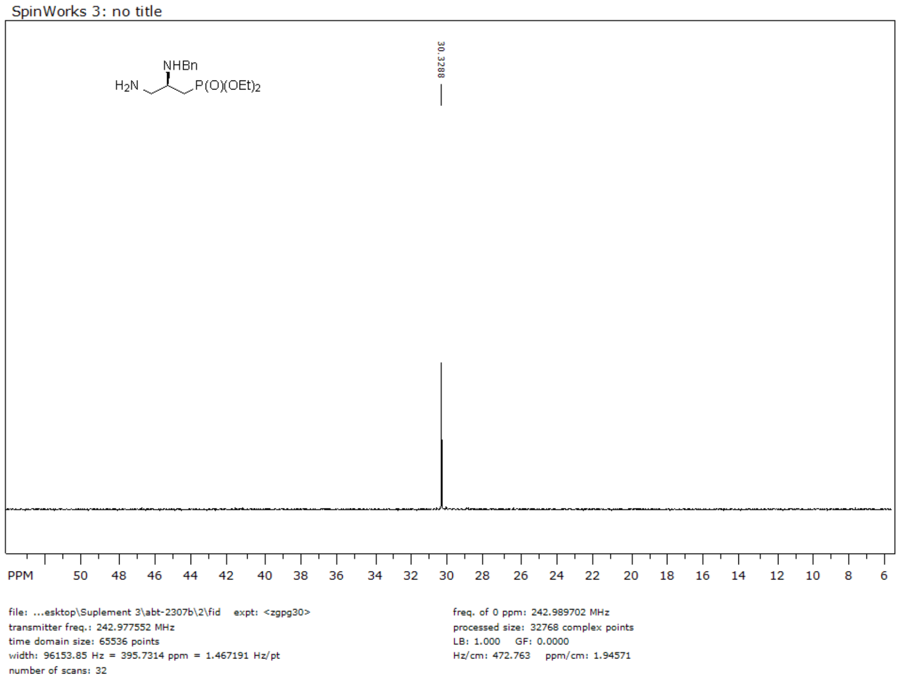

Figure S41. ^{1}H NMR Spectrum for (*S*)-**29** in $CDCl_3$

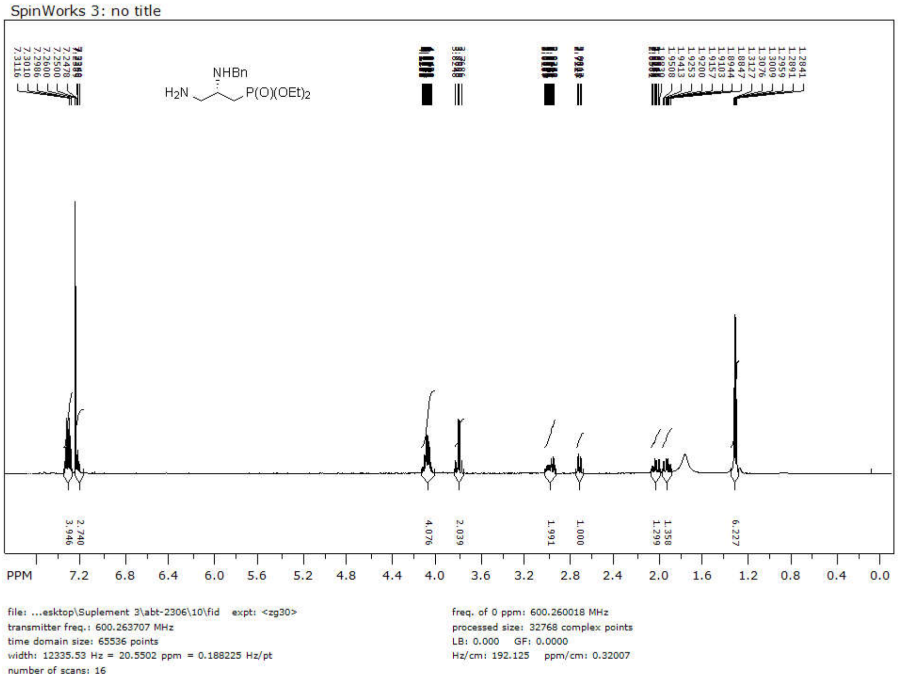

Figure S42. ^{31}P NMR Spectrum for (*S*)-**29** in $CDCl_3$

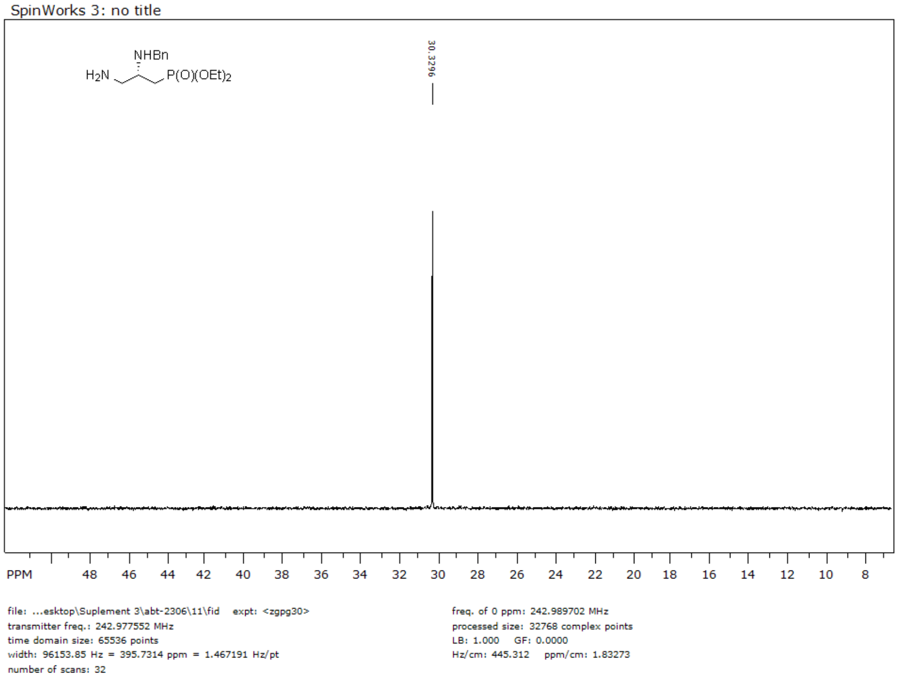

Figure S43. ^{1}H NMR Spectrum for racemic **34** in $CDCl_3$

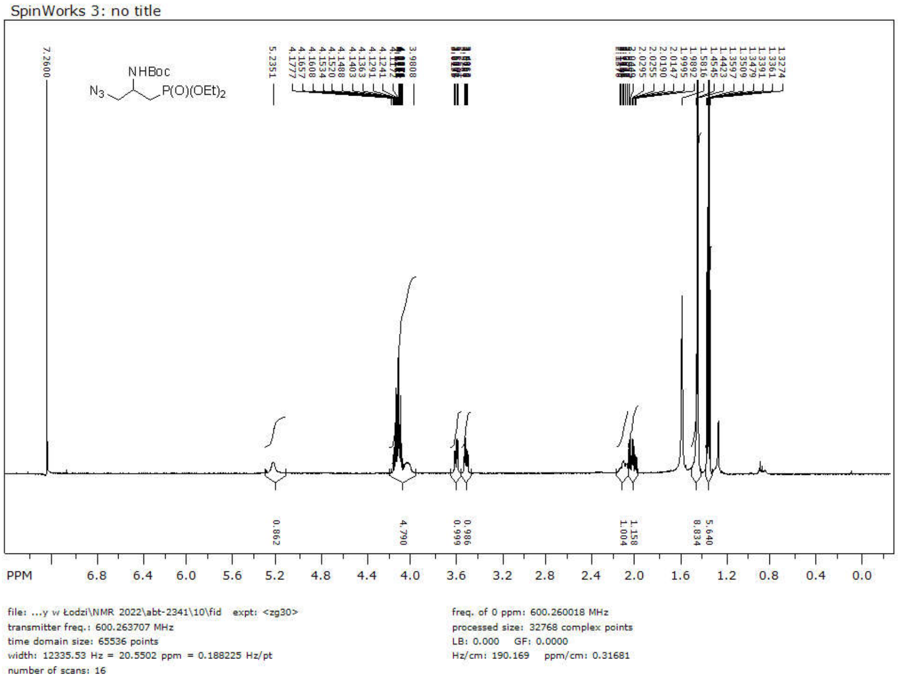

Figure S44. ^{13}C NMR Spectrum for racemic **34** in $CDCl_3$

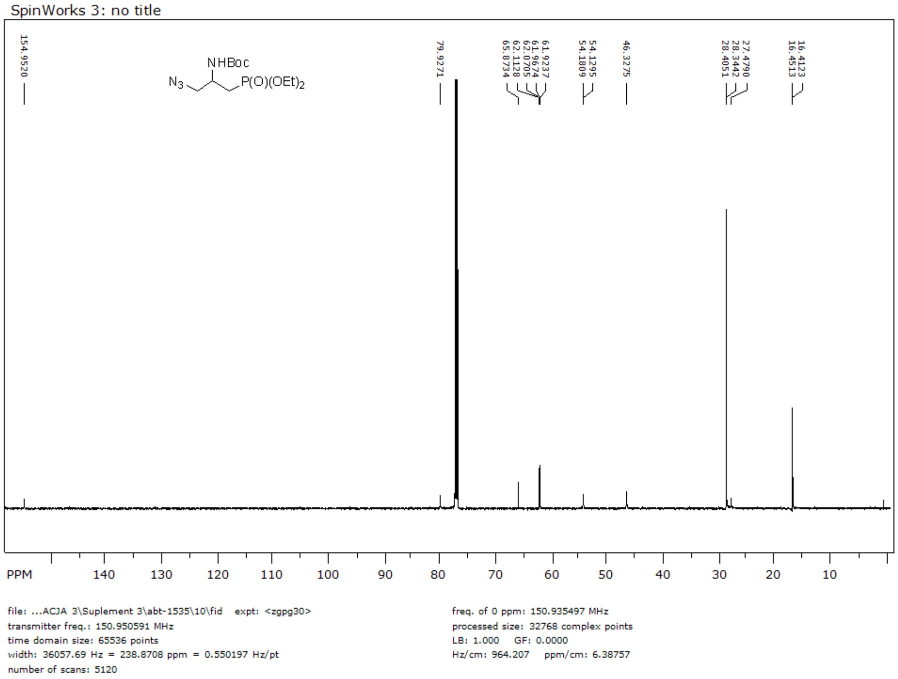

Figure S45. ^{31}P NMR Spectrum for racemic **34** in $CDCl_3$

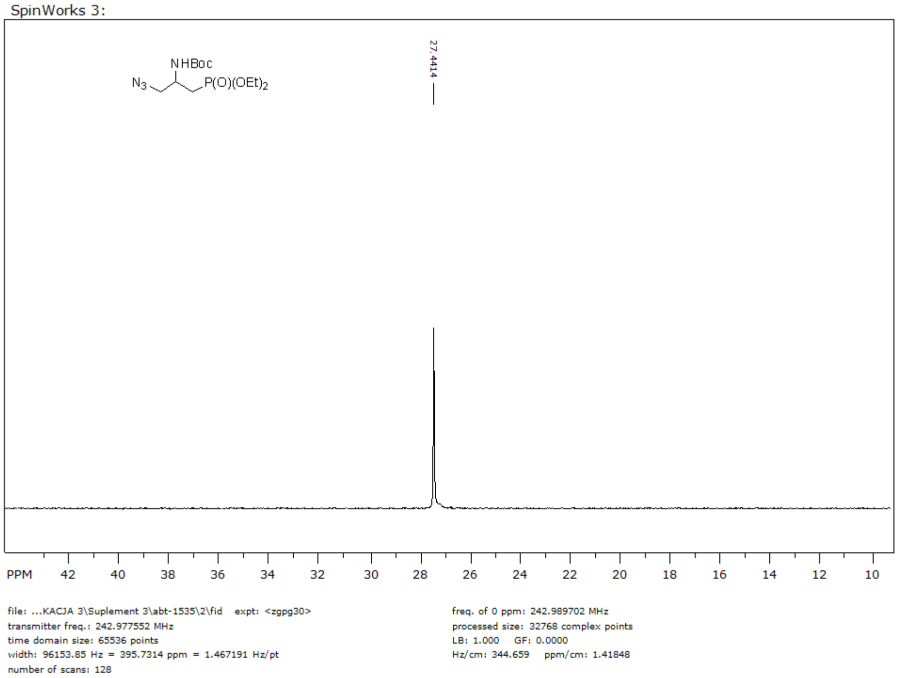

Figure S46. ^{1}H NMR Spectrum for racemic **35** in $CDCl_3$

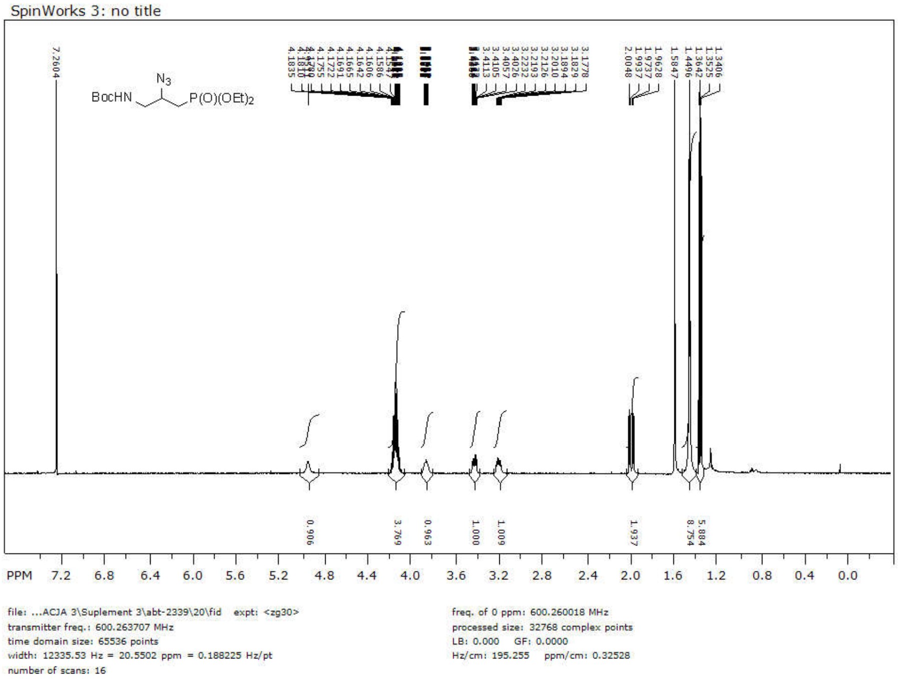

Figure S47. ^{13}C NMR Spectrum for racemic **35** in $CDCl_3$

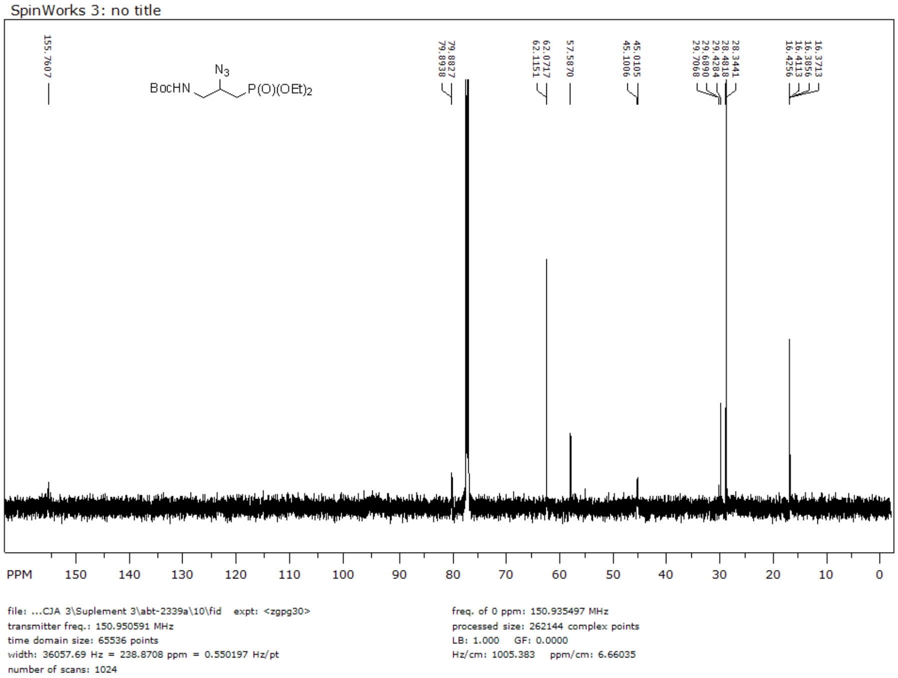

Figure S48. ^{31}P NMR Spectrum for racemic **35** in $CDCl_3$

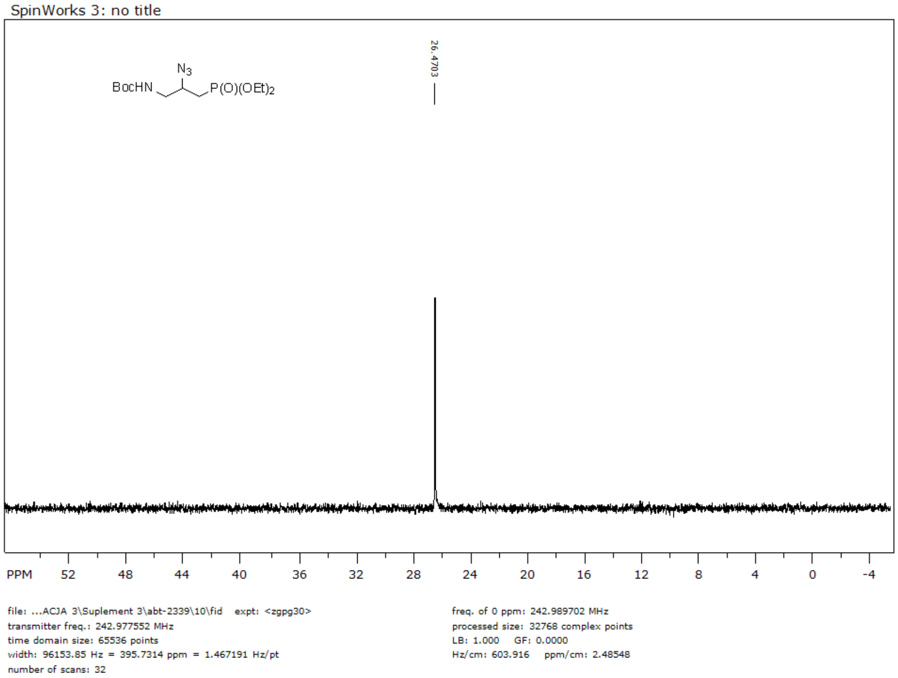

Figure S49. ^{1}H NMR Spectrum for (*E*)-**39** in $CDCl_3$

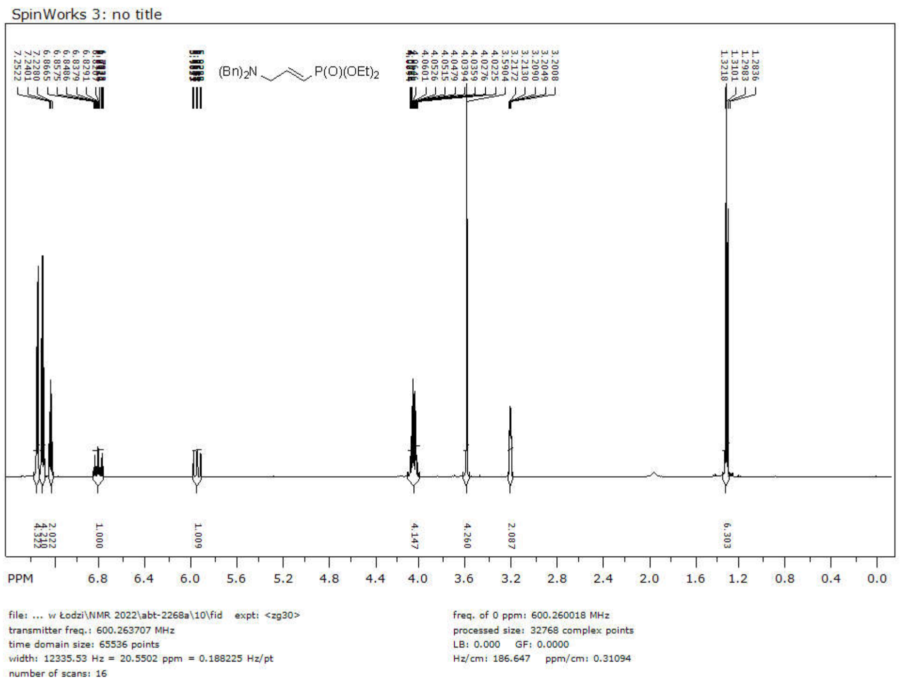

Figure S50. ^{13}C NMR Spectrum for (*E*)-**39** in $CDCl_3$

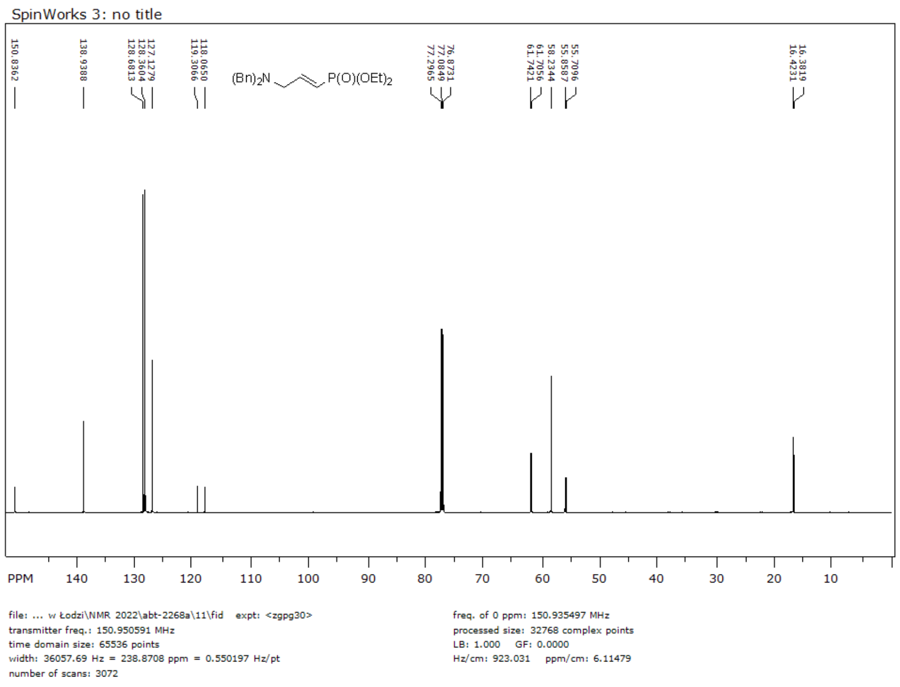

Figure S51. ^{31}P NMR Spectrum for (*E*)-**39** in $CDCl_3$

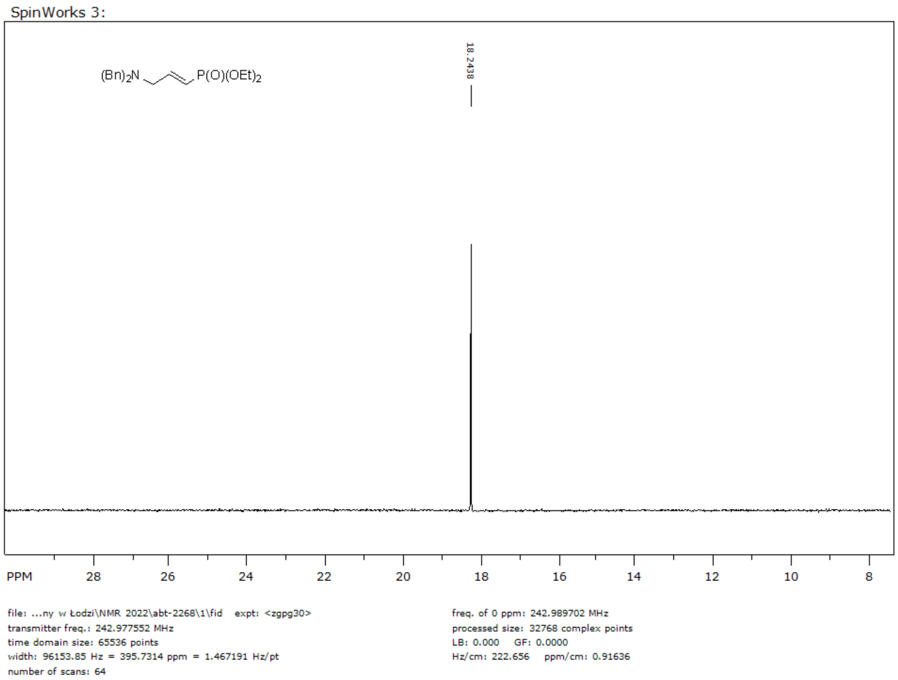

Figure S52. ^{31}P NMR Spectrum for crude mixture after reaction of racemic **30** with (*R*)-1-phenylethylamine in $CDCl_3$

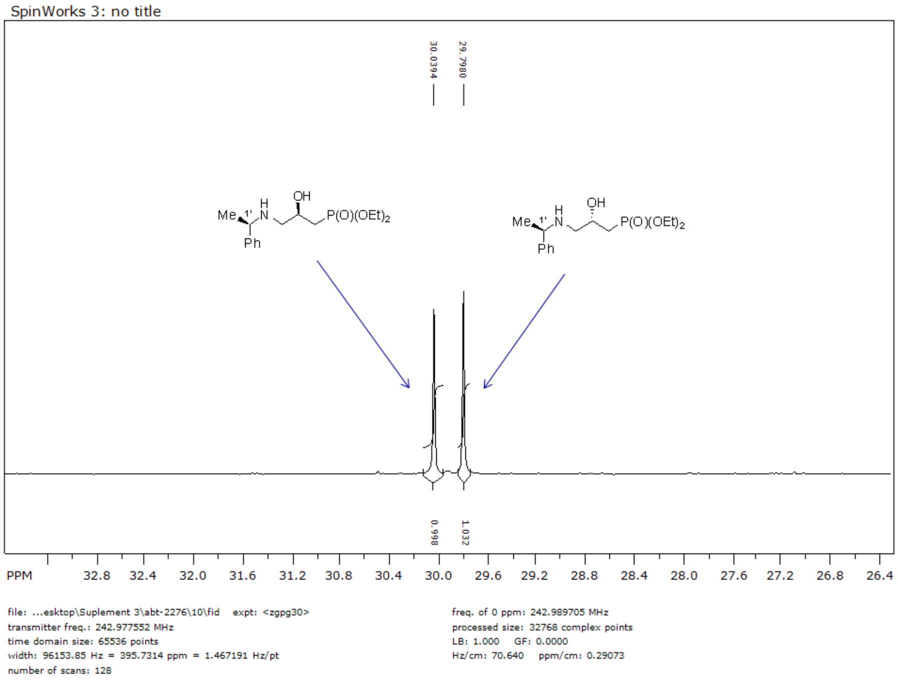

Figure S53. ^{31}P NMR Spectrum for crude mixture after reaction of (*S*)-**30** with (*R*)-1-phenylethylamine in $CDCl_3$

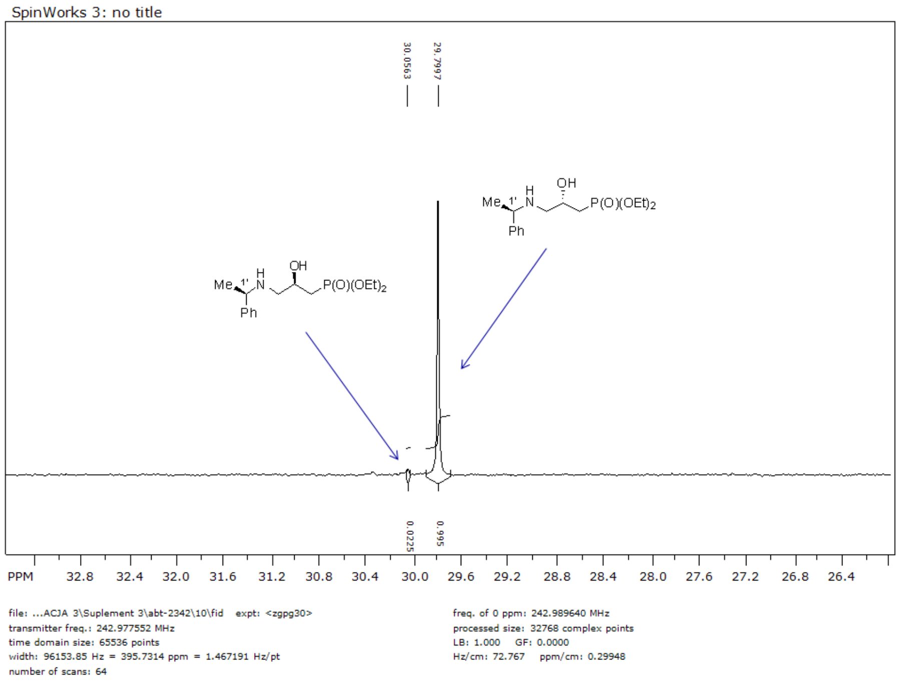

Figure S54. ^{31}P NMR Spectrum for crude mixture after reaction of (*R*)-**30** with (*R*)-1-phenylethylamine in $CDCl_3$

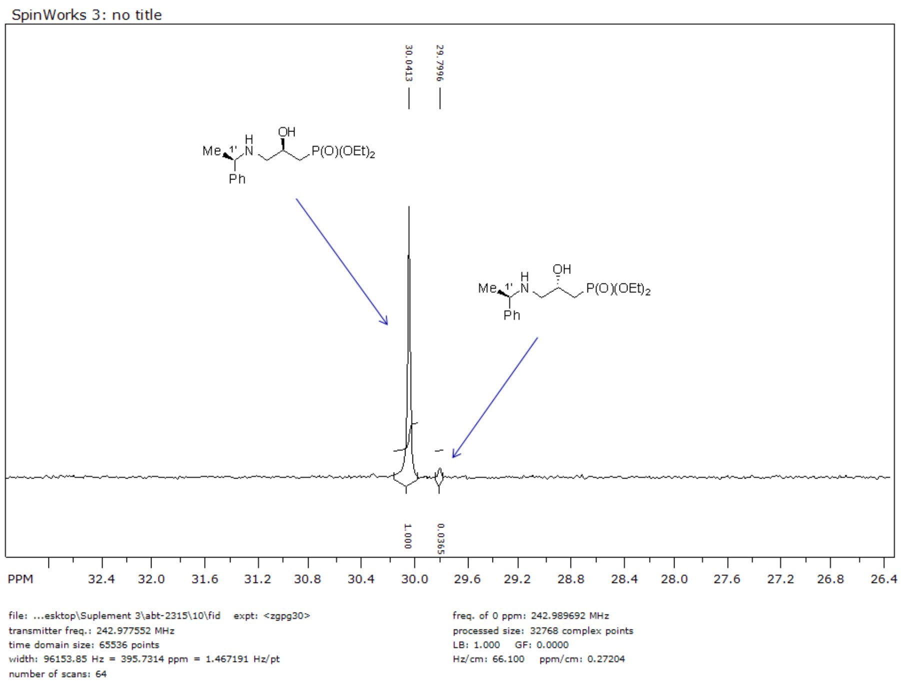

Figure S55. ^{31}P NMR Spectrum for mixture of crude (*S*)-**31** with 4 equiv. of quinine in $CDCl_3$

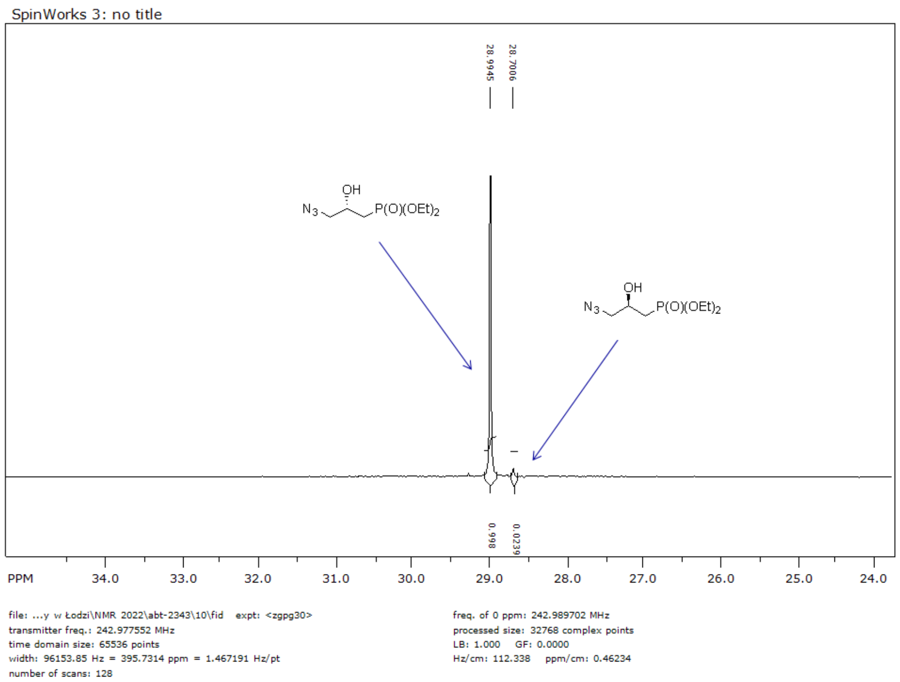

Figure S56. ^{31}P NMR Spectrum for mixture of crude (*R*)-**31** with 4 equiv. of quinine in $CDCl_3$

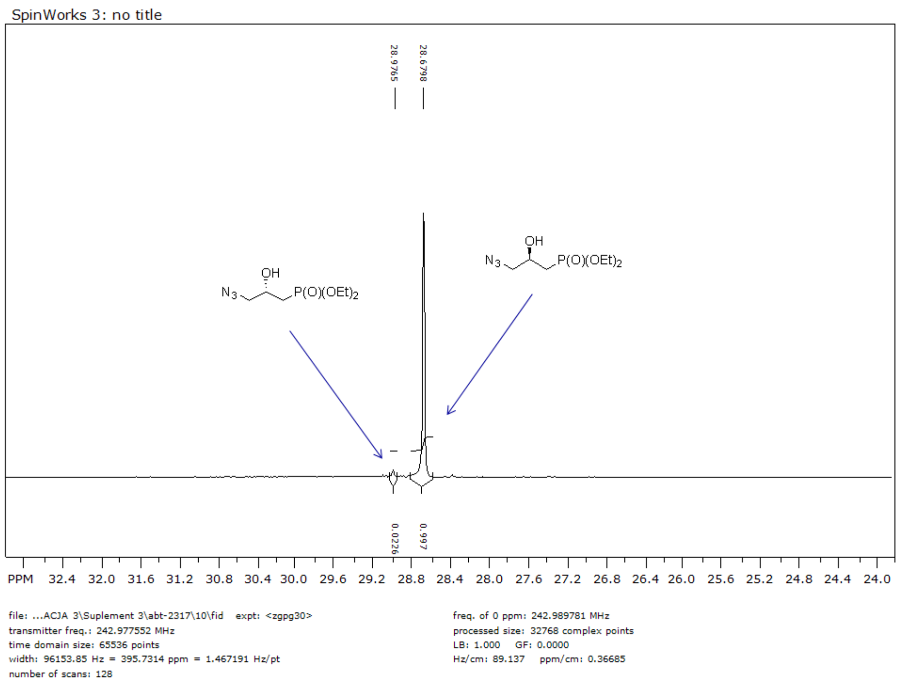

Figure S57. Analytical chromatogram for racemic **27**

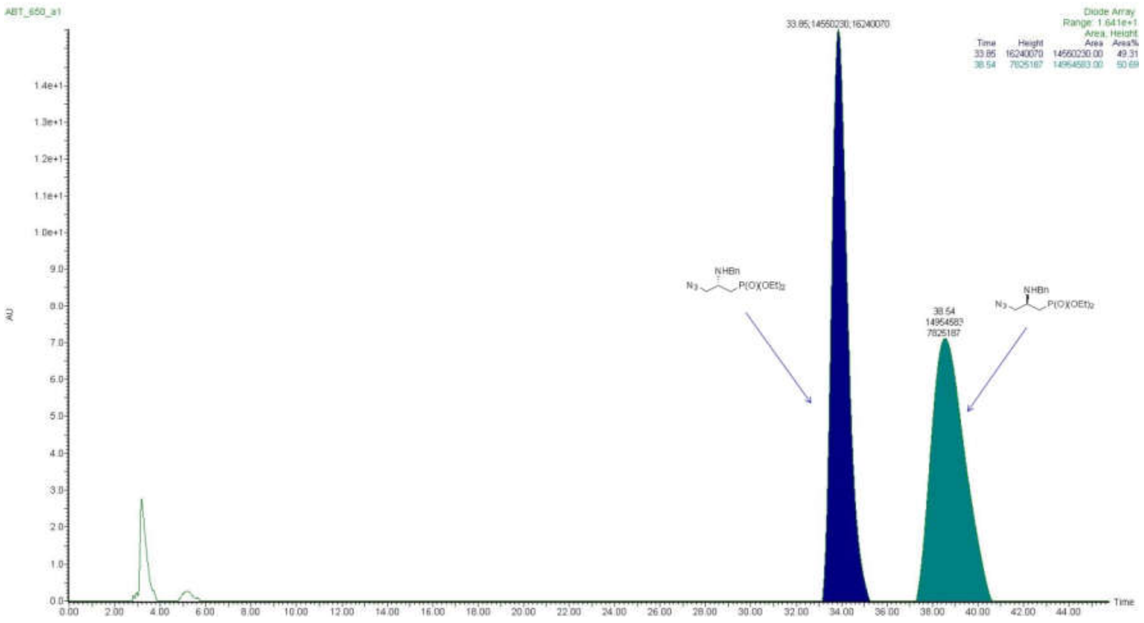

Figure S58. Analytical chromatogram for (*R*)-**27**

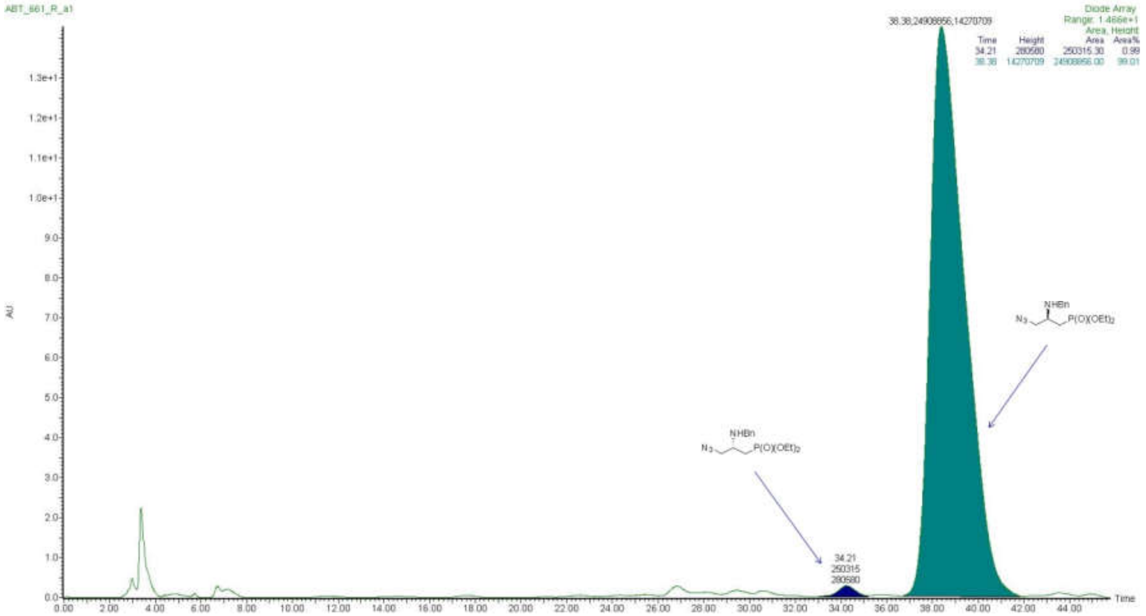

Figure S59. Analytical chromatogram for (*S*)-**27**

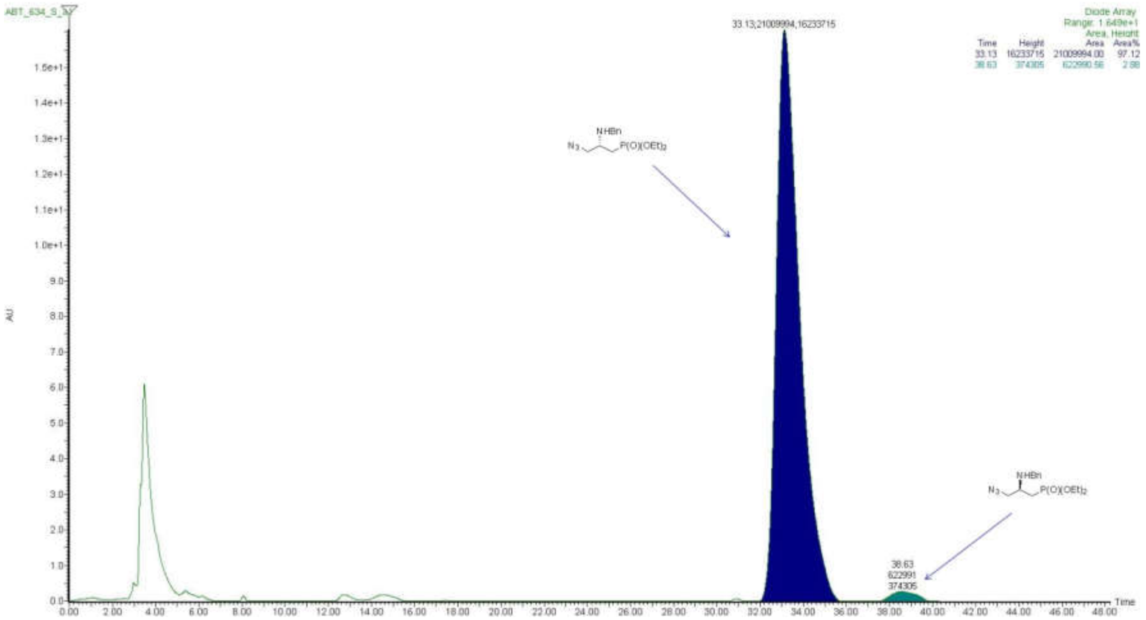

Figure S60. Analytical chromatogram for racemic **28**

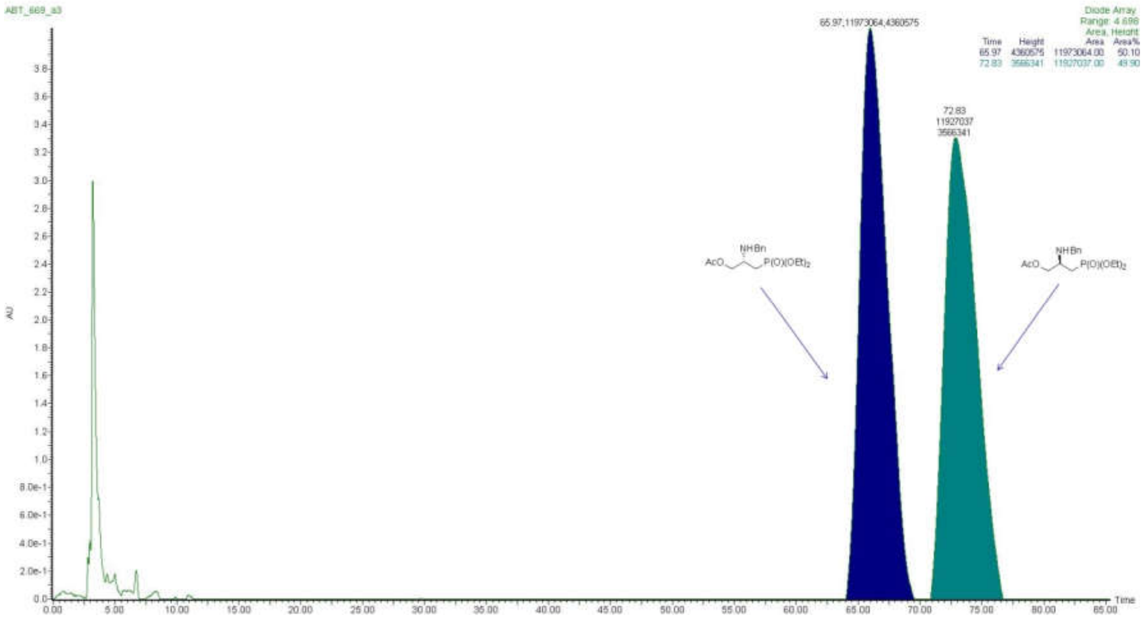

Figure S61. Analytical chromatogram for (*R*)-**28**

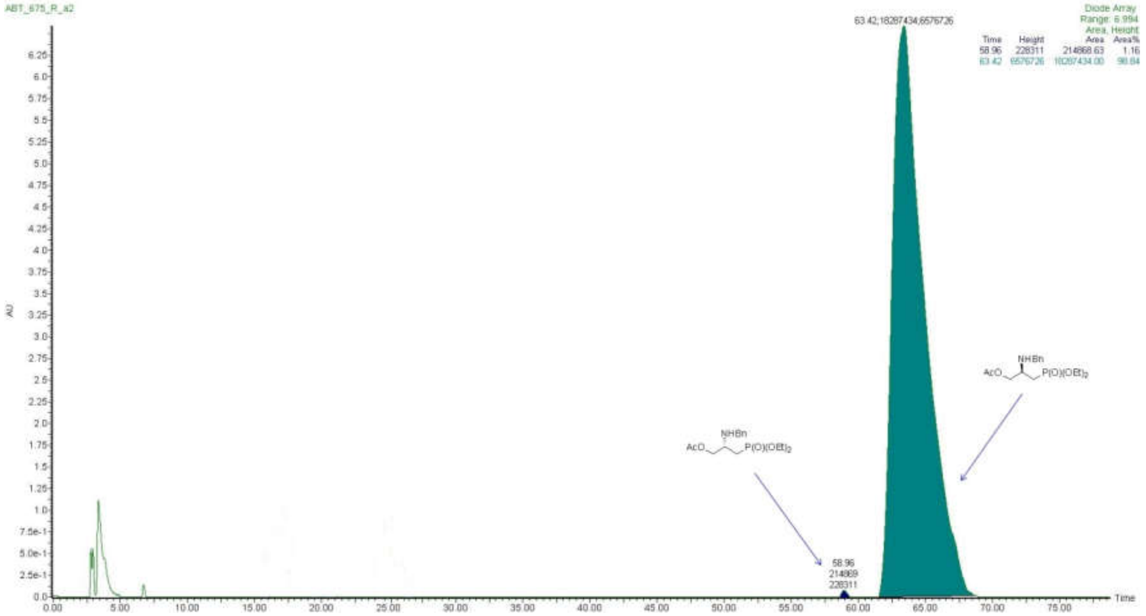

Figure S62. Analytical chromatogram for (*S*)-**28**

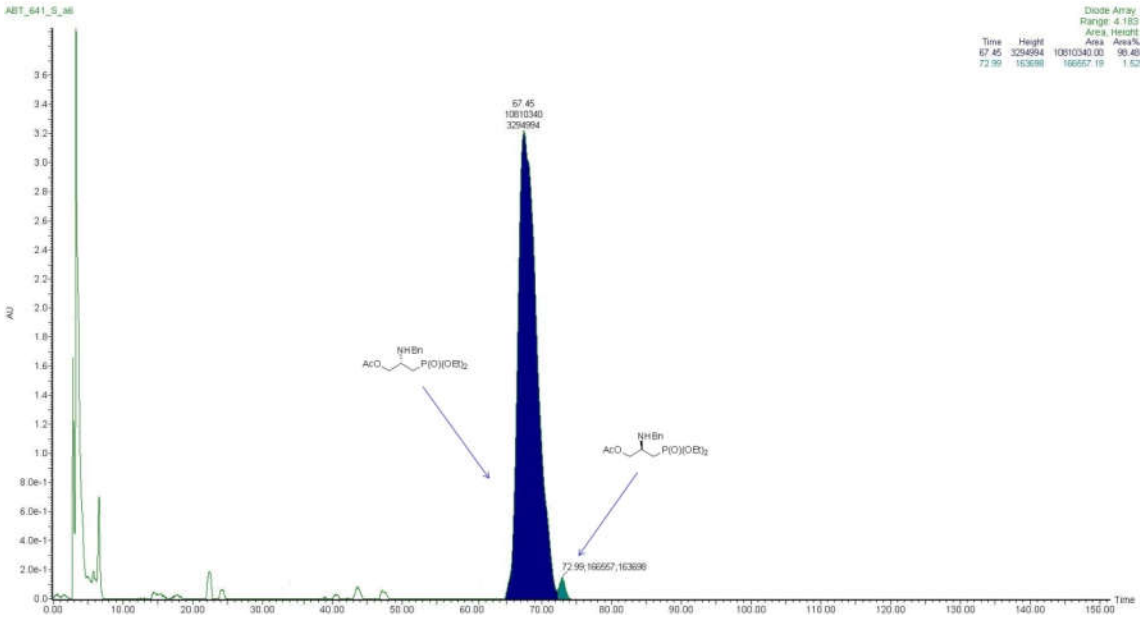

Supplement: Supplementary file 1 [file molecules-28-01466-s001.zip › molecules-2194273-supplementary.pdf]
